# Antiprotozoal activity of natural products from Nigerien plants used in folk medicine

**DOI:** 10.3389/fphar.2023.1190241

**Published:** 2023-06-23

**Authors:** Ozlem Sevik Kilicaslan, Sylvian Cretton, Estelle Hausmann, Luis Quirós-Guerrero, Soumana Karimou, Marcel Kaiser, Pascal Mäser, Philippe Christen, Muriel Cuendet

**Affiliations:** ^1^ School of Pharmaceutical Sciences, University of Geneva, Geneva, Switzerland; ^2^ Institute of Pharmaceutical Sciences of Western Switzerland, University of Geneva, Geneva, Switzerland; ^3^ Plantasav, Niamey, Niger; ^4^ Swiss Tropical and Public Health Institute, Allschwil, Switzerland; ^5^ University of Basel, Basel, Switzerland

**Keywords:** *Cassia sieberiana*, *Ziziphus mauritiana*, *Sesamum alatum*, neglected tropical diseases, malaria

## Abstract

In the course of the screening of plants from Niger for antiprotozoal activity, the methanol extract of *Cassia sieberiana*, and the dichloromethane extracts of *Ziziphus mauritiana* and *Sesamun alatum* were found to be active against protozoan parasites, namely *Trypanosoma brucei rhodesiense*, *Trypanosoma cruzi*, *Leishmania donovani* and/or *Plasmodium falciparum*. Myricitrin (**1**), quercitrin (**2**) and 1-palmitoyl-lysolecithin (**3**) were isolated from *C. sieberiana*. From *Z. mauritiana*, the three triterpene derivatives **13**, **15**, and **16** are described here for the first time. Their chemical structures were determined by 1D and 2D NMR experiments, UV, IR and HRESIMS data. The absolute configurations were assigned via comparison of the experimental and calculated ECD spectra. In addition, eight known cyclopeptide alkaloids (**4**, **5**, **7**–**12**), and five known triterpenoids (**6**, **14**, **17–19**) were isolated. The antiprotozoal activity of the isolated compounds, as well as of eleven quinone derivatives (**20–30**) previously isolated from *S. alatum* was determined *in vitro.* The cytotoxicity in L6 rat myoblast cells was also evaluated. Compound **18** showed the highest antiplasmodial activity (IC_50_ = 0.2 µm) and compound **24** inhibited *T. b. rhodesiense* with an IC_50_ value of 0.007 *µ*M. However, it also displayed significant cytotoxicity in L6 cells (IC_50_ = 0.4 µm).

## Introduction

Human protozoal diseases remain an important health issue, with significant morbidity and mortality, particularly in tropical and sub-tropical regions. Malaria, which is caused by parasites of the genus *Plasmodium*, is the most prevalent parasitic disease with approximately 247 million cases and almost 619,000 deaths per year, mostly affecting young children in Africa (WHO, 2022c). Moreover, human African trypanosomiasis, American trypanosomiasis, and leishmaniasis affect millions of people worldwide (WHO, 2022b). Trypanosomiasis and leishmaniasis are protozoan infections caused by kinetoplastids. *Trypanosoma cruzi* is responsible for American trypanosomiasis also known as Chagas disease, which affects approximately 7 million people annually in Central and South America (WHO, 2022a). Sleeping sickness or human African trypanosomiasis, caused by *T. brucei* subspecies*,* leads to about 1000 cases in Africa annually (Franco et al., 2022). Worldwide, it is estimated that up to 100,000 people are affected by *Leishmania donovani*, a protozoan parasite that causes visceral leishmaniasis (WHO, 2021). The drugs currently used to treat the four diseases are limited because of important side effects, variable efficacy depending on the phase of the disease, and emerging resistances.

Thirty-eight plants from Niger were selected based on their use in traditional medicine. Aerial and/or subterranean parts of each plant were successively extracted with dichloromethane (DCM), methanol (MeOH), and H_2_O to obtain a wide variety of metabolites of various polarities. The extracts were obtained and tested against four parasites, *T. b. rhodesiense*, *T. cruzi*, *L. donovani* and *P. falciparum.* The selection criteria for the phytochemical investigation of the biologically active extracts were their LC-MS profile and traditional use. Based on these criteria, the MeOH extract from the leaves of *Cassia sieberiana* DC., as well as the DCM extracts from the root bark and roots of *Ziziphus mauritiana* Lam. and *Sesamum alatum* Thonn., respectively, were selected for further studies.

*Cassia,* a large genus of the Fabaceae family, is widely distributed in tropical and subtropical regions and contains 37 species (POWO, 2022). *C. sieberiana* is a perennial tree native to Africa and is used in traditional medicine against fever, diarrhea, leprosy, dropsy, bilharzia and abdominal pain (Tamboura et al., 2005). The MeOH and benzene leaf extracts of *C. sieberiana* showed *in vitro* antiplasmodial activity (Aliyu et al., 2013). In addition, the DCM leaf extract of *C. sieberiana* displayed an *in vitro* antitrypanosomal activity against *T. b. rhodesiense* with an IC_50_ value of 25.8 *µ*g/mL (Hoet et al., 2004).

The genus *Ziziphus* (Rhamnaceae) is distributed in tropical and subtropical areas of the world, and comprises 70 species of deciduous or evergreen trees and shrubs (POWO, 2022). *Z. mauritiana* is traditionally used for the treatment of diarrhea (Dahiru et al., 2006), ulcers, vomiting, indigestion (Bunyapraphatsara and Chokechaijaroenporn, 1999), pulmonary ailments, dysentery and fever (Morton, 1987; Verheij and Coronel, 1991). The antiplasmodial activity of the hydroethanol leaf extract of *Z. mauritiana* was reported (Attemene et al., 2018). However, there is no previous report on its activity against *T. b. rhodesiense*, *T. cruzi* and *L. donovani*.

The *Sesamum* genus (Pedaliaceae) comprises 31 species, most of which are native to tropical Africa (POWO, 2022). *S. alatum*, a wild plant, has been reported for its traditional use against women infertility, vomiting, diarrhea, and fever (Eklu-Natey et al., 2012). Lignans and saponins were isolated from the aerial parts of *S. alatum* (Kamal-Eldin and Yousif, 1992; Potterat et al., 1992). The antiproliferative activity against multiple myeloma cells of the DCM root extract of *S. alatum* and of the eighteen isolated compounds was reported previously (Saraux et al., 2022).

## Materials and methods

### General experimental procedures

For UHPLC separations, LC-MS grade acetonitrile (MeCN) and water were purchased from Fisher (Waltham, MA, United States). For extraction, technical grade MeOH and DCM were obtained from Thommen-Furler (Rüti bei Büren, Switzerland). For fractionation and preparative separation, HPLC-grade MeCN was purchased from Fisher and water obtained by a Milli-Q water purification system (Labatec Pure Lab Ultra, Geneva, Switzerland). Formic acid 98% was purchased from Acros Organics (Geel, Belgium). Optical rotation values were obtained in MeOH using a JASCO P-1030 polarimeter (Easton, MD, United States) equipped with a sodium lamp (589 nm). The ECD spectra were recorded in MeOH on a JASCO J-815 CD spectrometer. The UV spectra were obtained in MeOH on a Perkin-Elmer Lambda-25 UV-vis spectrophotometer (Wellesley, MA, United States). IR spectra were recorded using a Perkin-Elmer Spectrum 100 spectrometer (Waltham, MA, United States). NMR spectra were obtained using a Bruker Avance III HD 600 MHz NMR spectrometer equipped with a QCI 5 mm cryoprobe and a Sample Jet automated sample changer (Bruker BioSpin, Rheinstetten, Germany). The residual DMSO-*d*_6_ signals (*δ*_H_ 2.50; *δ*_C_ 39.5) were used as internal standards for ^1^H and ^13^C NMR experiments, respectively. Chemical shifts (*δ*) are reported in parts per million and coupling constants (*J*) are reported in Hz. Spectra were analyzed by the Mestrenova 14.1.2 software (Santiago de Compostela, Spain). HRMS spectra were obtained using a Q Exactive Focus Hybrid quadrupole-orbitrap mass spectrometer (Thermo Scientific, Waltham, MA, United States) using electrospray ionization in positive and negative-ion modes. The instrument was programmed with spray voltage at 3.5 kV; sheath gas flow rate (N_2_) at 50 units; capillary temperature at 320°C; S lens RF level at 50 and probe heater temperature at 425°C. UHPLC-PDA-MS analyses were acquired using an Acquity UPLC I-class System (Waters, Milford, MA, United States) equipped with an Acquity PDA detector and connected to a Quattro Micro triple quadrupole mass spectrometer (Waters) with an ESI source operating in positive-ion mode. The system was controlled by Mass Lynx 4.1 software (Waters). The separation was achieved on a Kinetex UPLC EVO C_18_ column (100 × 2.1 mm i. d., 1.7 μm, Phenomenex, Torrance, CA, United States), using a gradient (MeCN and H_2_O both containing 0.1% formic acid) from 5% to 98% MeCN in 15 min, followed by an isocratic washing step at 98% MeCN for 2 min. After the washing step, the column was re-equilibrated with 5% MeCN for 2 min prior to the next injection. The flow rate was set to 0.5 mL/min, the column temperature to 40°C, and the injection volume was 2 μL. The UV absorbance was measured at 210 nm, and PDA absorption spectra were recorded between 190 and 500 nm (1.2 nm steps). The spray voltage was set at 2.6 kV; the sheath gas flow rate (N_2_) at 600 L/h; the capillary temperature was 350°C; the spectra (150–1000 Da) were recorded every 0.20 s. Fractionation and semi-preparative chromatography were conducted using an Armen Spot preparative chromatographic system (Interchim, Montluçon, France) equipped with a quaternary pump, a UV detector and a fraction collector. The fractionation was performed using two flash chromatography columns connected in series (PF-C_18_HQ/120 g, 15 μm, Interchim). A Kinetex EVO C_18_ column (250 × 21.2 mm, 5 μm, Phenomenex) and an X-Select C_18_ column (250 × 19.0 mm, 5 μm, Waters) were used for semi-preparative HPLC separations. The fractions were analyzed by using an Acquity UPLC System (Waters) with an Acquity BEH C_18_ column (50 × 2.1 mm i.d., 1.7 μm, Waters).

### ECD computational details

The determination of the absolute configuration of compounds **15** and **16** was done by comparing the calculated and experimental ECD spectra. The structures proposed by 1D and 2D NMR experiments were used as a base to perform a conformational search using MMFF94s force field by Spartan Student v7 (Wavefunction, Irvine, CA, United States). Based on the results, the 20 isomers with lowest energy were successively optimized using PM3 and B3LYP/6-31G (d, p) basis sets with the CPCM model in MeOH in Gaussian 16 software (^©^ 2015–2022, Gaussian Inc., Wallingford, CT, United States). All optimized conformers in each step were checked to avoid imaginary frequencies. All optimized conformers were submitted to Gaussian 16 software for ECD calculations, using TD-DFT B3LYP/def2svp as basis set with a CPCM model in MeOH. The calculated ECD spectra were generated in SpecDis1.71 software (Berlin, Germany) based on Boltzmann weighing average. The computation in Gaussian was performed at the University of Geneva on the Baobab cluster, https://plone.unige.ch/distic/pub/hpc/baobab_en).

### Antiparasitic and cytotoxicity assays

The *in vitro* activity was assessed on *T. b. rhodesiense* (STIB900, trypomastigotes), *T. cruzi* (Tulahuen C2C4, intracellular amastigotes), *L. donovani* (MHOM-ET-67/L82, axenically grown amastigotes), *P. falciparum* (NF54, intraerythrocytic), and L6 cells (ATCC, CRL-1458™, rat skeletal myoblasts) as previously described (Bernal et al., 2020). The activities were determined in serial dilution assays with 72 h compound exposure, respectively 96 h for intracellular *T. cruzi*. Results are expressed in µg/mL for extracts and in µM for pure compounds. The IC_50_ values were determined for the compounds only when they were <50 µM.

### Plant material

*Cassia sieberiana* leaves, *Ziziphus mauritiana* root bark and *Sesamum alatum* roots were collected in November 2017 in Gabi, Niger. The identification was confirmed by Prof. Mahamane Saadou at the University of Maradi. Voucher specimens were deposited at the University of Geneva (no 2016020-L, 2016018-SB and 89128, respectively).

### Extraction

Air-dried and powdered *C. sieberiana* leaves (760 g) and *Z. mauritiana* root bark (270 g) were extracted at room temperature by maceration using MeOH and DCM (1:10, m/v), respectively (3 × 24 h). After filtration, extracts were evaporated under reduced pressure at 40°C to yield 12.2 g and 3.7 g of crude extracts of *C. sieberiana* leaves and *Z. mauritiana* root bark respectively. *S. alatum* roots were extracted as previously described (Saraux et al., 2022).

### Isolation

The MeOH leaf extract of *C. sieberiana* (6 g) was mixed with 16 g of Celite 577 (Fluka, Buchs, Switzerland) and introduced into a cartridge for a dry load injection. The fractionation was performed with a linear gradient mode of 20%–100% MeCN containing 0.1% formic acid over 120 min and then 100% MeCN for 30 min. The flow rate was set to 20 mL/min, and UV detection was performed at 220 nm. The separation yielded 214 fractions that were combined into 24 fractions according to their chromatographic profiles. Fraction 1 yielded myricitrin (**1**, 6 mg) and fraction 5 afforded quercitrin (**2,** 10 mg). Fractions 15 and 16 were subjected to semi-preparative HPLC using an X-Select C_18_ column (250 × 19.0 mm, 5 µm) using a linear gradient of 30%–80% MeCN containing 0.1% formic acid over 50 min and then up to 100% MeCN in 30 min to afford 1-palmitoyl-lysolecithin (**3**, 2 mg). The flow rate was set to 20 mL/min, and UV detection was performed at 220 nm.

The dichloromethane extract from *Z. mauritiana* root bark (3.7 g) was mixed with 8 g of Celite 577 and introduced into a cartridge for a dry load injection. Fractionation was performed using two flash chromatography columns connected in series (PF-C_18_HQ/120 g, 15 µm) with a linear gradient mode of 10%–100% MeCN containing 0.1% formic acid over 170 min and then 100% MeCN for 55 min. The flow rate was set to 20 mL/min, and UV detection was performed at 280 nm. The separation yielded 220 fractions that were combined into 27 fractions according to their chromatographic profiles. Fraction 4 yielded mauritine F (**4**, 1.5 mg), fraction 5 gave mauritine A (**5**, 0.8 mg) and fraction 20 gave ceanothic acid (**6**, 93.3 mg). Fractions 6, 9, 10, 22, 23, 25, and 27 were selected for further purification using a semi-preparative HPLC with a Kinetex EVO C_18_ column (250 × 21.2 mm, 5 µm) with MeCN/H_2_O/0.1% formic acid. The flow rate was set to 20 mL/min and UV absorbance was measured at 210 nm. Fraction 6 was subjected to a separation using a gradient of 15%–22% MeCN over 80 min, which yielded sanjoinine B (**7**, 1.9 mg), lotusanine A (**8**, 2.8 mg), nummularine K (**9**, 0.8 mg), amphibine H (**10**, 0.6 mg), and *N*-demethylamphibine H (**11**, 0.7 mg). Fractions 9 and 10 were separated using a gradient of 25%–40% MeCN in 80 min and gave mauritine M (**12**, 1.3 mg). Fractions 22 and 23 were fractionated using a gradient of 45%–65% MeCN in 80 min and yielded 3-dehydroxyceanothan-28*β*-19*β*-olide (**13**, 1.8 mg) from fraction 22. Fraction 23 afforded colubrinic acid (**14**, 1.4 mg) and (1*R*)-3-*O*-benzoyl ceanothic acid (**15**, 11.7 mg). Fraction 25 afforded (1*S*)-3-*O*-benzoyl ceanothic acid (**16**, 2.2 mg), ceanothenic acid (**17**, 0.7 mg) and 3-*O*-*trans-p*-coumarylalphitolic acid (**18**, 1.3 mg) using a gradient of 50%–55% MeCN in 80 min. Fraction 27 was separated using a gradient of 60%–65% MeCN in 80 min and gave betulinic acid (**19**, 2.6 mg).

Compounds **20**–**30** were isolated from the roots of *S. alatum* by liquid chomatography and characterized by spectral methods as previously described (Saraux et al., 2022).

Compound **13**: white, amorphous solid; ${\left[\alpha \right]}_{D}^{25}$ −110.2 (*c* 0.1, MeOH); UV (MeOH) *λ*_max_ (log *ε*), 231 (3.22), 270 (2.44) nm; ^1^H and ^13^C NMR, see Table 1; HRESIMS *m/z* 483.3123 ([M–H])^-^ (calcd for C_30_H_43_O_5_, 483.3105).

**TABLE 1:** ^1^H NMR (DMSO-*d*_*6*_, 600 MHz) and ^13^C NMR (DMSO-*d*_*6*_, 150 MHz) data of compounds **13**, **15**, **and 16**.

	13		15		16	
Position	*δ*_H_ (*J* in Hz)	*δ*_C_, type	*δ*_H_ (*J* in Hz)	*δ*_C_, type	*δ*_H_ (*J* in Hz)	*δ*_C_, type
**1**	2.23, m	61.5, CH	2.54, s	63.2, CH	2.67, d (7.5)	59.4, CH
**2**	-	174.1, C	-	175.2, C	-	172.2, C
**3**	3.95, d (7.6)	81.7, CH	5.21, s	85.3, CH	5.31, d (7.5)	84.1, CH
**4**	-	42.1, C	-	42.8, C	-	42.2, C
**5**	^a^	^a^	1.70, m	55.9, CH	1.05, m	60.9, CH
**6**	1.25, m	17.4, CH_2_	1.37, m	17.9, CH_2_	1.35, m	17.3, CH_2_
	1.36, m		1.72, m		1.45, m	
**7**	1.29, m	34.3, CH_2_	1.34, m	33.6, CH_2_	1.34, m	33.7, CH_2_
**8**	-	41.4, C	-	41.6, C	-	42.2, C
**9**	1.40, m	49.4, CH	1.66, m	44.2, CH	1.50, m	48.6, CH
**10**	-	46.6, C	-	48.6, C	-	46.6, C
**11**	1.23, m	24.7, CH_2_	1.37, m	23.9, CH_2_	1.32, m	23.6, CH_2_
	1.58, m		1.55, m		1.43, m	
**12**	1.50, m	28.3, CH_2_	0.96, m	25.0, CH_2_	1.02, m	24.7, CH_2_
	1.80, m		1.56, m		1.61, m	
**13**	2.23, m	33.6, CH	2.23, td (3.0, 12.2)	37.9, CH	2.23, td (3.4, 12.3)	37.4, CH
**14**	-	40.9, C	-	42.6, C	-	40.9, C
**15**	1.14, m	27.6, CH_2_	1.09, m	29.4, CH_2_	1.10, m	29.4, CH_2_
	1.40, m		1.44, m		1.44, m	
**16**	1.32, m	33.4, CH_2_	1.37, m	31.7, CH_2_	1.36, m	31.7, CH_2_
	2.17, m		2.12, dt (3.2, 12.5)		2.12, dt (3.2, 12.3)	
**17**	-	53.1	-	55.4, C	-	55.2, C
**18**	1.95, m	54.4	1.52, m	48.5, CH	1.52, m	48.5, CH
**19**	-	91.8, C	2.94, td (4.8, 10.6)	46.5, CH	2.94, td (4.7, 10.4)	46.6, CH
**20**	-	140	-	150.3, C	-	150.4, C
**21**	1.51, m	22.1, CH_2_	1.30, m	30.1, CH_2_	1.31, m	30.0, CH_2_
	1.81, m		1.81, m		1.81, m	
**22**	1.46, m	^a^	1.43, m	36.4, CH_2_	1.43, m	36.4, CH_2_
			1.80, m		1.81, m	
**23**	0.93, s	31.5, CH_3_	1.17, s	30.1, CH_3_	1.15, s	30.4, CH_3_
**24**	0.76, s	19.2, CH_3_	0.91, s	19.6, CH_3_	0.76, s	19.0, CH_3_
**25**	1.06, s	14.08, CH_3_	1.07, s	18.1, CH_3_	1.24, s	18.9, CH_3_
**26**	0.81, s	15.5, CH_3_	0.91, s	16.2, CH_3_	0.88, s	16.0, CH_3_
**27**	0.86, s	13.5, CH_3_	0.93, s	14.4, CH_3_	0.97, s	14.5, CH_3_
**28**	-	178.0, C	-	177.2, C	-	176.9, C
**29**	1.76, s	19.1, CH_3_	1.65, s	19.1, CH_3_	1.65, s	19.0, CH_3_
**30**	4.93, br s	111.5, CH_2_	4.58, br s	109.6, CH_2_	4.57, br s	109.7, CH_2_
	5.13, br s		4.69, br s		4.70, br s	
**1′**				133.5, C		133.3, C
**2′**			7.94, dd (1.2, 8.3)	129.1, CH	7.92, dd (1.4, 8.3)	129.1, CH
**3′**			7.54, t (7.7)	128.9, CH	7.54, t (7.8)	128.8, CH
**4′**			7.67, t (7.6)	133.4, CH	7.66, t (7.6)	133.3, CH
**5′**			7.54, t (7.7)	128.9, CH	7.54, t (7.8)	128.8, CH
**6′**			7.94, dd (1.2, 8.3)	129.1, CH	7.92, dd (1.4, 8.3)	129.1, CH
**7′**			-	165.2, C	-	165.0, C
**-OH**			12.29, s	-	12.04, s	-
**-OH’**			12.29, s	-	12.04, s	-

^a^ Signal too weak to be measured.

Compound **15**: white, amorphous solid; ${\left[\alpha \right]}_{D}^{25}$ −3.8 (*c* 0.1, MeOH); UV (MeOH) *λ*_max_ (log *ε*) 241 (3.33), 273 (3.19) nm; IR *ν*_max_ 2945, 1717, 1452, 1272, 1175, 1111, 1026 cm^-1^; ^1^H and ^13^C NMR, see Table 1; HRESIMS *m/z* 589.3543 ([M–H])^-^ (calcd for C_37_H_49_O_6_, 589.3524).

Compound **16**: white, amorphous solid; ${\left[\alpha \right]}_{D}^{25}$ −32.4 (*c* 0.1, MeOH); UV (MeOH) *λ*_max_ (log *ε*) 241 (3.35), 273 (3.15) nm; IR *ν*_max_ 2931, 1720, 1453, 1273, 1175, 1112, 1028 cm^-1^; ^1^H and ^13^C NMR, see Table 1; HRESIMS *m/z* 589.3541 ([M–H])^-^ (calcd for C_37_H_49_O_6_, 589.3524).

## Results and discussion

### Antiprotozoal activity of extracts

Based on the screening of 38 plants from Niger against *T. b. rhodesiense*, *T. cruzi*., *L. donovani* and *P. falciparum*, the MeOH extract from the leaves of *C. sieberiana*, the DCM extract from the root bark of *Z. mauritiana*, and the DCM extract from the roots of *S. alatum* were selected for further analysis (IC_50_ values <10 *μ*g/mL). These three extracts showed some cytotoxicity in L6 cells (Table 2).

**TABLE 2:** Antiparasitic activity and cytotoxicity of *Cassia sieberiana*, *Ziziphus mauritiana,* and *Sesamum alatum* extracts.

Plant	Part	Extract	IC_50_ ^a^ [µg/mL]				
			*T. b. rhodesiense*	*T. cruzi*	*L. donovani*	*P. falciparum*	Cytotoxicity^b^
*Cassia sieberiana*	Leaves	MeOH	5.7	8.3	>40	>20	18.5
*Ziziphus mauritiana*	Root bark	DCM	10.2	7.4	2.7	1.7	6.1
*Sesamum alatum*	Roots	DCM	12.5	42.0	5.9	7.6	13.4
Melarsoprol^c^			0.005				
Benznidazole^c^				0.667			
Miltefosine^c^					0.282		
Chloroquine^c^						0.003	
Podophyllotoxin^c^							0.008

^a^ The IC_50_ values are the means of two independent experiments (*n* = 2), except for *Cassia sieberiana,* where the experiments against *T. b. rhodesiense, L. donovani* and *P. falciparum* were performed three times (*n* = 3).

^b^ Rat skeletal myoblast (L6 cells).

^c^ Positive controls.

### Compound isolation and structure elucidation

**FIGURE 1:**
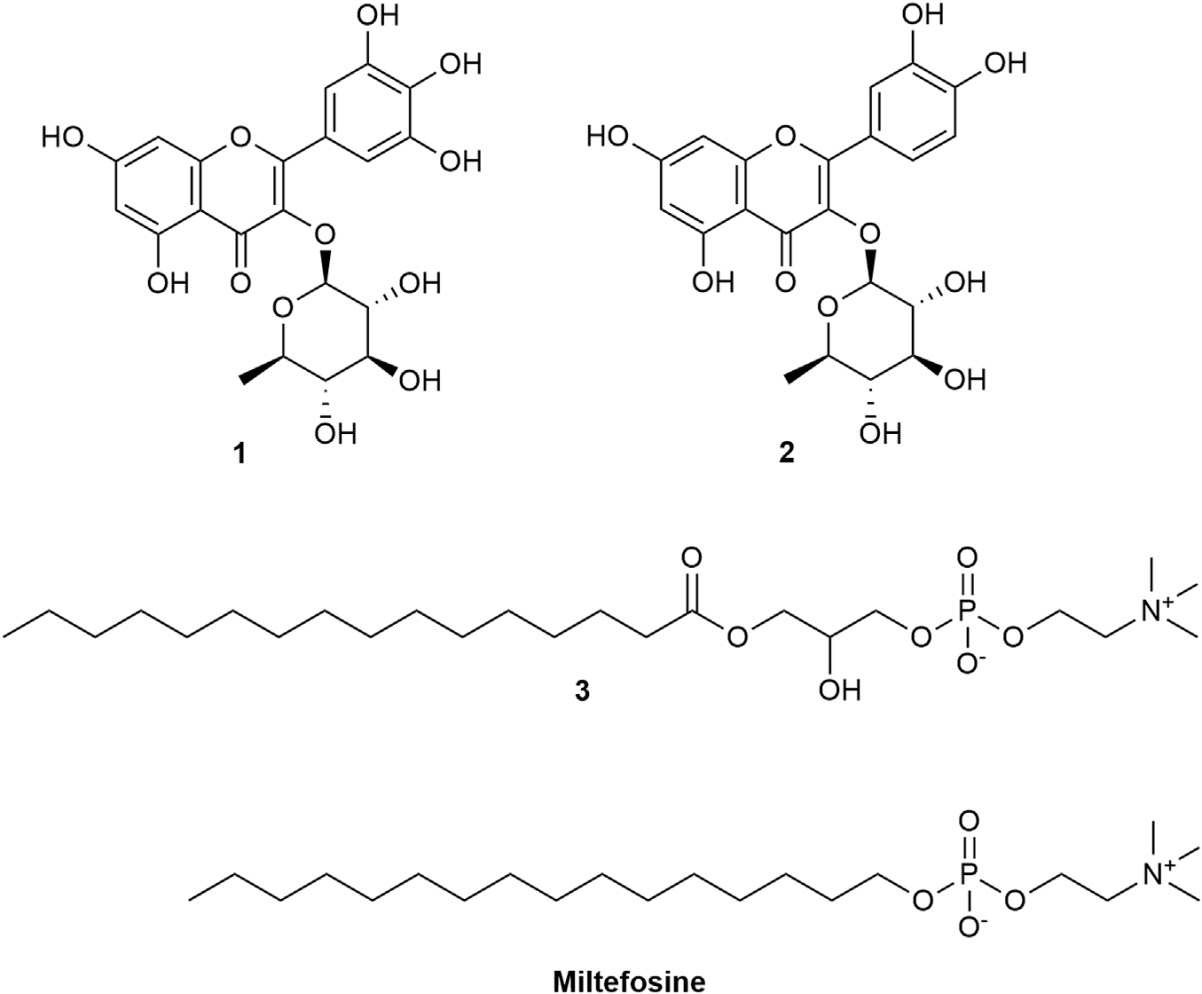
Structures of compounds **1** to **3** isolated from the MeOH leaf extract of *C. sieberiana*.

The LC-HRMS/MS-based dereplication of the MeOH leaf extract of *C. sieberiana* suggested the presence of several fatty acids, flavonoids, and chlorophyll derivatives. This extract was subjected to a flash column chromatography and semi-preparative HPLC to isolate two flavonoids and a phospholipid. The flavonoids were identified as myricitrin (**1**) (David et al., 1996) and quercitrin (**2**) (Mendez et al., 1995) **(** Supplementary Figures S1–S14 **)** by comparison of the experimental data with the literature. Compound **3** was defined to be 1-palmitoyl-lysolecithin based on HRESIMS and MS/MS spectra, as well as NMR data **(** Supplementary Figures S15-S21 **)**. This compound showed structural similarities with miltefosine (Figure 1), a compound initially developed as an anti-tumor drug, which was the first effective oral drug against visceral and cutaneous leishmaniasis (Croft et al., 2003; Dorlo et al., 2012). Compound **3** and miltefosine share a phosphocholine moiety. The former is known to be a cellular membrane component in eukaryotic cells (Kim et al., 2003). To the best of our knowledge, there is no biological activity reported for this compound but some analogues were isolated from the marine sponge *Spirastrella abata* and from deer antler, and showed antifungal activities (Min et al., 2001).

The DCM extract from the root bark of *Z. mauritiana* was subjected to flash column chromatography and semi-preparative HPLC to obtain eight cyclopeptide alkaloids and eight triterpenoids (**4—19**) (Figure 2). Their structures were elucidated by analysis of 1D and 2D NMR spectra, as well as HRESIMS data. Thirteen known compounds were identified as mauritine F (**4**) (Tschesche et al., 1974c), mauritine A (**5**) (Tschesche et al., 1972), ceanothic acid (**6**) (Julian et al., 1938), sanjoinine B (**7**) (Han et al., 1990), lotusanine A (**8**) (Zarga et al., 1995), nummularine K (**9**), (Tschesche et al., 1977), amphibine H (**10**) (Tschesche et al., 1974b), *N*-demethylamphibine H (**11**) (Tschesche et al., 1974a), mauritine M (**12**) (Panseeta et al., 2011), colubrinic acid (**14**) (Roitman and Jurd, 1978), ceanothenic acid (**17**) (Jou et al., 2004), 3-*O*-*trans-p*-coumaroylalphitolic acid (**18)** (Yagi et al., 1978) and betulinic acid (**19**) (Siddiqui et al., 1988) **(** Supplementary Figures S23–S79, Supplementary Figures S88–S94, and Supplementary Figures S113–132 **)**. To our knowledge, this is the first time that compounds **4**–**10**, **14**, and **17**–**19** are reported in the genus *Ziziphus*. In addition, compounds **13**, **15**, and **16** are described here for the first time.

**FIGURE 2:**
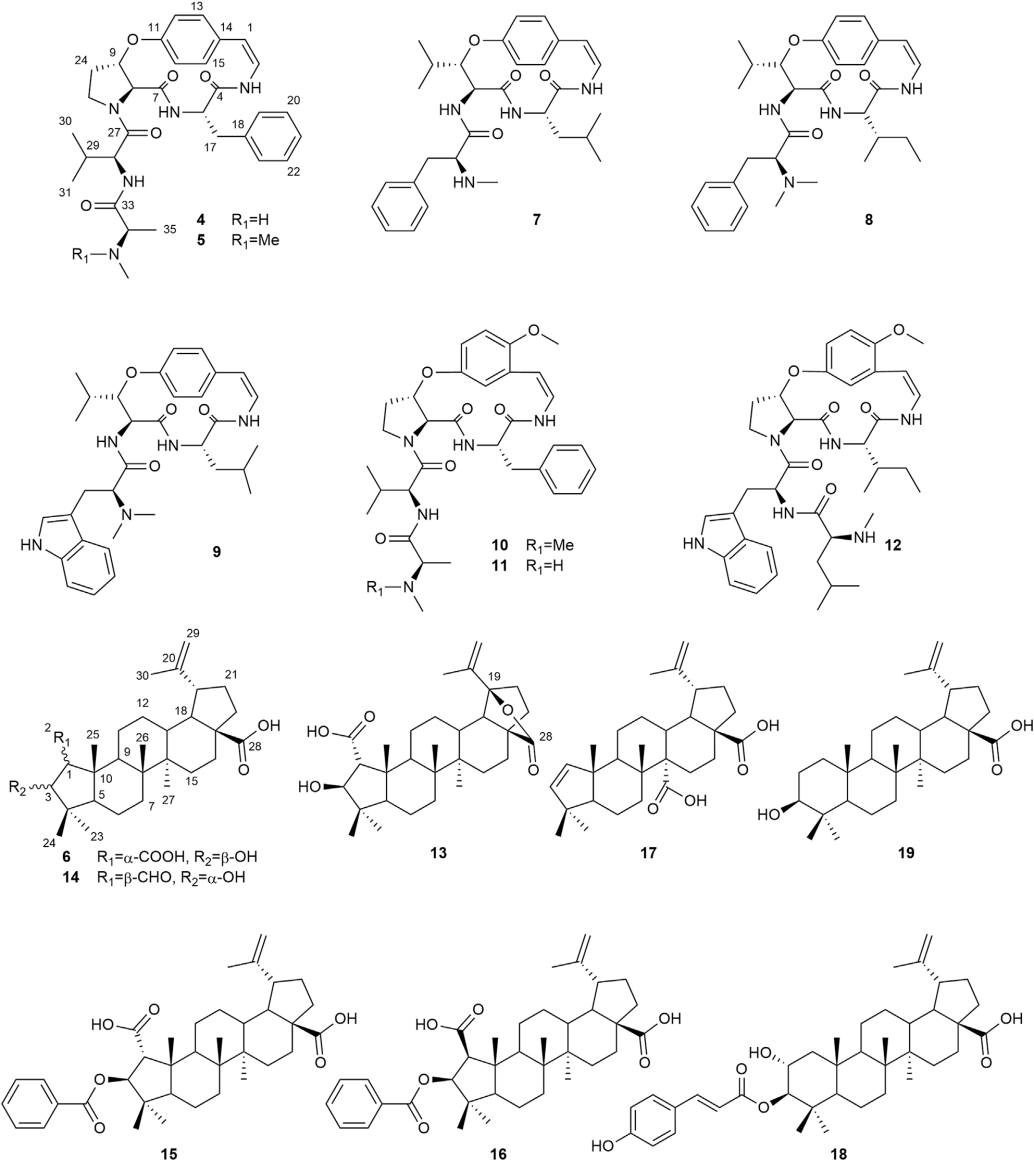
Structures of compounds **4** to **19** isolated from the DCM root bark extract of *Z. mauritiana*.

**FIGURE 3:**
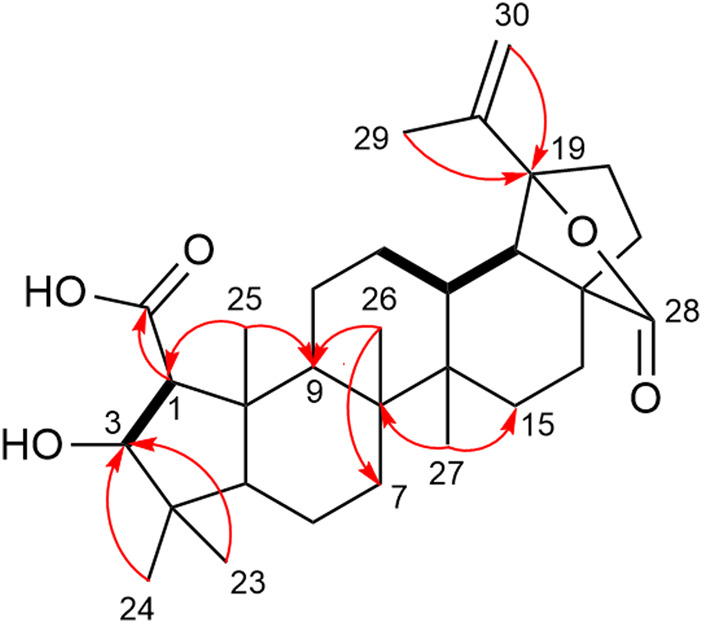
Key COSY (black bold line) and HMBC (red arrows) correlations of compound **13**.

**FIGURE 4:**
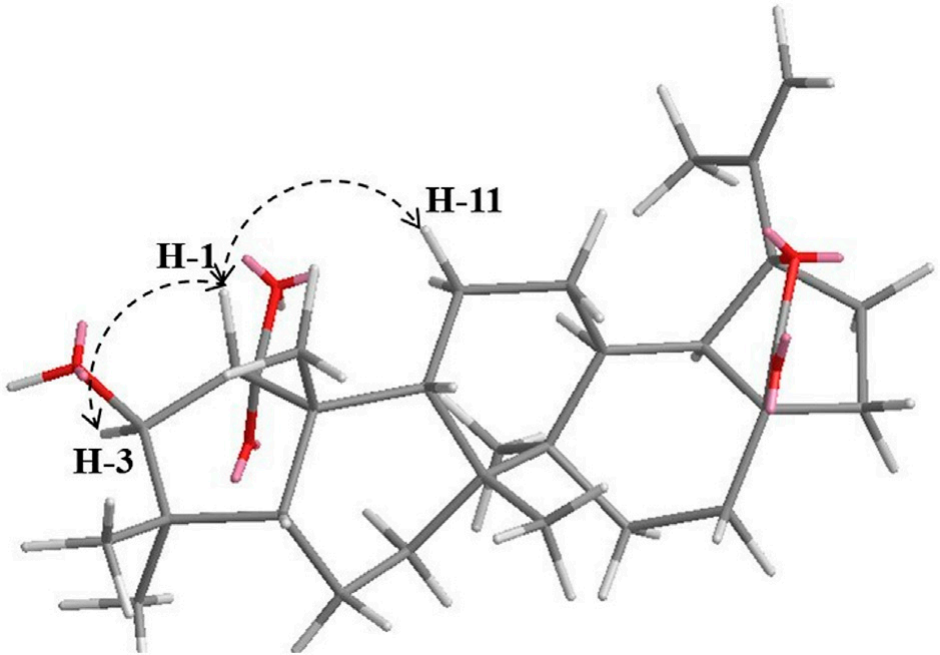
Key ROESY correlations of compound **13**.

Compound **13** was isolated as a white amorphous solid **(** Supplementary Figures S80–S87 **)**. The molecular formula C_30_H_44_O_5_ was determined by HRESIMS from the deprotonated ion peak at *m/z* 483.3123 ([M–H])^-^ (calcd for C_30_H_43_O_5_, 483.3105). The ^1^H and DEPTQ NMR data (Table 1) were similar to those of **6**, except for the hydrogen and carbon at position 19. The characteristic doublet of triplet proton signal corresponding to H-19 was absent in the ^1^H NMR spectrum of **13**. In the DEPTQ NMR spectrum, the signal of quaternary carbon resonance observed at *δ*_C_ 91.8 was attributed to C-19. The HMBC correlations of H-29 (*δ*_H_ 1.76) and H-30 (*δ*_H_ 4.93 and 5.13) to C-19 (*δ*_C_ 91.8) confirmed this attribution and indicated an E-ring-*γ* lactone (Figure 3). These chemical shifts were similar to those of its analogue 3-dehydroxyceanothan-27α-carboxy-28*β*-19*β*-olide (Kang et al., 2016). A ROESY experiment showed ROE correlations of H-3 (*δ*_H_ 3.95) and H-11 (*δ*_H_ 1.58) with H-1 (*δ*_H_ 2.23). This indicated an α-orientation of the carboxy group at C-1 (Figure 4). Compound **13** was identified as 3-dehydroxyceanothan-28*β*-19*β*-olide.

**FIGURE 5:**
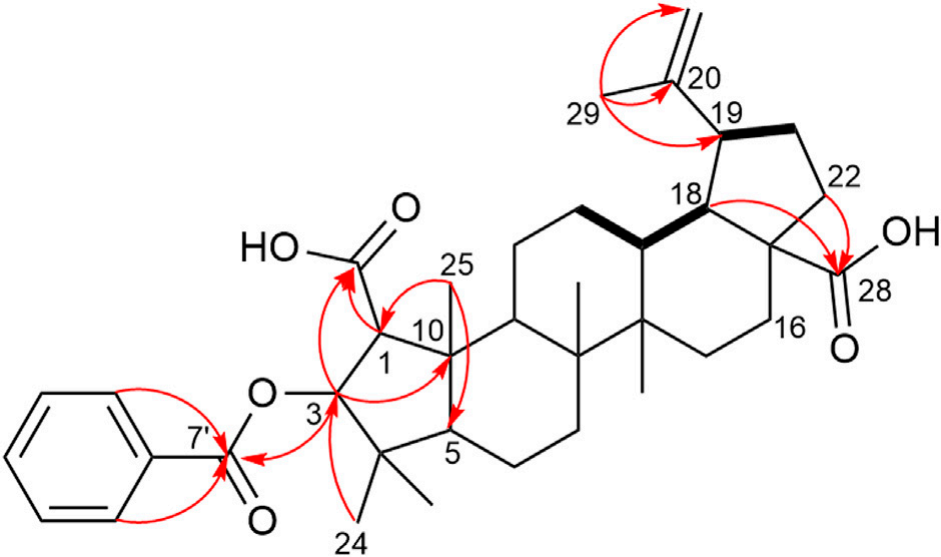
Key COSY (black bold line) and HMBC (red arrows) correlations of compounds **15** and **16**.

**FIGURE 6:**
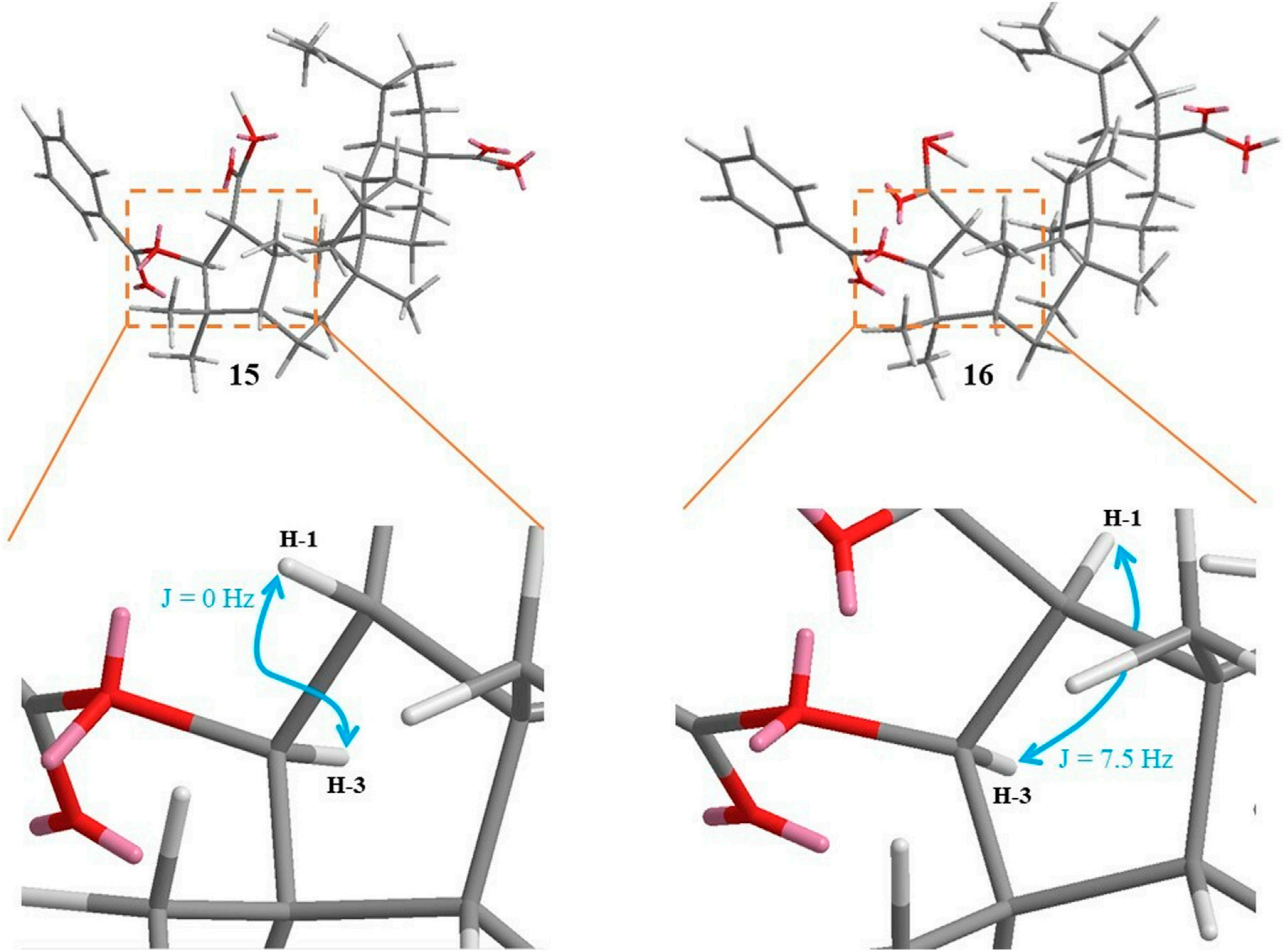
3D structures and ^3^ *J* coupling constants of compounds **15** and **16**.

**FIGURE 7:**
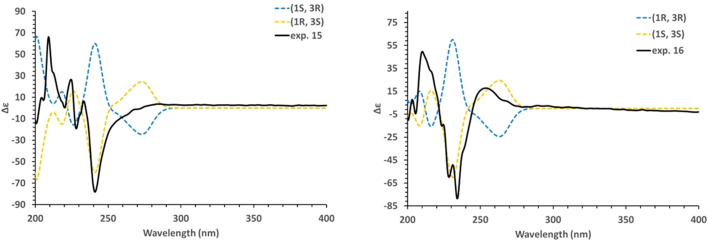
Experimental and B3LYP/def2svp//B3LYP/6-31G (d, p) calculated spectra in MeOH for compounds **15** and **16**.

Compound **15** was isolated as a white amorphous solid **(** Supplementary Figures S95–S103 **)**. The molecular formula C_37_H_50_O_6_ was determined by HRESIMS from the deprotonated ion peak at *m/z* 589.3543 ([M–H])^-^ (calcd for C_37_H_49_O_6_, 589.3524). The IR spectrum showed the presence of a conjugated carbonyl ester (1717 cm^-1^) and an aromatic ring (1452 cm^-1^). The ^1^H NMR spectrum of **15** (Table 1) indicated six methyl singlets (*δ*_H_ 1.65, 1.17, 1.07, 0.93, 0.91, 0.91) and two vinylic protons (*δ*_H_ 4.69, 4.58). The DEPTQ NMR spectrum (Table 1) exhibited 37 carbon resonances, which were attributed using the HSQC spectrum to six methyl groups, nine methylenes, twelve methines and ten non-protonated carbons. The ^1^H and DEPTQ NMR data showed great similarities with those of compound **6**. The ceanothic acid skeleton of **15** was confirmed by the HMBC correlations between H-25 (*δ*_H_ 1.07) and the methine carbons at *δ*_C_ 63.2 (C-1), 55.9 (C-5), and between H-1 (*δ*_H_ 2.54) and H-3 (*δ*_H_ 5.21) with the carboxylic acid carbon at *δ*_C_ 175.2 (C-2) (Figure 5). Another carboxylic acid group in position C-28 was attributed according to the HMBC correlations from H-18 (*δ*_H_ 1.52) and H-22 (*δ*_H_ 1.43 and 1.81) to C-28 (*δ*_C_ 177.2). The presence of an isopropenyl group at C-19 was supported by the HMBC correlations from H-29 (*δ*_H_ 1.65) to C-19, C-20 and C-30. The unique difference from compound **6** was the presence of an oxygenated methine singlet at H-3 (*δ*_H_ 5.21), with a down-field shift when compared to the signal at *δ*_H_ 3.92 of compound **6**. A benzoyl moiety linked to C-3 was revealed by the HMBC correlations from H-2' (*δ*_H_ 7.94), H-6' (*δ*_H_ 7.94) and H-3 (*δ*_H_ 5.21) to C-7' (*δ*_C_ 165.2). The HMBC correlations of H-23 (*δ*_H_ 1.17) and H-24 (*δ*_H_ 0.91) to C-3 (*δ*_C_ 85.3), and of H-3 (*δ*_H_ 5.21) to C-7' (*δ*_C_ 165.2) and C-10 (*δ*_C_ 48.6) confirmed the position of this group at C-3. The substitution at position C-3 is commonly observed in most triterpenoids because of their biosynthetic pathway originating from 2,3-oxidosqualene (Kang et al., 2016). The absence of coupling between H-1 and H-3 indicated an angle of 90° between the two protons and therefore a *trans* configuration (Figure 6). The absolute configuration of **15** was established by ECD data by comparison of experimental and calculated ECD spectra (Figure 7). A main negative Cotton Effect (CE) was observed at 241 nm perfectly matching the calculated curve for the enantiomer bearing the *β*-oriented benzoyl moiety. Compound **15** was identified as (1*R*, 3*S*)-3-*O*-benzoyl ceanothic acid.

Compound **16** was obtained as a white amorphous solid **(** Supplementary Figures S104–S112 **)**. The molecular formula C_37_H_50_O_6_ was determined from the deprotonated ion peak at *m/z* 589.3541 ([M–H])^-^ (calcd for C_37_H_49_O_6_, 589.3524) observed by HRESIMS. The presence of a hydroxy group, a conjugated carbonyl ester and an aromatic ring was confirmed by the IR spectrum from bands at 2931, 1720, 1453 cm^-1^, respectively. The ^1^H and ^13^C NMR data of **16** (Table 1) were closely related to those of **15**. The HMBC and COSY correlations suggested that **16** presented the same 2D structure as **15** (Figure 5). The coupling constant *J* = 7.5 Hz between H-1 (*δ*_H_ 2.67) and H-3 (*δ*_H_ 5.31) suggested a *cis* orientation and an angle close to 0° between H-1 and H-3 (Figure 6). The stereochemistry was established by ECD. A slight hypsochromic shift for the main CE band was observed relative to **15** (from 241 to 234 nm). This is possible due to the spatial interaction with the substituent at C-3. The experimental and calculated curves for the enantiomer 3*S* matched accordingly. These data demonstrated that **16** was different from **15** at the stereogenic center C-1 (Figure 7). Compound **16** was identified as (1*S*, 3*S*)-3-*O*-benzoyl ceanothic acid.

### Antiprotozoal activity

**FIGURE 8:**
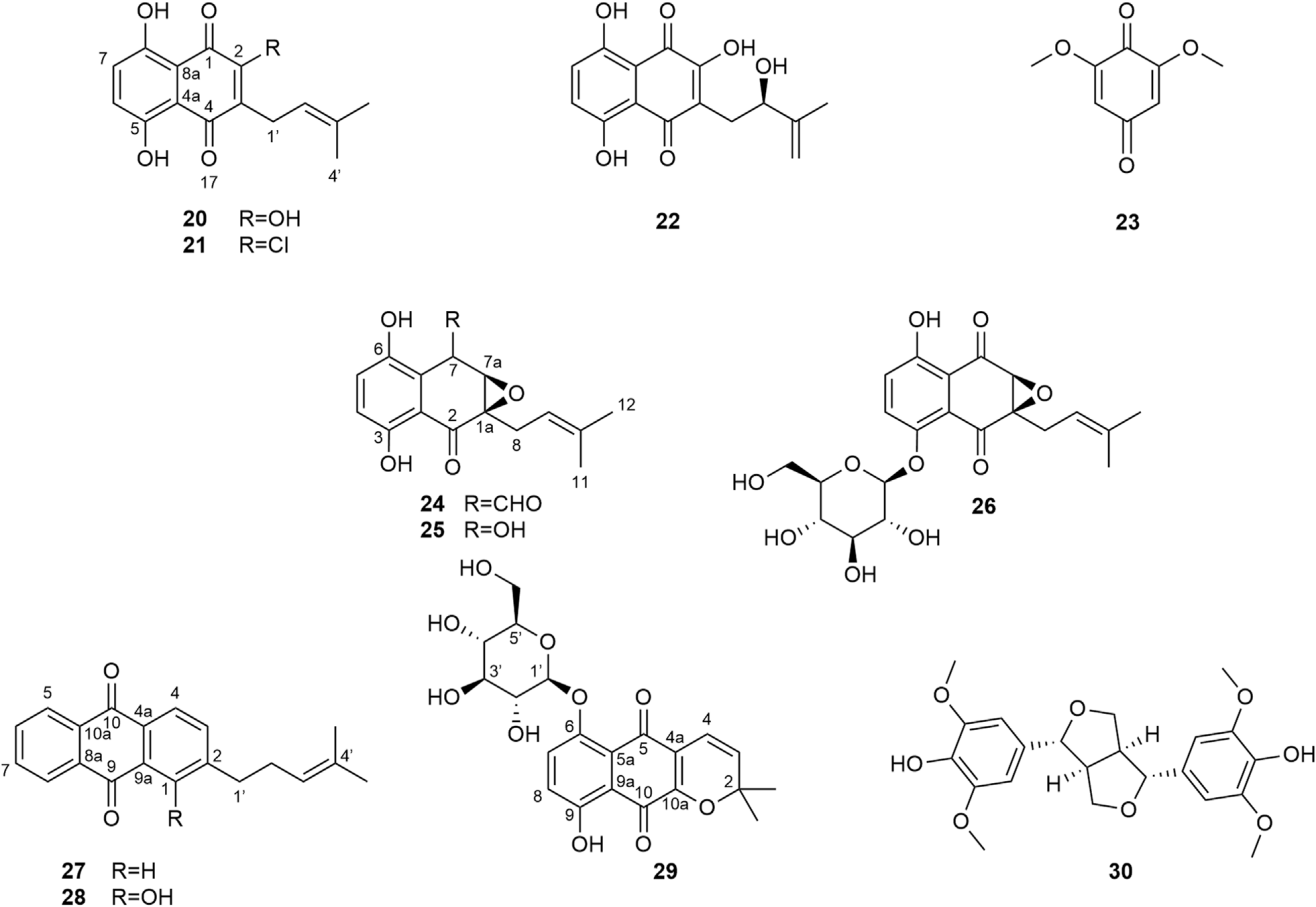
Structures of compounds **20** to **30** isolated from the DCM root extract of *S. alatum*.

The isolated compounds (**3**–**18**), as well as eleven compounds previously isolated from *S. alatum* by our group (**20**–**30**, Figure 8) (Saraux et al., 2022), were tested for their *in vitro* activity towards *T. b. rhodesiense*, *T. cruzi*, *L. donovani*, and *P. falciparum*, together with their cytotoxicity in the mouse skeletal L6 cells. Melarsoprol, benznidazole, miltefosine, chloroquine and podophyllotoxin were used as positive controls for the various activities. The biological data obtained are summarized in Table 3. Compounds **5**, **8**, **27**, and **28** were inactive (IC_50_ >50 µm).

**TABLE 3:** Antiparasitic activity and cytotoxicity of compounds **3**, **4**, **6**–**18,** **and** **20**–**26**, **29**, **30**.

	IC_50_ ^a^ [µM] (SI)							
Compound	*T. b. rhodesiense*			*T. cruzi*		*L. donovani*	*P. falciparum*	Cytotoxicity^b^
**3**	>20			12.1 ± 2.4 (1.7)^c^		>20	>10	>20
**4**	>50			>50		>50	24.3 ± 4.5 (>5.5)^c^	>50
**6**	>50			>50		29.0 ± 3.3 (>1.7)^c^	>50	>50
**7**	>50			>50		>50	23.1 ± 9.0 (>6.0)^c^	>50
**8**	>50			>50		>50	>50	>50
**9**	12.7 ± 3.8 (2.7)^c^			43.6 ± 7.1 (0.8)^c^		>50	20.7 ± 5.8 (1.6)^c^	33.6 ± 4.9
**10**	30.4 ± 4.6 (>2.5)^c^			>50		>50	15.1 ± 0.5 (>5.3)^c^	>50
**11**	>50			>50		44.8 ± 3.4 (>1.5)^c^	14.7 ± 4.2 (>5.4)^c^	>50
**12**	6.4 ± 2.0 (5.0)^c^			15.7 ± 1.1 (2.0)^c^		>50	2.6 ± 0.2 (12.2)^c^	31.8 ± 7.6
**13**	>50			>50		11.6 ± 2.4 (>9.3)^c^	29.1 ± 4.1 (>3.7)^c^	>50
**14**	>50			>50		1.9 ± 0.4 (>45)^c^	15.3 ± 4.1 (>5.5)^c^	>50
**15**	11.7 ± 1.0 (1.4)^c^			43.8 ± 8.4 (0.4)^c^		0.5 ± 0.2 (32.8)^c^	10.1 ± 0.6 (1.6)^c^	16.4 ± 5.8
**16**	30.6 ± 2.8 (1.1)^c^			>50		1.8 ± 0.7 (17.9)^c^	15.3 ± 1.5 (2.1)^c^	32.3 ± 5.1
**17**	>50			>50		2.6 ± 0.2 (>40)^c^	13.9 ± 4.0 (>8.0)^c^	>50
**18**	25.9 ± 2.6 (0.6)^c^			19.5 ± 0.6 (0.8)^c^		3.0 ± 0.2 (5.3)^c^	0.2 ± 0.04 (79.0)^c^	15.8 ± 6.4
**20**	6.9 ± 1.3 (2.0)^c^			27.4 ± 1.3 (0.5)^c^		1.9 ± 0.4 (7.4)^c^	45.0 ± 4.6 (0.3)^c^	14.0 ± 0.4
**21**	0.5 ± 0.3 (15.8)^c^			21.5 ± 0.2 (0.4)^c^		3.0 ± 0.4 (2.6)^c^	9.2 ± 2.1 (0.9)^c^	7.9 ± 1.3
**22**	36.0 ± 9.4 (0.8)^c^			>50		7.9 ± 0.9 (3.8)^c^	>50	30.4 ± 10.6
**23**	9.3 ± 3.1 (12.3)^c^			>50		15.1 ± 1.0 (7.6)^c^	36.9 ± 15.4 (3.1)^c^	>50
**24**	0.007 ± 0.003 (57.1)^c^			1.9 ± 0.9 (0.2)^c^		0.5 ± 0.2 (0.8)^c^	2.1 ± 0.3 (0.2)^c^	0.4 ± 0.16
**25**	2.5 ± 0.1 (7.6)^c^			>50		12.7 ± 5.3 (1.5)^c^	14.0 ± 1.9 (1.4)^c^	19.0 ± 2.3
**26**	9.9 ± 4.5 (1.2)^c^			39.2 ± 5.0 (0.3) ^c^		3.5 ± 0.5 (3.5)^c^	9.0 ± 5.4 (1.4)^c^	12.2 ± 1.8
**29**	12.5 ± 1.6 (3.2)^c^			>50		11.4 ± 2.3 (3.5)^c^	>50	40.3 ± 0.7
**30**	20.2 ± 1.8 (0.6)^c^			29.2 ± 11.4 (0.4)^c^		13.4 ± 2.3 (1.0)^c^	22.6 ± 2.0 (0.6)^c^	13.0 ± 1.7
**Melarsoprol**	0.016 ± 0.002							
**Benznidazole**				2.3 ± 0.2				
**Miltefosine**						0.52 ± 0.07		
**Chloroquine**							0.009 ± 0.001	
**Podophyllotoxin**								0.017 ± 0.002

^a^ The IC_50_ are the means of two independent assays ± deviation from the mean.

^b^ Rat skeletal myoblast (L6 cells).

^c^ Selectivity index (SI) = IC_50_ cytotoxicity/IC_50_ in the titled parasitic strain.

The antiparasitic activity of myricitrin (**1**) and quercitrin (**2**) was already described in the literature (Muzitano et al., 2006; Larit et al., 2021), and therefore it was not evaluated in the present study. Compound **3** was the only compound evaluated from the extract of *C. sieberiana* and was compared to the activity of miltefosine due to structural similarities. It exhibited a lower antileishmanial activity than miltefosine (Table 3), and the addition of a hydroxypropyl acetate in compound **3** seems to be responsible for this decrease. Among the cyclopeptide alkaloids isolated from the DCM extract of *Z. mauritiana* (**4**, **5**, **7**–**12**), a methoxy group at C-13 (**10**–**12**) seems to be favorable for the antiplasmodial activity. In addition, the indole group at C-29 present in compounds **9** and **12** seems to be important for the activity against *T. b. rhodesiense*. Overall, compound **12** was the most potent against *T. b. rhodesiense* and *P. falciparum*. This is the first report on the antileishmanial and antitrypanosomal activities of these compounds. The antiplasmodial activity of mauritine F (**4**), ceanothic acid (**6**), *N*-demethylamphibine H (**11**) and mauritine M (**12**) was already reported with IC_50_ values of 34.2, >20, 3.6 and 3.7 µM, respectively (Suksamrarn et al., 2006; Panseeta et al., 2011; Tuenter et al., 2017). This is in agreement with our study. Among the triterpene derivatives isolated from the DCM root bark extract of *Z. mauritiana* (**6**, **13**–**19**), betulinic acid (**19**), an already well-studied compound, was not tested in this work. Its antiparasitic activities with IC_50_ values of 4.2 µM against *T. b. rhodesiense* and 19 µM against *P. falciparum* was previously reported (Cretton et al., 2015). This compound did not present significant activity either against *Leishmania* spp., nor against *T. cruzi* with IC_50_ values >25 µM (Rocha et al., 2022). The triterpenoids tested in the present work (Table 3) were mainly active against *L. donovani* and presented a good selectivity index. The substitution with an epoxy group in compound **13** compared to compound **6** enhanced the activity toward *L. donovani* and *P. falciparum* but not against *T. cruzi*. The activity of compound **15**, which has a carboxylic acid group in *α* position at C-2, was slightly better than the one of compound **16**, which has the carboxylic acid group in *β* position, but there was no major improvement in its selectivity except against *L. donovani*. The high antileishmanial activity of **15** in comparison with compound **6** could possibly be due to the presence of a benzoic acid group at C-3. Compounds **15** and **16** were tested against the intracellular *L. donovani* amastigotes in mouse macrophages, but they were not active (IC_50_ >50 µM). This could be possibly because the molecules may not have reached the intracellular parasite or that they were metabolized by the host cell into a non-active metabolite. Compound **18** was the most active compound against *P. falciparum*. These results indicate that the antileishmanial and antiplasmodial activities shown by the DCM extract of *Z. mauritiana* was partly due to the triterpenoids.

The quinone derivatives previously isolated from the DCM root extract of *S. alatum* (**20**–**29**), were generally quite toxic towards L6 cells with selectivity indices <5. However, in some cases, there was a certain selectivity, indicating the importance of various substituents. The addition of a chlorine atom at position 2 of **21** compared to compound **20** led to the improvement of the activity against *T. b. rhodesiense.* The substitution of a hydroxyl at C-7 (compound **25**) for an aldehyde (compound **24**) led to a strong increase in activity against *T. b. rhodesiense*. The addition of an *O*-*β*-D-glucopyranoside substituent at C-3 in **26** and C-6 in **29** does not seem to be favorable for the activity. Compounds **24**, **25**, and **26** have a similar skeleton than vitamin K 2,3-epoxide, which is reduced to vitamin K by the vitamin K epoxide reductase (Robertson, 2004). This enzyme is also present in kinetoplastid parasites such as *T. b. rhodesiense, T. cruzi* and *L. major*. It is therefore possible that the quinone epoxides isolated (compounds **24**, **25**, and **26**) are substrates of this enzyme, which was shown to be involved in the anticoagulant activity of certain parasites. Compound **20** was previously evaluated for its antiplasmodial activity (IC_50_ value of 3.0 µM) (Kopa et al., 2014) and compound **29** for its antiparasitic activities (IC_50_ values of 12.6 and 7.6 µM against *L. amazonensis* and *T. cruzi*, respectively) (Costa et al., 2018). There are many reports on the antiparasitic activities and mechanisms of action of quinone derivatives. It was suggested that naphthoquinones could inhibit glycolysis (Prati et al., 2015), mitochondrial respiration (Salomão et al., 2013; Bombaça et al., 2018; Mendonca et al., 2018) and topoisomerase IB (Perez-Pertejo et al., 2019) in parasites. In addition, quinone derivatives are known to be inhibitors of cytochrome b in *P. falciparum* (Lane et al., 2018).

## Conclusion

This study led to the isolation of three compounds that have not been previously reported in the literature, and sixteen known compounds from two Nigerien plant extracts, the MeOH extract from the leaves of *C. sieberiana* and the DCM extract from the root bark of *Z. mauritiana*. Fifteen of those compounds, as well as eleven compounds previously isolated from *S. alatum*, were evaluated for their antiprotozoal activities. Among these, compound **24** showed the strongest activity against *T. b. rhodesiense*. Moreover, compounds **15** and **18** exhibited activities in the low micromolar range against *L. donovani* and *P. falciparum*, respectively.

## Data Availability

The original contributions presented in the study are included in the article/Supplementary Material. Further inquiries can be directed to the corresponding author.
