# Supplementary material for: Antiprotozoal activity of natural products from Nigerien plants used in folk medicine
Source: Front Pharmacol. 2023 Jun 23;14:1190241. doi: 10.3389/fphar.2023.1190241 (PMC10326435; doi:10.3389/fphar.2023.1190241)
Supplement: Supplementary file 1 [file DataSheet1.docx]

Supplementary Material

**Antiprotozoal activity of natural products from Niger plants used in folk**

**Ozlem Sevik Kilicaslan^1,2†^, Sylvian Cretton^1,2^, Estelle Hausmann^1,2^, Luis Quirós-Guerrero^1,2^, Soumana Karimou^3^, Marcel Kaiser^4,5^, Pascal Mäser^4,5^, Philippe Christen^1,2^ and Muriel Cuendet****^1,2^***

*** Correspondence:**Corresponding Author
[Muriel.cuendet@unige.ch](mailto:Muriel.cuendet@unige.ch)

[**Figure S1.** ESIMS spectrum of **1** 6](#_Toc135086830)

[**Figure S2.** ^1^H NMR (600 MHz, CD_3_OD) spectrum of **1** 6](#_Toc135086831)

[**Figure S3.** DEPTQ NMR (150 MHz, CD_3_OD) spectrum of **1** 7](#_Toc135086832)

[**Figure S4**. COSY (600 MHz, CD_3_OD) spectrum of **1** 7](#_Toc135086833)

[**Figure S5.** HSQC (600 MHz, CD_3_OD) spectrum of **1** 8](#_Toc135086834)

[**Figure S6**. HMBC (600 MHz, CD_3_OD) spectrum of **1** 8](#_Toc135086835)

[**Figure S7.** ROESY (600 MHz, CD_3_OD) spectrum of **1** 9](#_Toc135086836)

[**Figure S8.** ESIMS spectrum of **2** 9](#_Toc135086837)

[**Figure S9.** ^1^H NMR (600 MHz, CD_3_OD) spectrum of **2** 10](#_Toc135086838)

[**Figure S10.** DEPTQ NMR (150 MHz, CD_3_OD) spectrum of **2** 10](#_Toc135086839)

[**Figure S11.** COSY (600 MHz, CD_3_OD) spectrum of **2** 11](#_Toc135086840)

[**Figure S12.** HSQC (600 MHz, CD_3_OD) spectrum of **2** 11](#_Toc135086841)

[**Figure S13.** HMBC (600 MHz, CD_3_OD) spectrum of **2** 12](#_Toc135086842)

[**Figure S14.** ROESY (600 MHz, CD_3_OD) spectrum of **2** 12](#_Toc135086843)

[**Figure S15.** ESIMS spectrum of **3** 13](#_Toc135086844)

[**Figure S16.** ^1^H NMR (600 MHz, CD_3_OD) spectrum of **3** 13](#_Toc135086845)

[**Figure S17.** DEPTQ NMR (150 MHz, CD_3_OD) spectrum of **3** 14](#_Toc135086846)

[**Figure S18.** COSY (600 MHz, CD_3_OD) spectrum of **3** 14](#_Toc135086847)

[**Figure S19.** HSQC (600 MHz, CD_3_OD) spectrum of **3** 15](#_Toc135086848)

[**Figure S20.** HMBC (600 MHz, CD_3_OD) spectrum of **3** 15](#_Toc135086849)

[**Figure S21.** ^31^P (600 MHz, CD_3_OD) spectrum of **3** 16](#_Toc135086850)

[**Figure S22.** ESIMS spectrum of **4** 16](#_Toc135086851)

[**Figure S23.** ^1^H NMR (600 MHz, DMSO-*d_6_*) spectrum of **4** 17](#_Toc135086852)

[**Figure S24.** DEPTQ NMR (150 MHz, DMSO-*d_6_*) spectrum of **4** 17](#_Toc135086853)

[**Figure S25.** COSY (600 MHz, DMSO-*d_6_*) spectrum of **4** 18](#_Toc135086854)

[**Figure S26.** HSQC (600 MHz, DMSO-*d_6_*) spectrum of **4** 18](#_Toc135086855)

[**Figure S27.** HMBC (600 MHz, DMSO-*d_6_*) spectrum of **4** 19](#_Toc135086856)

[**Figure S28.** ROESY (600 MHz, DMSO-*d_6_*) spectrum of **4** 19](#_Toc135086857)

[**Figure S29.** ESIMS spectrum of **5** 20](#_Toc135086858)

[**Figure S30.** ^1^H NMR (600 MHz, DMSO-*d_6_*) spectrum of **5** 20](#_Toc135086859)

[**Figure S31.** DEPTQ NMR (150 MHz, DMSO-*d_6_*) spectrum of **5** 21](#_Toc135086860)

[**Figure S32.** COSY (600 MHz, DMSO-*d_6_*) spectrum of **5** 21](#_Toc135086861)

[**Figure S33.** HSQC (600 MHz, DMSO-*d_6_*) spectrum of **5** 22](#_Toc135086862)

[**Figure S34.** HMBC (600 MHz, DMSO-*d_6_*) spectrum of **5** 22](#_Toc135086863)

[**Figure S35.** ROESY (600 MHz, DMSO-*d_6_*) spectrum of **5** 23](#_Toc135086864)

[**Figure S36.** ESIMS spectrum of **6** 23](#_Toc135086865)

[**Figure S37.** ^1^H NMR (600 MHz, DMSO-*d_6_*) spectrum of **6** 24](#_Toc135086866)

[**Figure S38.** DEPTQ NMR (150 MHz, DMSO-*d_6_*) spectrum of **6** 24](#_Toc135086867)

[**Figure S39.** COSY (600 MHz, DMSO-*d_6_*) spectrum of **6** 25](#_Toc135086868)

[**Figure S40.** HSQC (600 MHz, DMSO-*d_6_*) spectrum of **6** 25](#_Toc135086869)

[**Figure S41.** HMBC (600 MHz, DMSO-*d_6_*) spectrum of **6** 26](#_Toc135086870)

[**Figure S42.** ROESY (600 MHz, DMSO-*d_6_*) spectrum of **6** 26](#_Toc135086871)

[**Figure S43.** ESIMS spectrum of **7** 27](#_Toc135086872)

[**Figure S44.** ^1^H NMR (600 MHz, DMSO-*d_6_*) spectrum of **7** 27](#_Toc135086873)

[**Figure S45.** DEPTQ NMR (150 MHz, DMSO-*d_6_*) spectrum of **7** 28](#_Toc135086874)

[**Figure S46.** COSY (600 MHz, DMSO-*d_6_*) spectrum of **7** 28](#_Toc135086875)

[**Figure S 47.** HSQC (600 MHz, DMSO-*d_6_*) spectrum of **7** 29](#_Toc135086876)

[**Figure S48.** HMBC (600 MHz, DMSO-*d_6_*) spectrum of **7** 29](#_Toc135086877)

[**Figure S49.** ROESY (600 MHz, DMSO-*d_6_*) spectrum of **7** 30](#_Toc135086878)

[**Figure S50.** ESIMS spectrum of **8** 30](#_Toc135086879)

[**Figure S51.** ^1^H NMR (600 MHz, DMSO-*d_6_*) spectrum of **8** 31](#_Toc135086880)

[**Figure S52.** DEPTQ NMR (150 MHz, DMSO-*d_6_*) spectrum of **8** 31](#_Toc135086881)

[**Figure S53.** COSY (600 MHz, DMSO-*d_6_*) spectrum of **8** 32](#_Toc135086882)

[**Figure S54.** HSQC (600 MHz, DMSO-*d_6_*) spectrum of **8** 32](#_Toc135086883)

[**Figure S55.** HMBC (600 MHz, DMSO-*d_6_*) spectrum of **8** 33](#_Toc135086884)

[**Figure S56.** ROESY (600 MHz, DMSO-*d_6_*) spectrum of **8** 33](#_Toc135086885)

[**Figure S57.** ^1^H NMR (600 MHz, DMSO-*d_6_*) spectrum of **9** 34](#_Toc135086886)

[**Figure S58.** COSY (600 MHz, DMSO-*d_6_*) spectrum of **9** 34](#_Toc135086887)

[**Figure S59.** HSQC (600 MHz, DMSO-*d_6_*) spectrum of **9** 35](#_Toc135086888)

[**Figure S60.** HMBC (600 MHz, DMSO-*d_6_*) spectrum of **9** 35](#_Toc135086889)

[**Figure S61.** ROESY (600 MHz, DMSO-*d_6_*) spectrum of **9** 36](#_Toc135086890)

[**Figure S62.** ^1^H NMR (600 MHz, DMSO-*d_6_*) spectrum of **10** 36](#_Toc135086891)

[**Figure S63.** DEPTQ NMR (150 MHz, DMSO-*d_6_*) spectrum of **10** 37](#_Toc135086892)

[**Figure S64.** COSY (600 MHz, DMSO-*d_6_*) spectrum of **10** 37](#_Toc135086893)

[**Figure S65.** HSQC (600 MHz, DMSO-*d_6_*) spectrum of **10** 38](#_Toc135086894)

[**Figure S66.** HMBC (600 MHz, DMSO-*d_6_*) spectrum of **10** 38](#_Toc135086895)

[**Figure S67.** ROESY (600 MHz, DMSO-*d_6_*) spectrum of **10** 39](#_Toc135086896)

[**Figure S68.** ^1^H NMR (600 MHz, DMSO-*d_6_*) spectrum of **11** 39](#_Toc135086897)

[**Figure S69.** COSY (600 MHz, DMSO-*d_6_*) spectrum of **11** 40](#_Toc135086898)

[**Figure S70.** HSQC (600 MHz, DMSO-*d_6_*) spectrum of **11** 40](#_Toc135086899)

[**Figure S71.** HMBC (600 MHz, DMSO-*d_6_*) spectrum of **11** 41](#_Toc135086900)

[**Figure S72.** ROESY (600 MHz, DMSO-*d_6_*) spectrum of **11** 41](#_Toc135086901)

[**Figure S73.** ESIMS spectrum of **12** 42](#_Toc135086902)

[**Figure S74.** ^1^H NMR (600 MHz, DMSO-*d_6_*) spectrum of **12** 42](#_Toc135086903)

[**Figure S75.** DEPTQ NMR (150 MHz, DMSO-*d_6_*) spectrum of **12** 43](#_Toc135086904)

[**Figure S76.** COSY (600 MHz, DMSO-*d_6_*) spectrum of **12** 43](#_Toc135086905)

[**Figure S77.** HSQC (600 MHz, DMSO-*d_6_*) spectrum of **12** 44](#_Toc135086906)

[**Figure S78.** HMBC (600 MHz, DMSO-*d_6_*) spectrum of **12** 44](#_Toc135086907)

[**Figure S79.** ROESY (600 MHz, DMSO-*d_6_*) spectrum of **12** 45](#_Toc135086908)

[**Figure S80.** UV spectrum of **13** 45](#_Toc135086909)

[**Figure S81.** HRESIMS spectrum of **13** 46](#_Toc135086910)

[**Figure S82.** ^1^H NMR (600 MHz, DMSO-*d_6_*) spectrum of **13** 46](#_Toc135086911)

[**Figure S83.** DEPTQ NMR (150 MHz, DMSO-*d_6_*) spectrum of **13** 47](#_Toc135086912)

[**Figure S84.** COSY (600 MHz, DMSO-*d_6_*) spectrum of **13** 47](#_Toc135086913)

[**Figure S85.** HSQC (600 MHz, DMSO-*d_6_*) spectrum of **13** 48](#_Toc135086914)

[**Figure S86.** HMBC (600 MHz, DMSO-*d_6_*) spectrum of **13** 48](#_Toc135086915)

[**Figure S87.** ROESY (600 MHz, DMSO-*d_6_*) spectrum of **13** 49](#_Toc135086916)

[**Figure S88.** ESIMS spectrum of **14** 49](#_Toc135086917)

[**Figure S89.** ^1^H NMR (600 MHz, DMSO-*d_6_*) spectrum of **14** 50](#_Toc135086918)

[**Figure S90.** DEPTQ NMR (150 MHz, DMSO-*d_6_*) spectrum of **14** 50](#_Toc135086919)

[**Figure S91.** COSY (600 MHz, DMSO-*d_6_*) spectrum of **14** 51](#_Toc135086920)

[**Figure S92.** HSQC (600 MHz, DMSO-*d_6_*) spectrum of **14** 51](#_Toc135086921)

[**Figure S93.** HMBC (600 MHz, DMSO-*d_6_*) spectrum of **14** 52](#_Toc135086922)

[**Figure S94.** ROESY (600 MHz, DMSO-*d_6_*) spectrum of **14** 52](#_Toc135086923)

[**Figure S95.** IR spectrum of **15** 53](#_Toc135086924)

[**Figure S96.** UV spectrum of **15** 53](#_Toc135086925)

[**Figure S97.** HRESIMS spectrum of **15** 53](#_Toc135086926)

[**Figure S98.** ^1^H NMR (600 MHz, DMSO-*d_6_*) spectrum of **15** 54](#_Toc135086927)

[**Figure S99.** DEPTQ NMR (150 MHz, DMSO-*d_6_*) spectrum of **15** 54](#_Toc135086928)

[**Figure S100.** COSY (600 MHz, DMSO-*d_6_*) spectrum of **15** 55](#_Toc135086929)

[**Figure S101.** HSQC (600 MHz, DMSO-*d_6_*) spectrum of **15** 55](#_Toc135086930)

[**Figure S102.** HMBC (600 MHz, DMSO-*d_6_*) spectrum of **15** 56](#_Toc135086931)

[**Figure S103.** ROESY (600 MHz, DMSO-*d_6_*) spectrum of **15** 56](#_Toc135086932)

[**Figure S104.** IR spectrum of **16** 57](#_Toc135086933)

[**Figure S105.** UV spectrum of **16** 57](#_Toc135086934)

[**Figure S106.** HRESIMS spectrum of **16** 58](#_Toc135086935)

[**Figure S107.** ^1^H NMR (600 MHz, DMSO-*d_6_*) spectrum of **16** 58](#_Toc135086936)

[**Figure S108.** DEPTQ NMR (150 MHz, DMSO-*d_6_*) spectrum of **16** 59](#_Toc135086937)

[**Figure S109.** COSY (600 MHz, DMSO-*d_6_*) spectrum of **16** 59](#_Toc135086938)

[**Figure S110.** HSQC (600 MHz, DMSO-*d_6_*) spectrum of **16** 60](#_Toc135086939)

[**Figure S111.** HMBC (600 MHz, DMSO-*d_6_*) spectrum of **16** 60](#_Toc135086940)

[**Figure S112.** ROESY (600 MHz, DMSO-*d_6_*) spectrum of **16** 61](#_Toc135086941)

[**Figure S113.** ESIMS spectrum of **17** 61](#_Toc135086942)

[**Figure S114.** ^1^H NMR (600 MHz, DMSO-*d_6_*) spectrum of **17** 62](#_Toc135086943)

[**Figure S115.** COSY (600 MHz, DMSO-*d_6_*) spectrum of **17** 62](#_Toc135086944)

[**Figure S116.** HSQC (600 MHz, DMSO-*d_6_*) spectrum of **17** 63](#_Toc135086945)

[**Figure S117.** HMBC (600 MHz, DMSO-*d_6_*) spectrum of **17** 64](#_Toc135086946)

[**Figure S118.** ROESY (600 MHz, DMSO-*d_6_*) spectrum of **17** 64](#_Toc135086947)

[**Figure S119.** ESIMS spectrum of **18** 64](#_Toc135086948)

[**Figure S120.** ^1^H NMR (600 MHz, DMSO-*d_6_*) spectrum of **18** 65](#_Toc135086949)

[**Figure S121.** DEPTQ NMR (150 MHz, DMSO-*d_6_*) spectrum of **18** 66](#_Toc135086950)

[**Figure S122.** COSY (600 MHz, DMSO-*d_6_*) spectrum of **18** 66](#_Toc135086951)

[**Figure S123.** HSQC (600 MHz, DMSO-*d_6_*) spectrum of **18** 67](#_Toc135086952)

[**Figure S124.** HMBC (600 MHz, DMSO-*d_6_*) spectrum of **18** 67](#_Toc135086953)

[**Figure S125.** ROESY (600 MHz, DMSO-*d_6_*) spectrum of **18** 68](#_Toc135086954)

[**Figure S126.** ESIMS spectrum of **19** 68](#_Toc135086955)

[**Figure S127.** ^1^H NMR (600 MHz, DMSO-*d_6_*) spectrum of **19** 69](#_Toc135086956)

[**Figure S128.** DEPTQ NMR (150 MHz, DMSO-*d_6_*) spectrum of **19** 69](#_Toc135086957)

[**Figure S129.** COSY (600 MHz, DMSO-*d_6_*) spectrum of **19** 70](#_Toc135086958)

[**Figure S130.** HSQC (600 MHz, DMSO-*d_6_*) spectrum of **19** 70](#_Toc135086959)

[**Figure S131.** HMBC (600 MHz, DMSO-*d_6_*) spectrum of **19** 71](#_Toc135086960)

[**Figure S132.** ROESY (600 MHz, DMSO-*d_6_*) spectrum of **19** 71](#_Toc135086961)


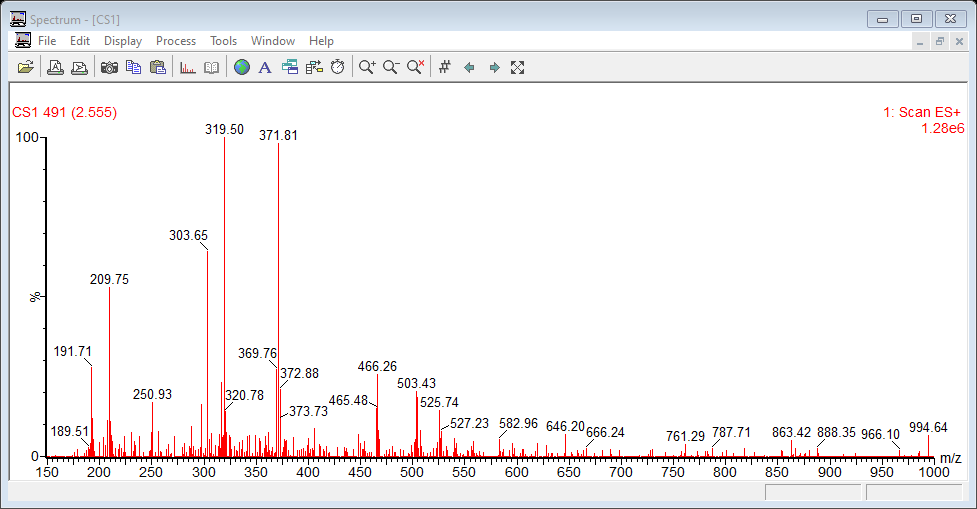


Figure S1. ESIMS spectrum of 1


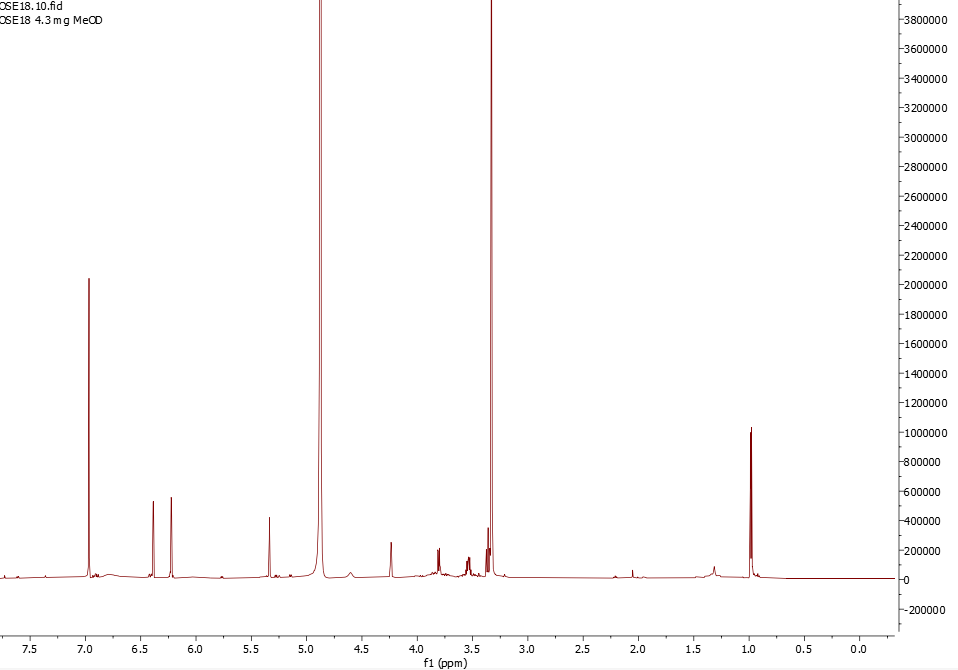


Figure S2. ^1^H NMR (600 MHz, CD_3_OD) spectrum of 1


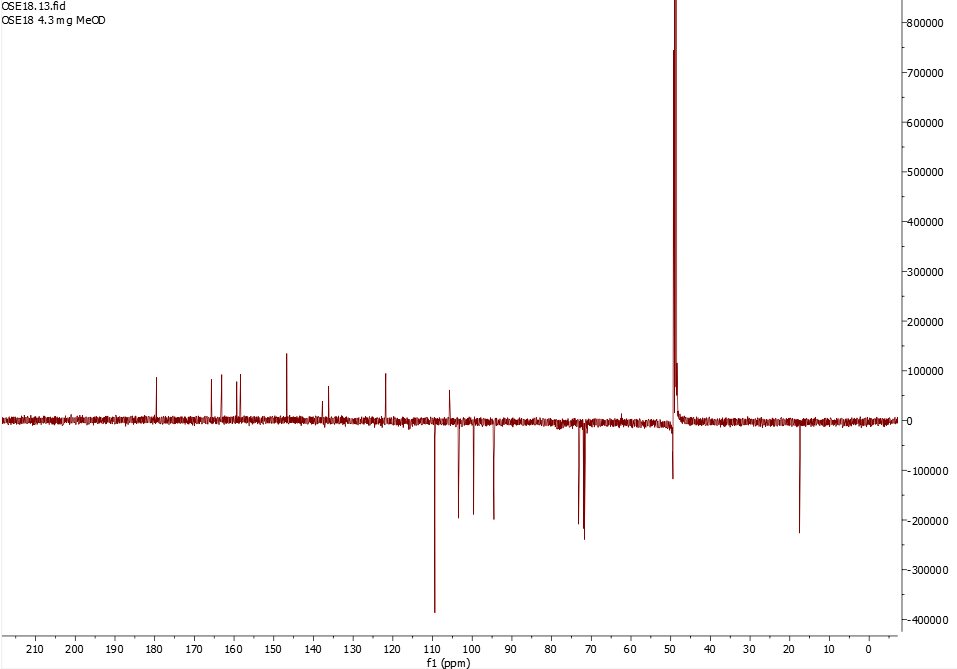


Figure S3. DEPTQ NMR (150 MHz, CD_3_OD) spectrum of 1


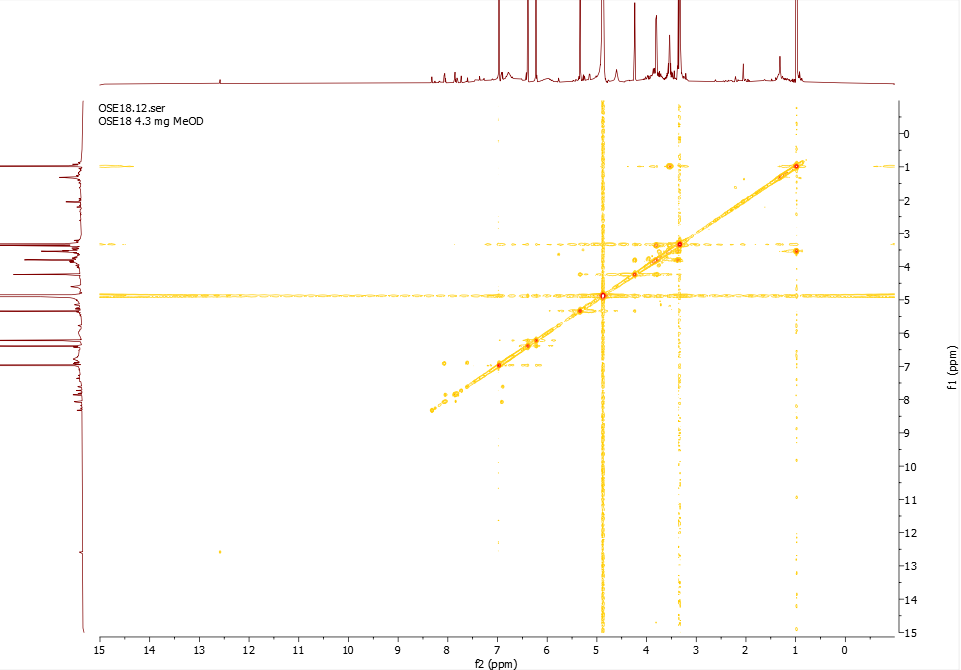


**Figure S4**. COSY (600 MHz, CD_3_OD) spectrum of **1**


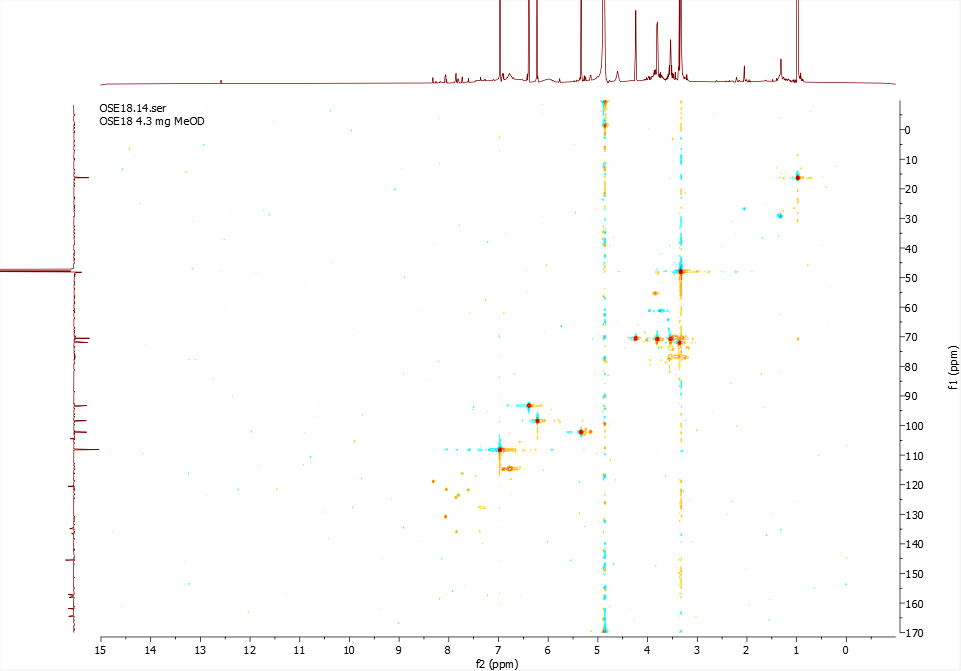


Figure S5. HSQC (600 MHz, CD_3_OD) spectrum of 1


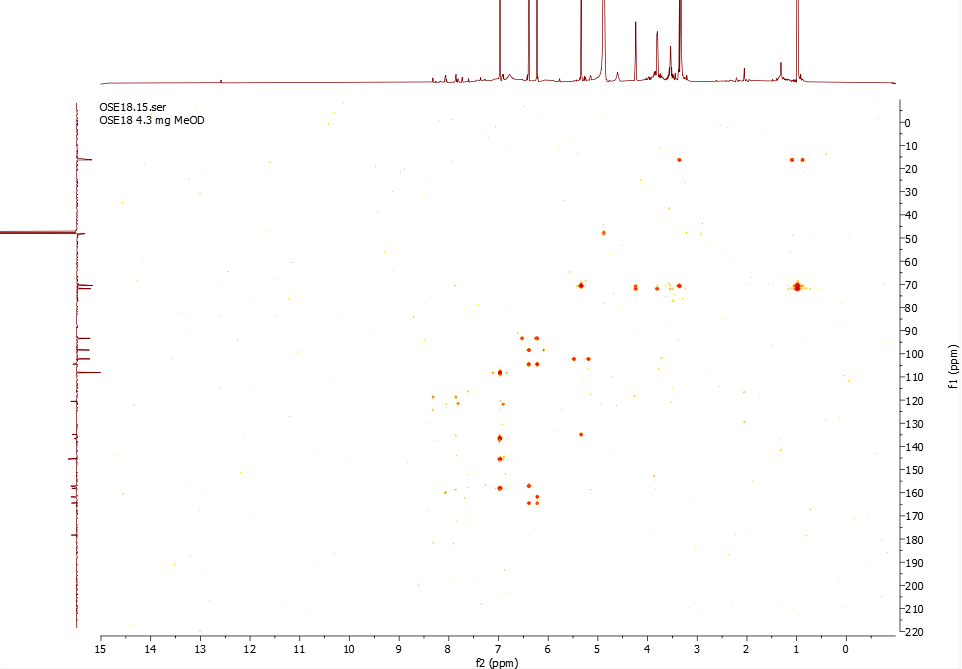


**Figure S6**. HMBC (600 MHz, CD_3_OD) spectrum of **1**


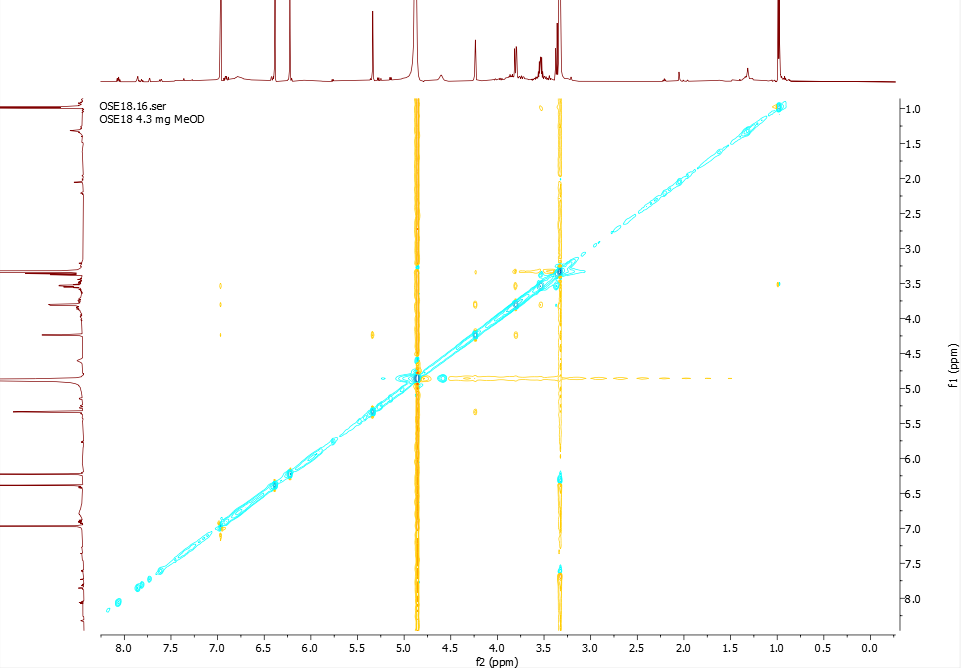


Figure S7. ROESY (600 MHz, CD_3_OD) spectrum of 1


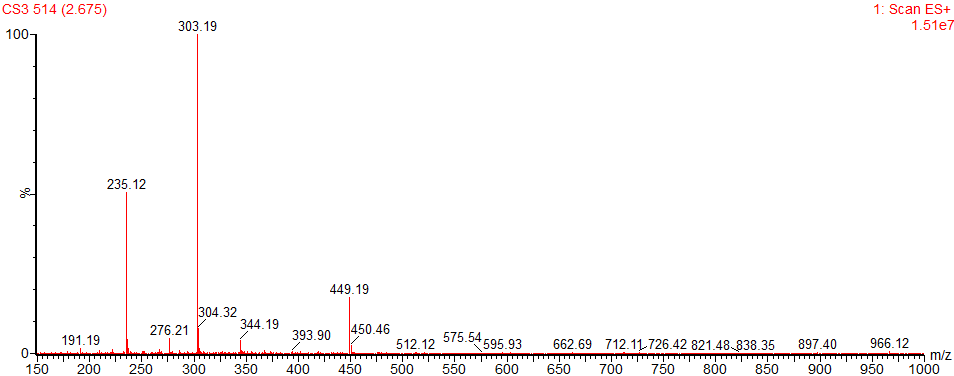


Figure S8. ESIMS spectrum of 2


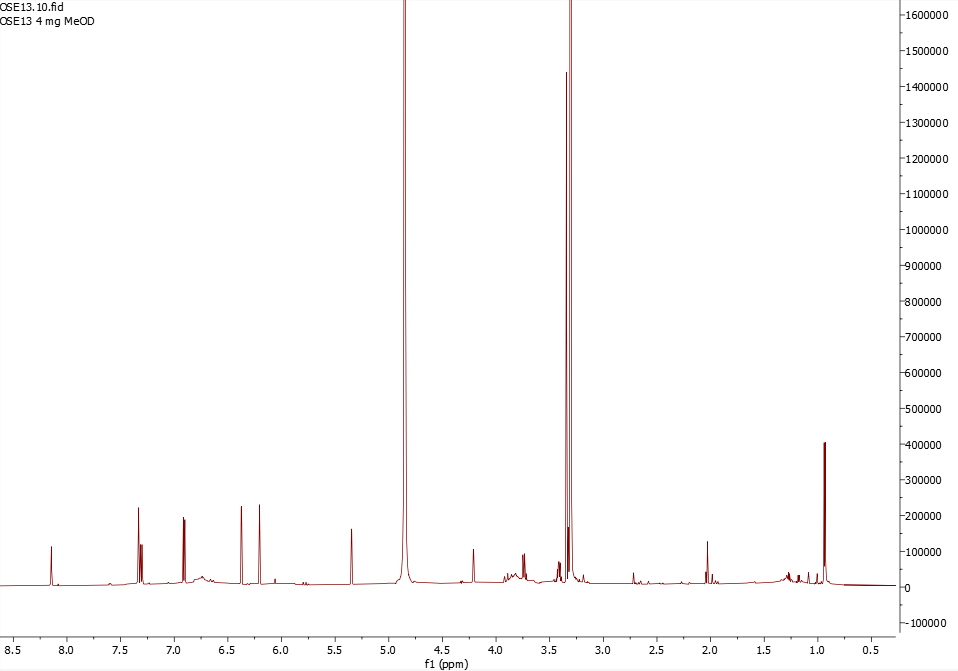


**Figure S9.** ^1^H NMR (600 MHz, CD_3_OD) spectrum of **2**


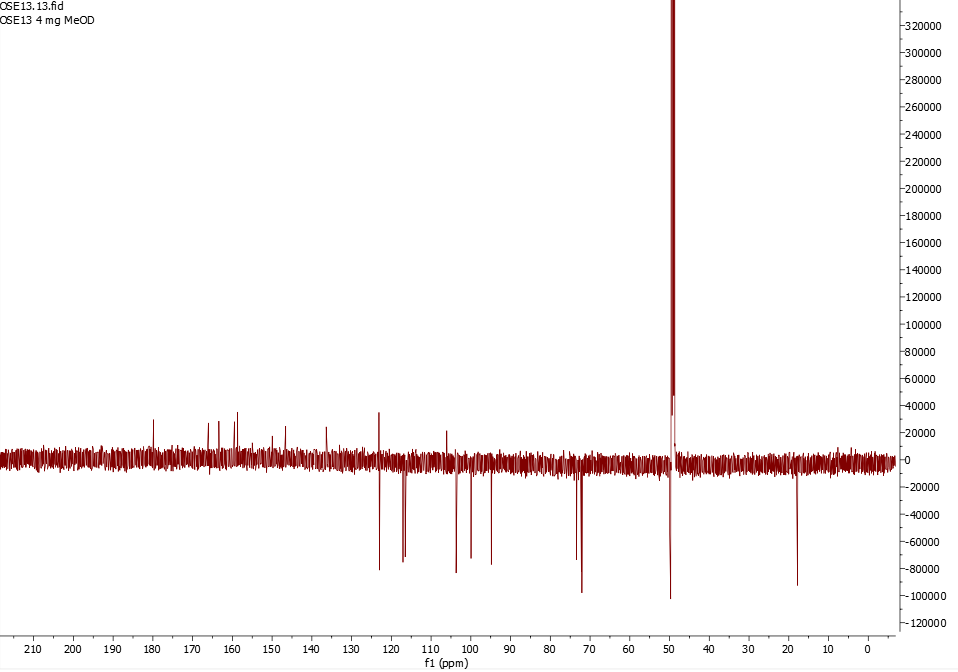


Figure S10. DEPTQ NMR (150 MHz, CD_3_OD) spectrum of 2


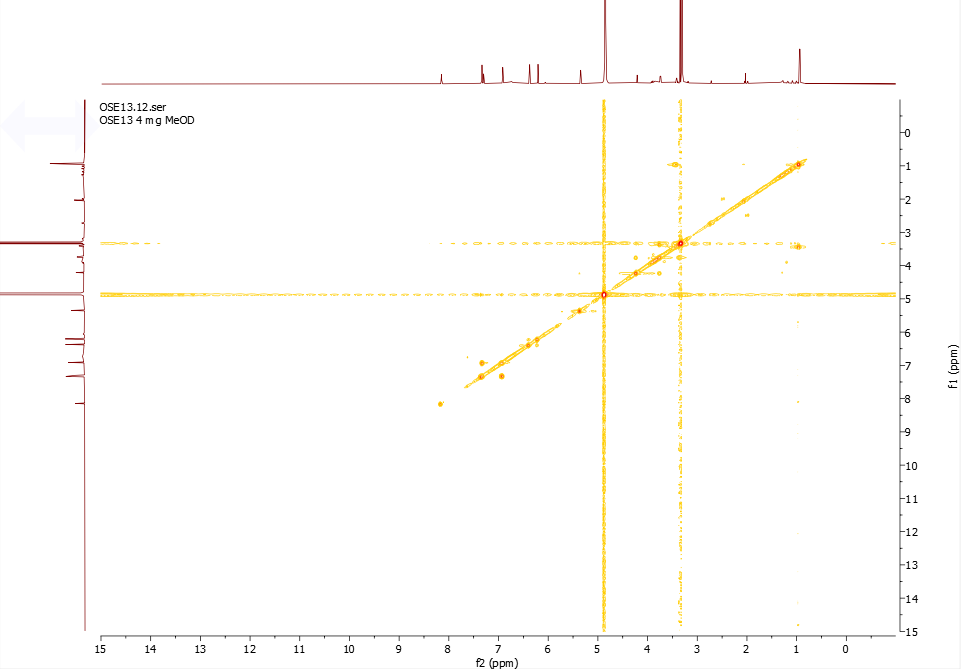


Figure S11. COSY (600 MHz, CD_3_OD) spectrum of 2


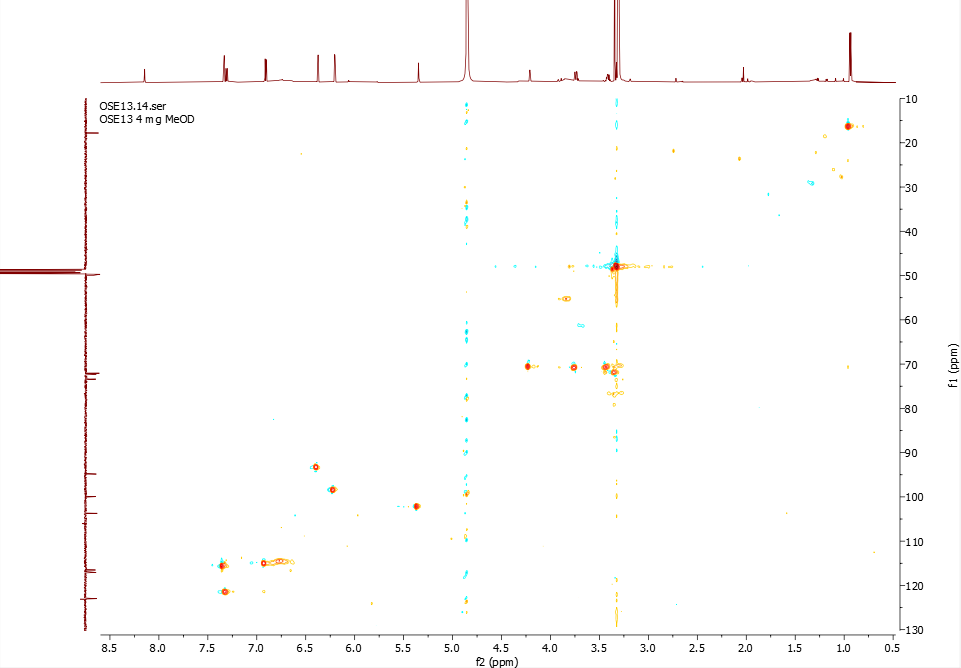


Figure S12. HSQC (600 MHz, CD_3_OD) spectrum of 2


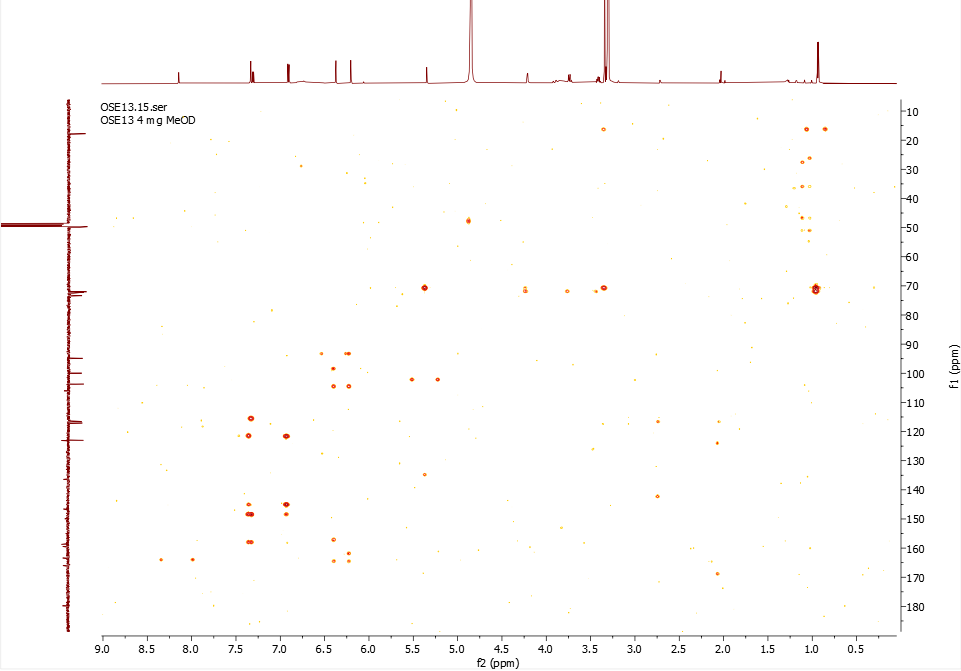


Figure S13. HMBC (600 MHz, CD_3_OD) spectrum of 2


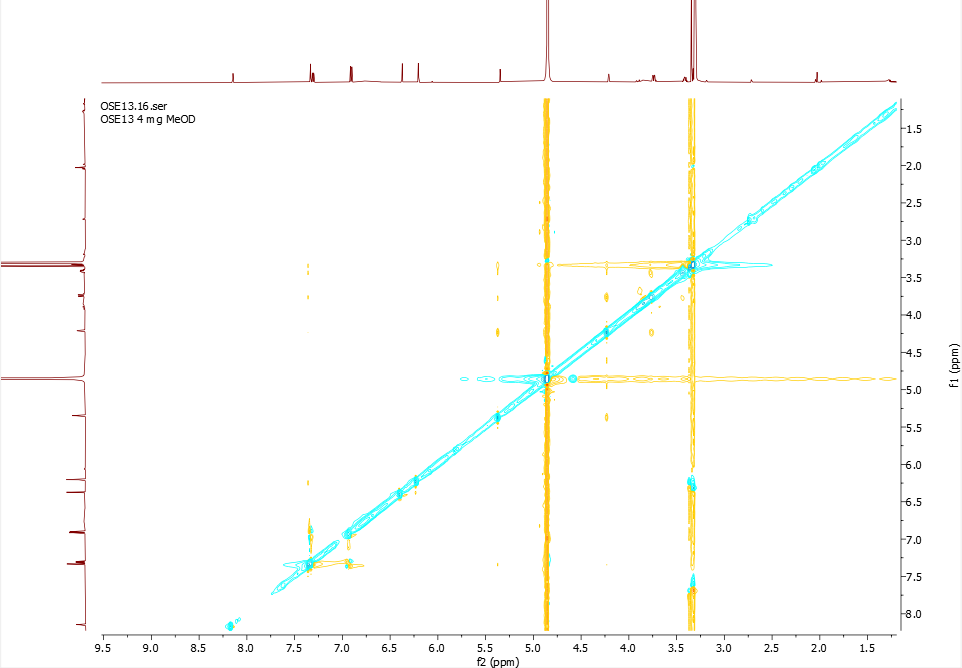


Figure S14. ROESY (600 MHz, CD_3_OD) spectrum of 2


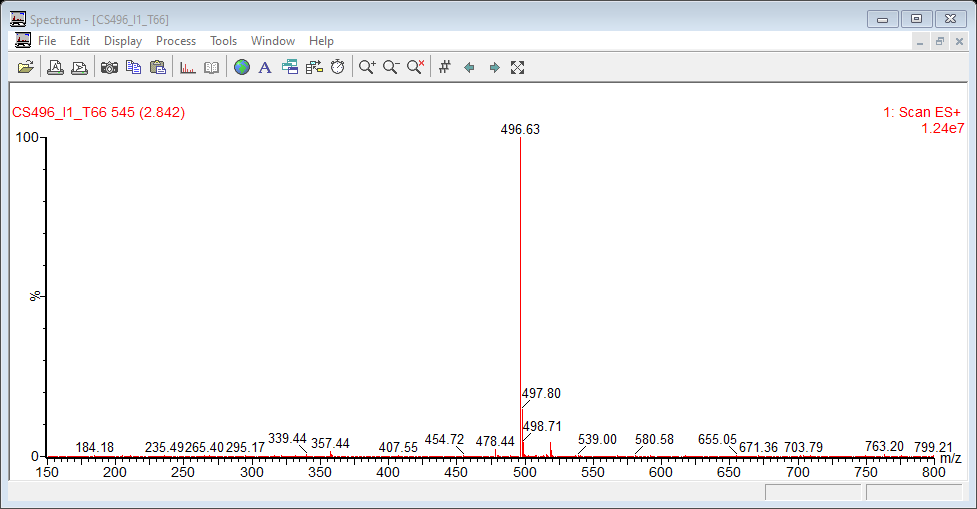


Figure S15. ESIMS spectrum of 3

**
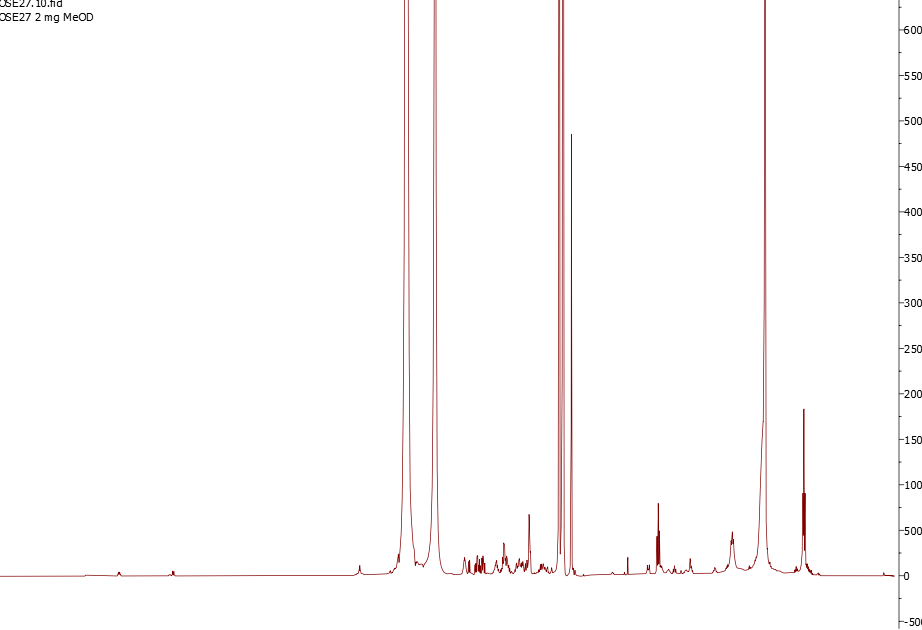
**

**Figure S16.** ^1^H NMR (600 MHz, CD_3_OD) spectrum of **3**


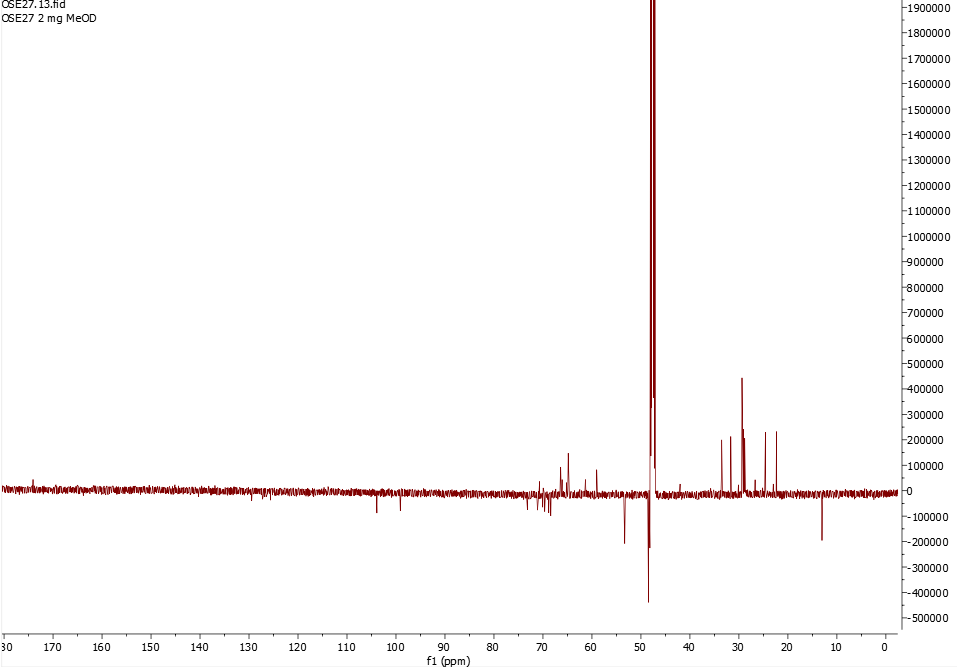


**Figure S17.** DEPTQ NMR (150 MHz, CD_3_OD) spectrum of **3**


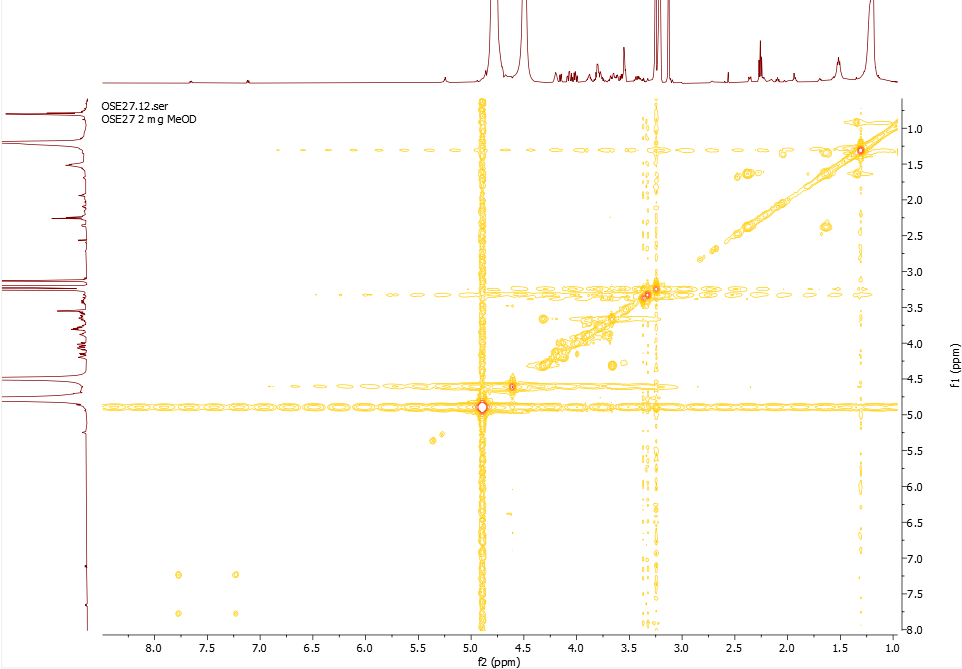


Figure S18. COSY (600 MHz, CD_3_OD) spectrum of 3


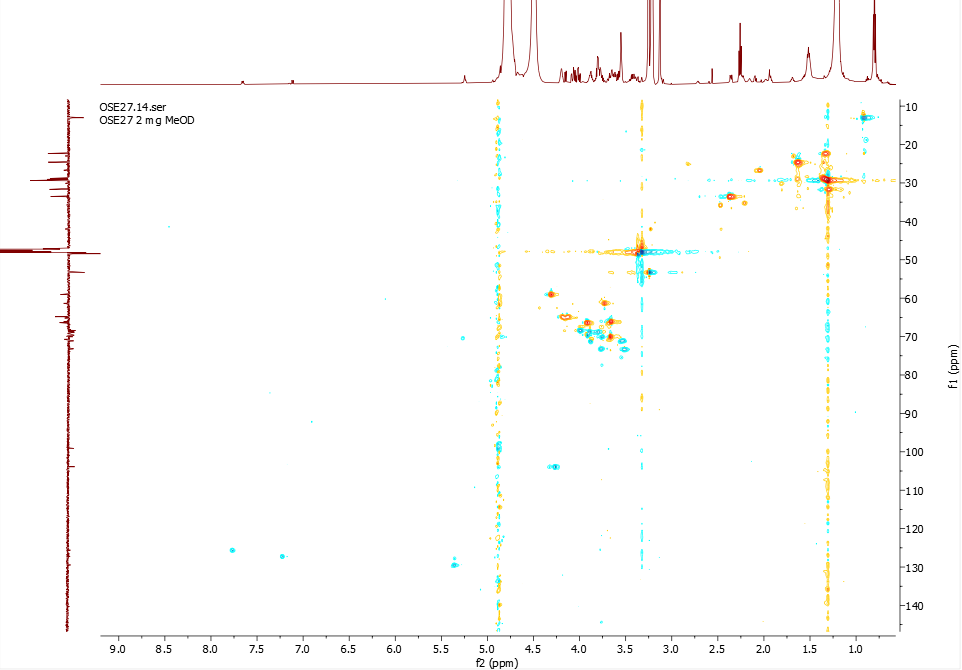


Figure S19. HSQC (600 MHz, CD_3_OD) spectrum of 3


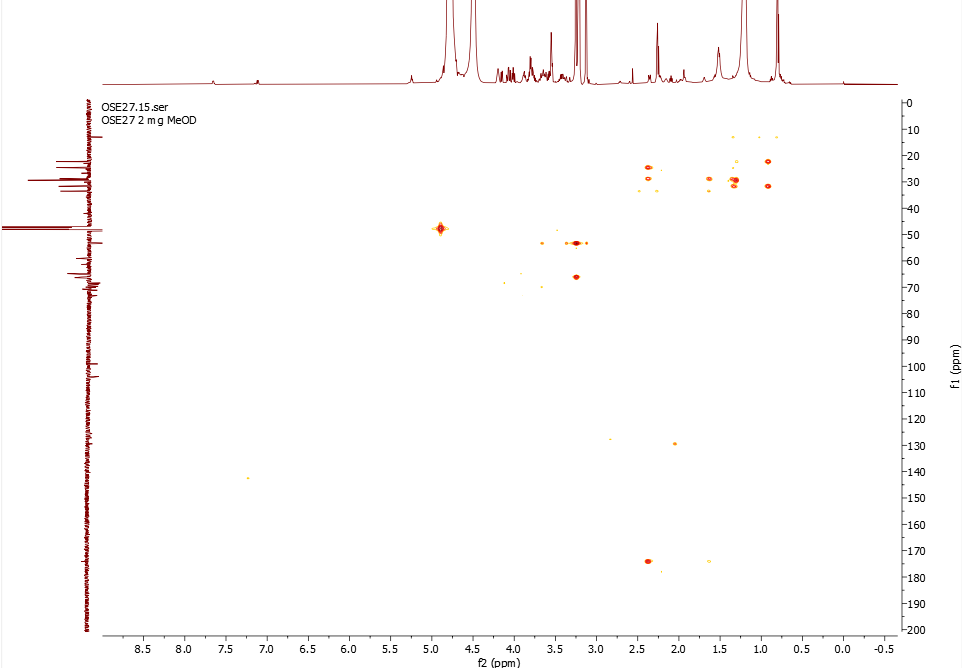


Figure S20. HMBC (600 MHz, CD_3_OD) spectrum of 3

^
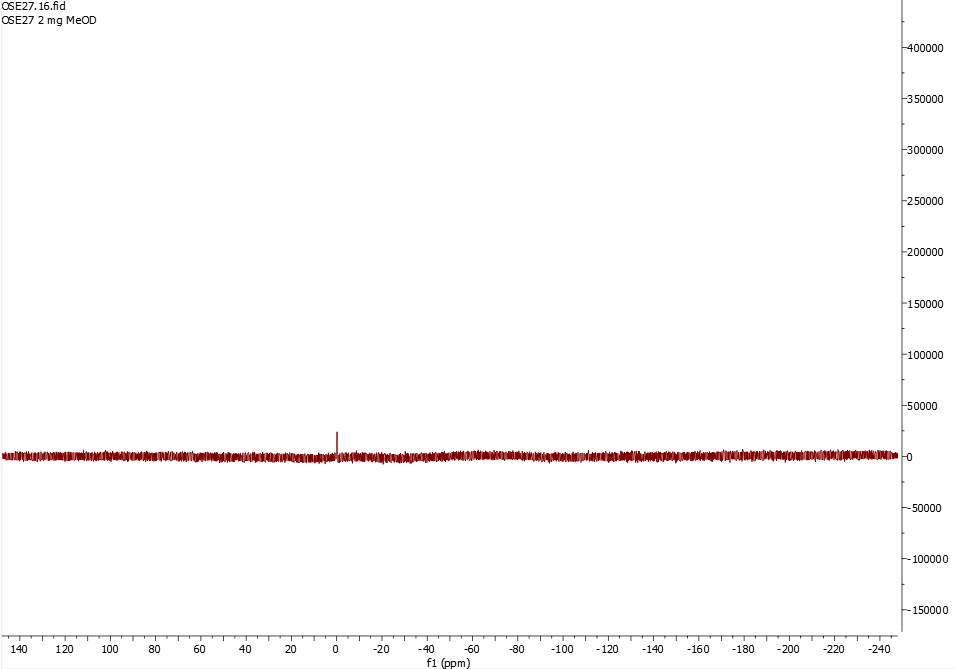
^

Figure S21. ^31^P (600 MHz, CD_3_OD) spectrum of 3


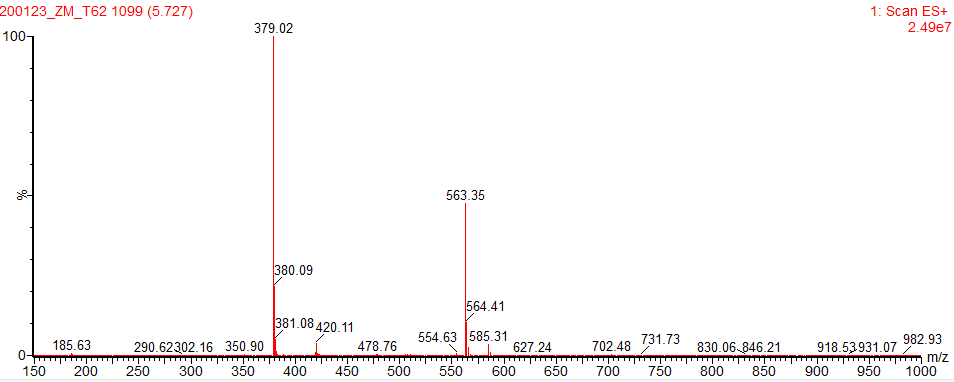


Figure S22. ESIMS spectrum of 4


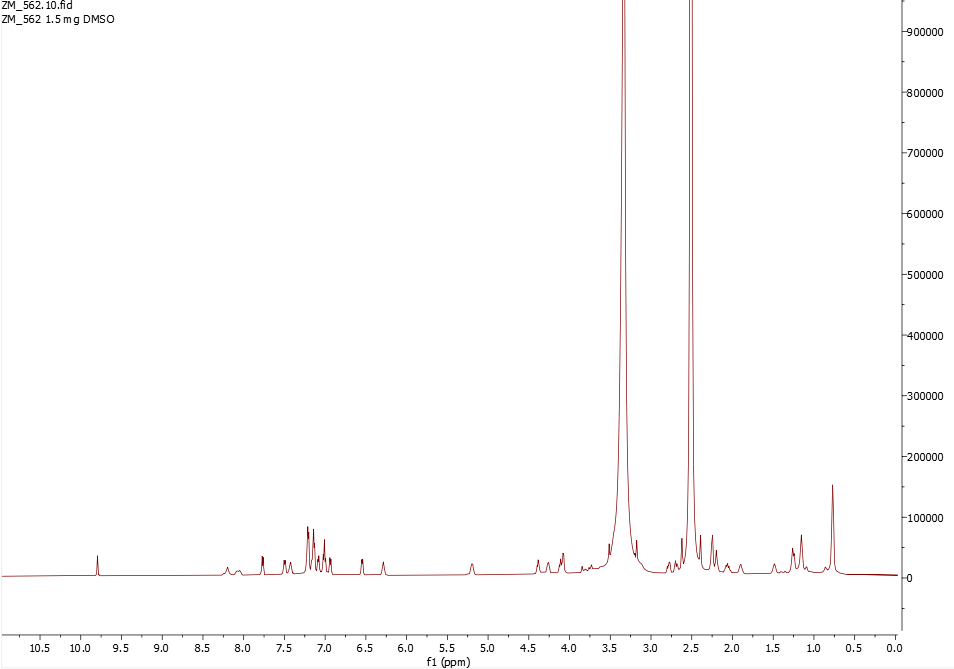


Figure S23. ^1^H NMR (600 MHz, DMSO-*d_6_*) spectrum of 4


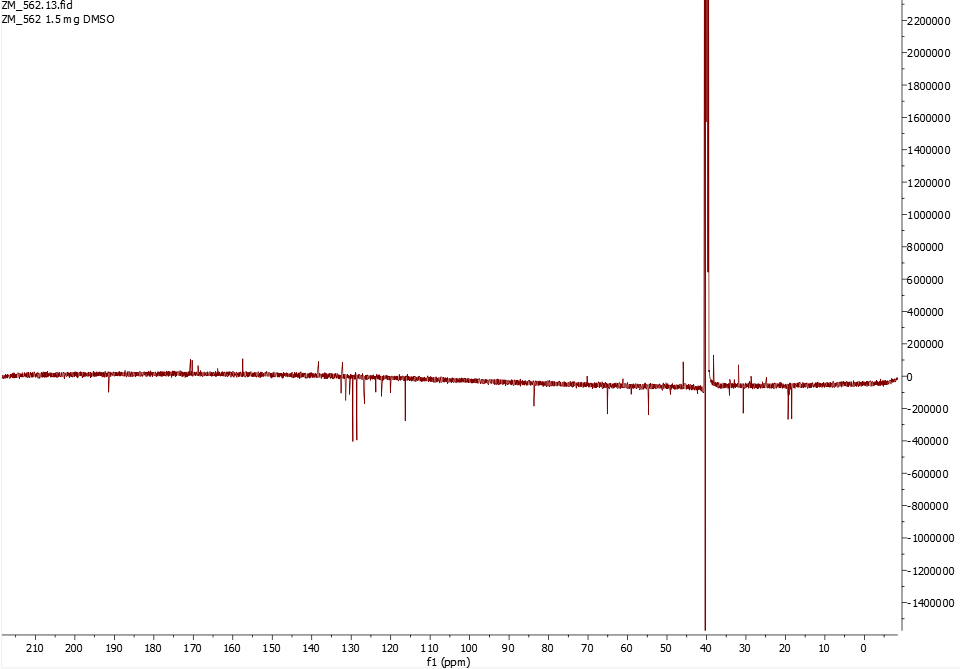


Figure S24. DEPTQ NMR (150 MHz, DMSO-*d_6_*) spectrum of 4


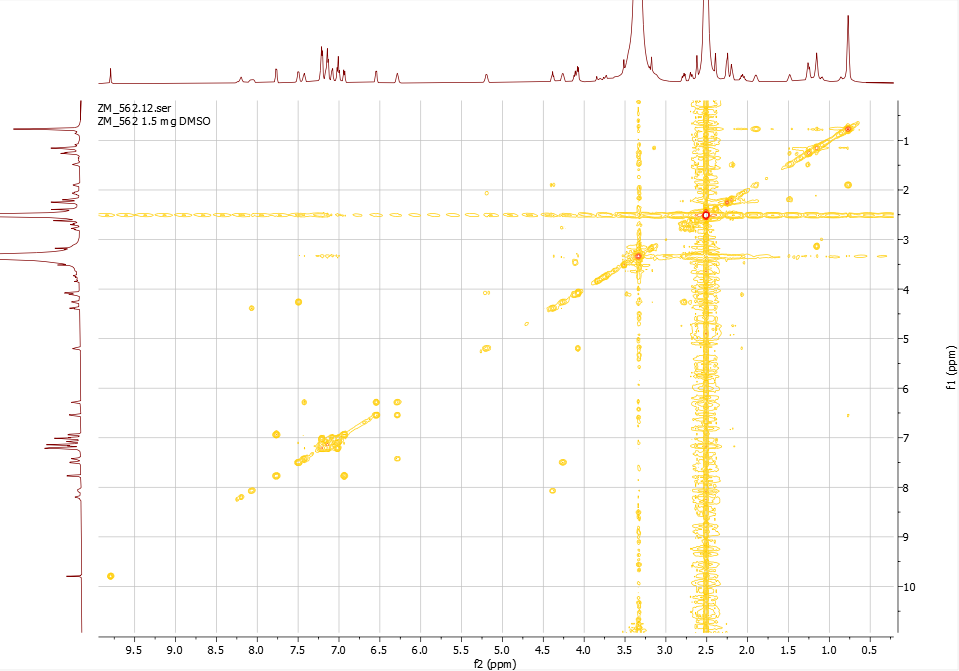


Figure S25. COSY (600 MHz, DMSO-*d_6_*) spectrum of 4


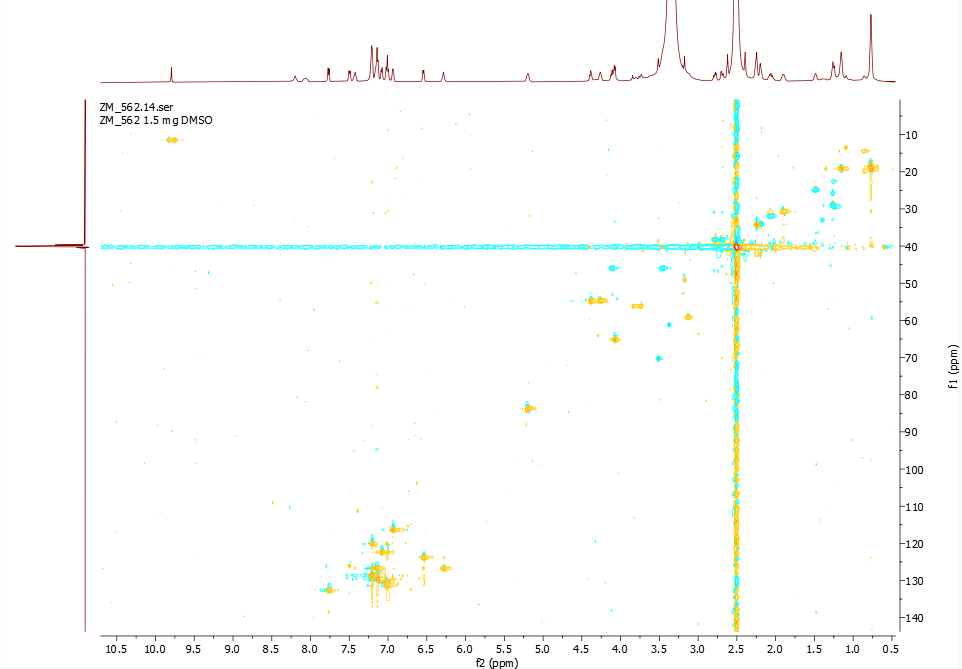


Figure S26. HSQC (600 MHz, DMSO-*d_6_*) spectrum of 4


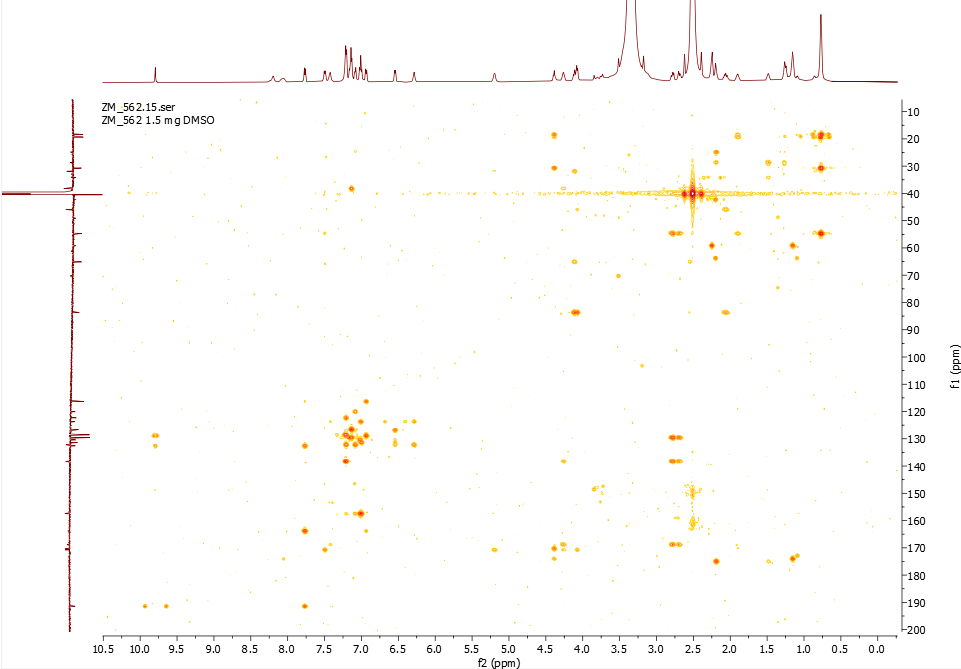


Figure S27. HMBC (600 MHz, DMSO-*d_6_*) spectrum of 4

**
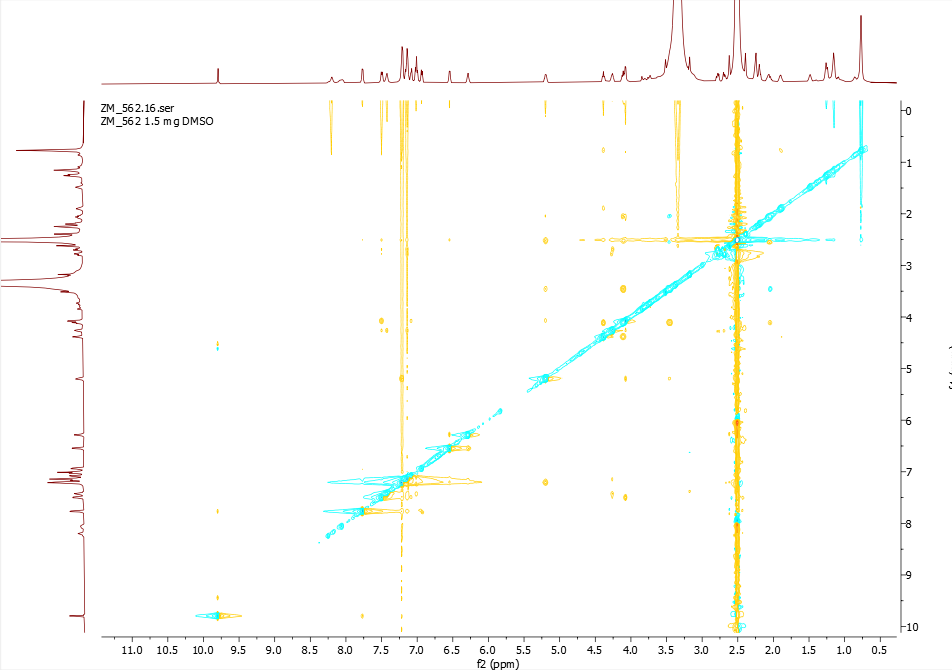
**

Figure S28. ROESY (600 MHz, DMSO-*d_6_*) spectrum of 4


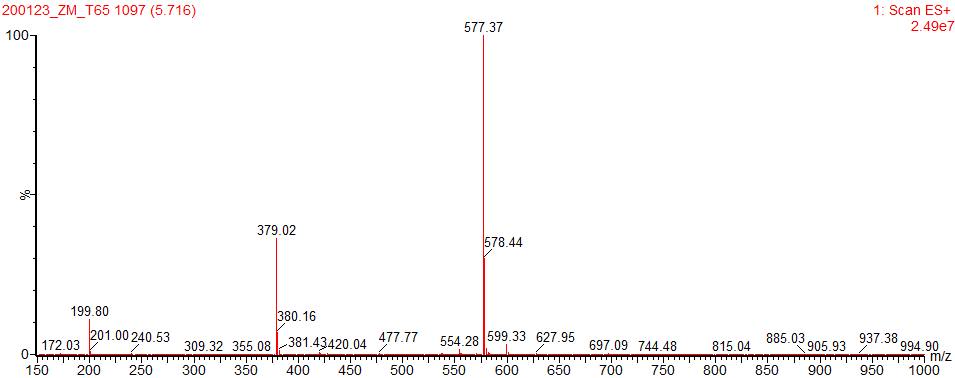


Figure S29. ESIMS spectrum of 5


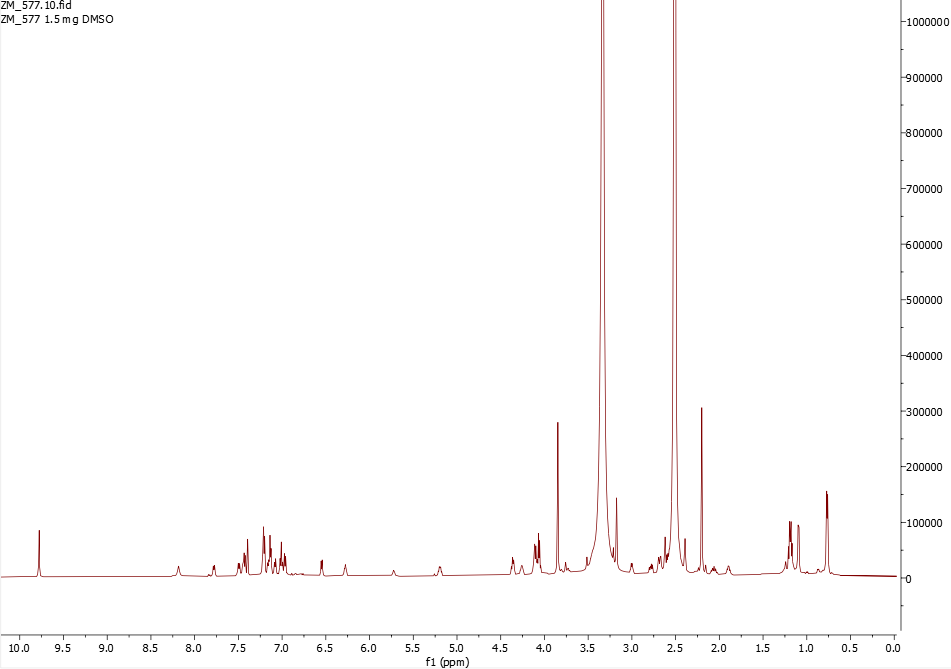


Figure S30. ^1^H NMR (600 MHz, DMSO-*d_6_*) spectrum of 5


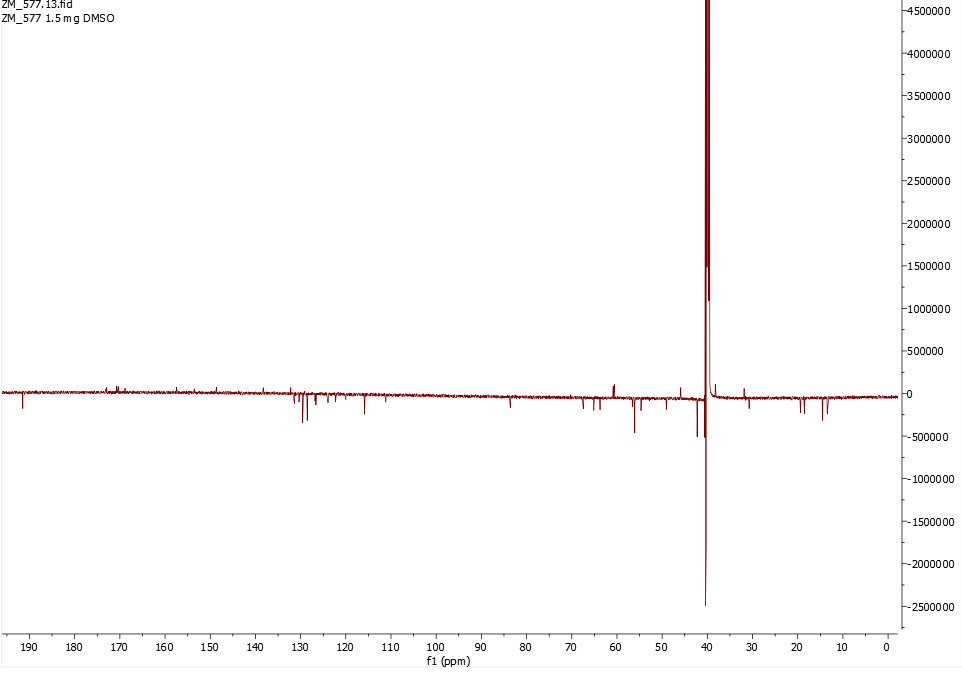


Figure S31. DEPTQ NMR (150 MHz, DMSO-*d_6_*) spectrum of 5


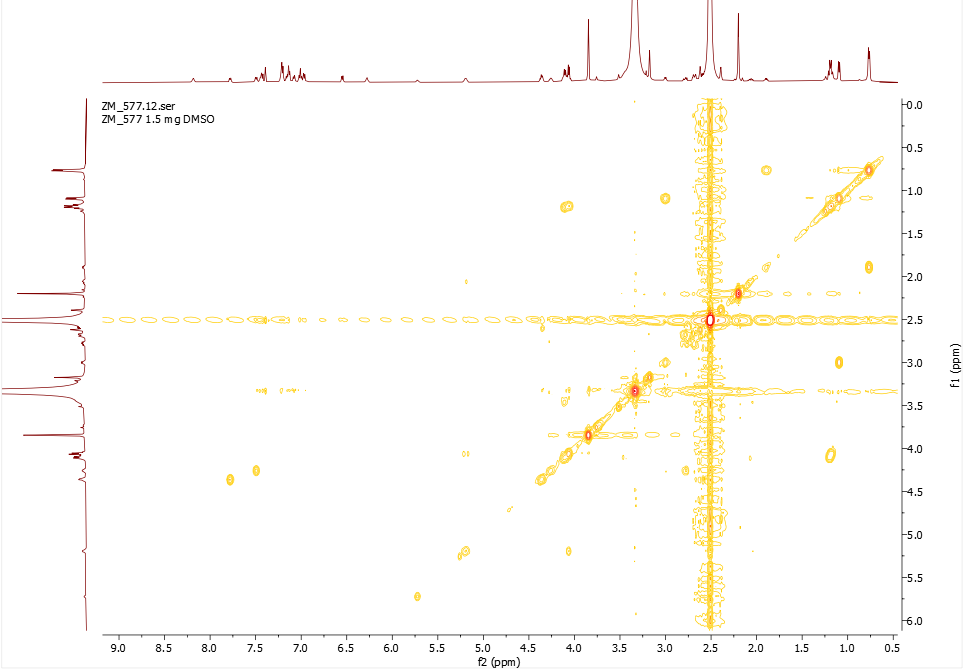


**Figure S32.** COSY (600 MHz, DMSO-*d_6_*) spectrum of **5**


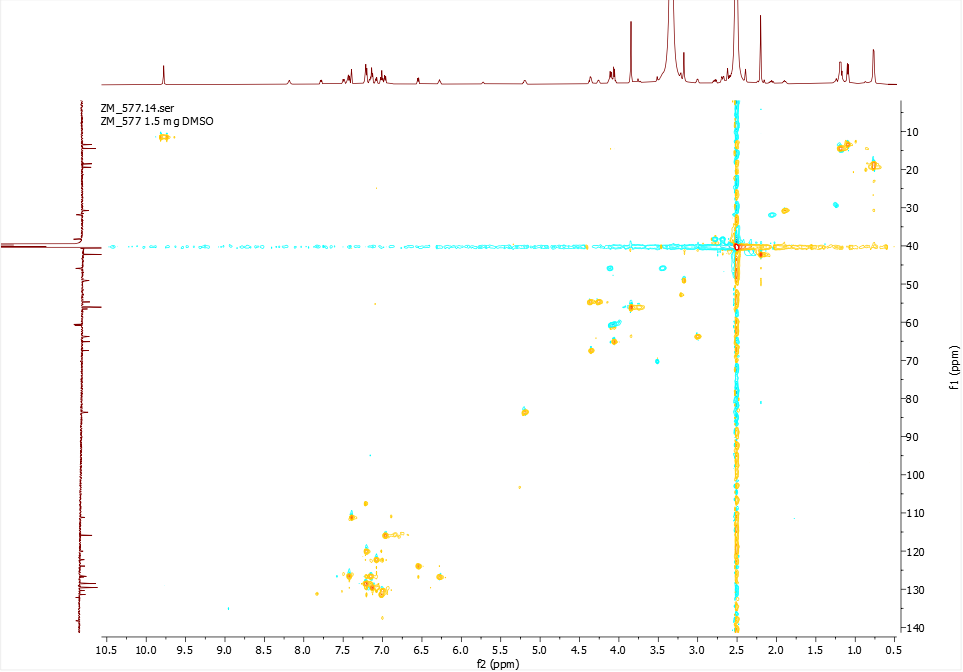


Figure S33. HSQC (600 MHz, DMSO-*d_6_*) spectrum of 5


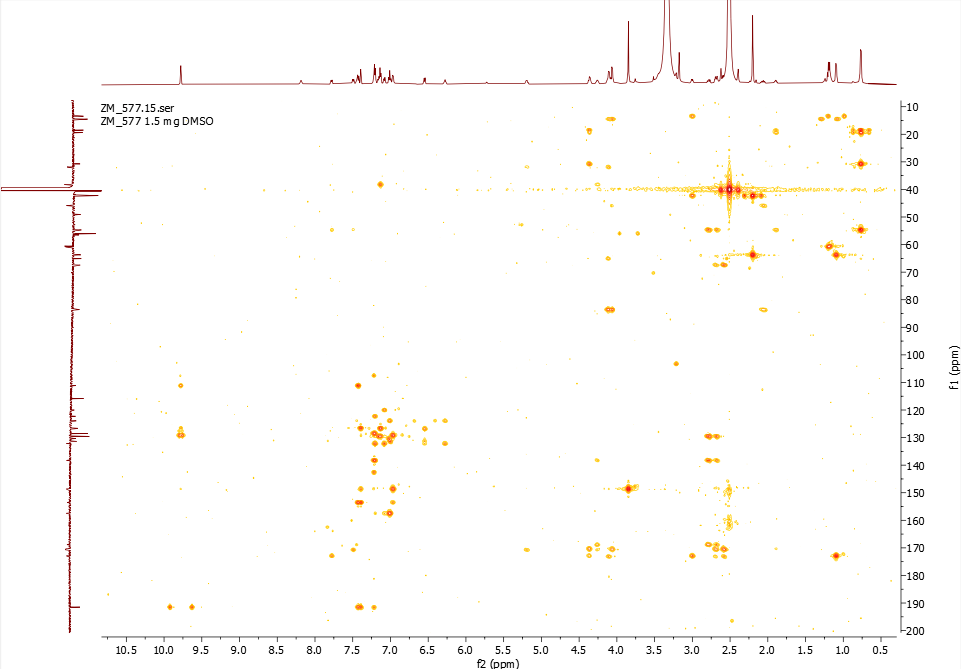


Figure S34. HMBC (600 MHz, DMSO-*d_6_*) spectrum of 5

**
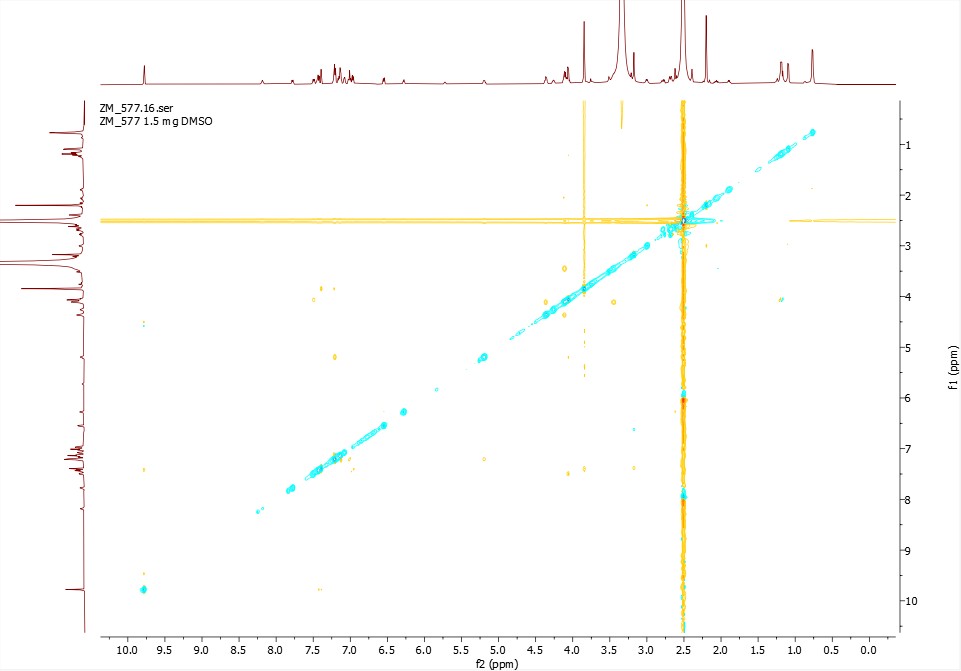
**

Figure S35. ROESY (600 MHz, DMSO-*d_6_*) spectrum of 5


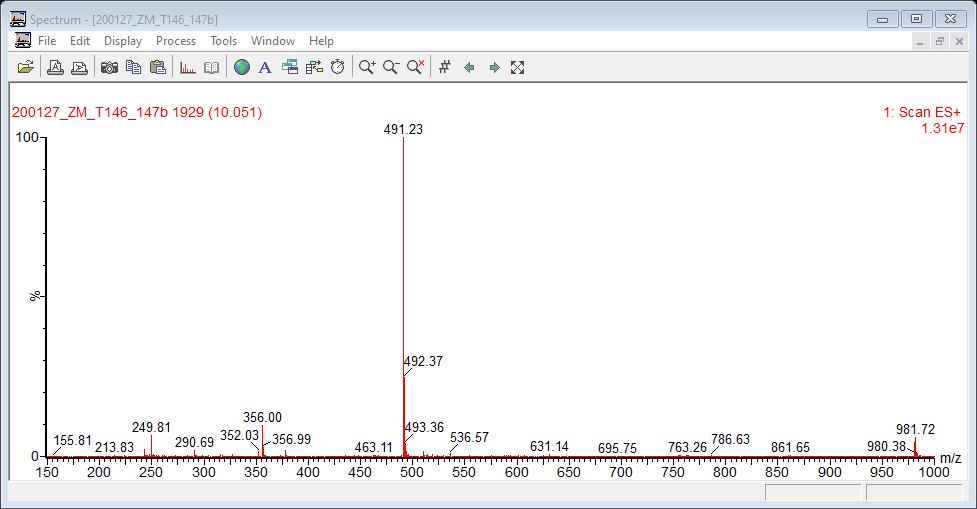


Figure S36. ESIMS spectrum of 6


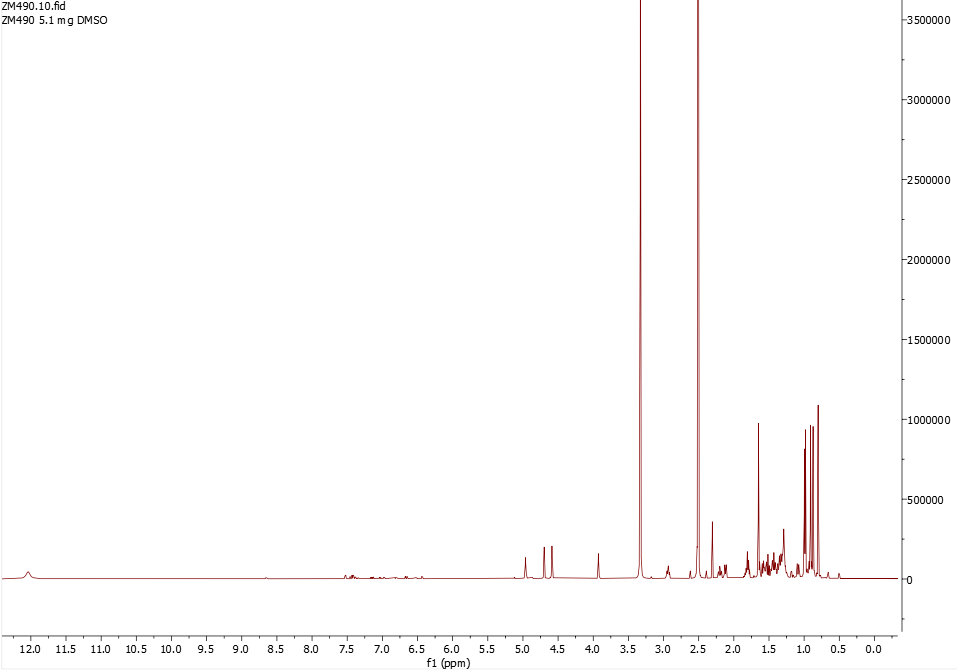


Figure S37. ^1^H NMR (600 MHz, DMSO-*d_6_*) spectrum of 6


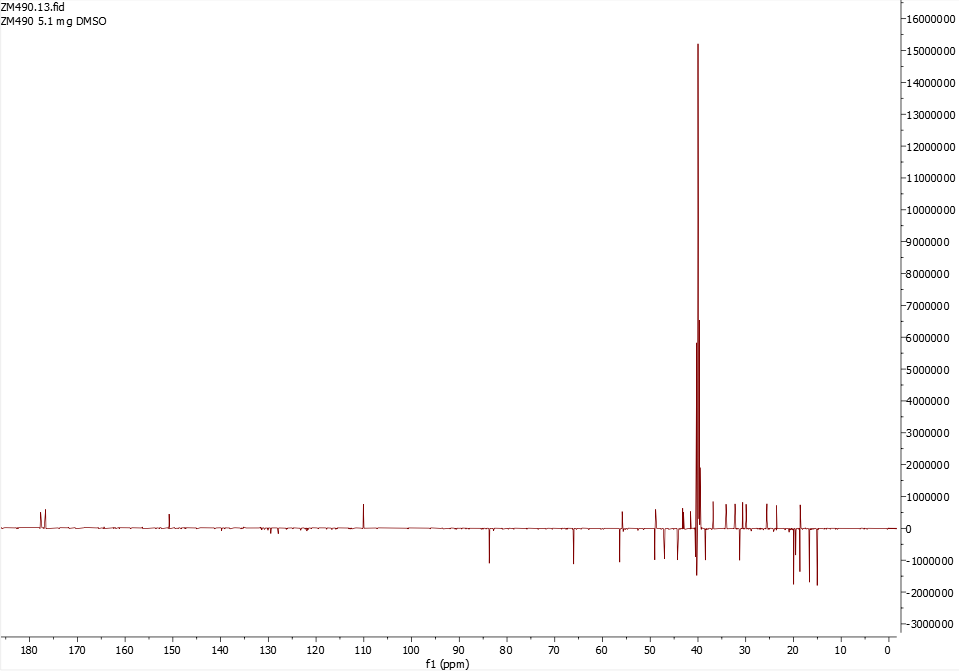


Figure S38. DEPTQ NMR (150 MHz, DMSO-*d_6_*) spectrum of 6


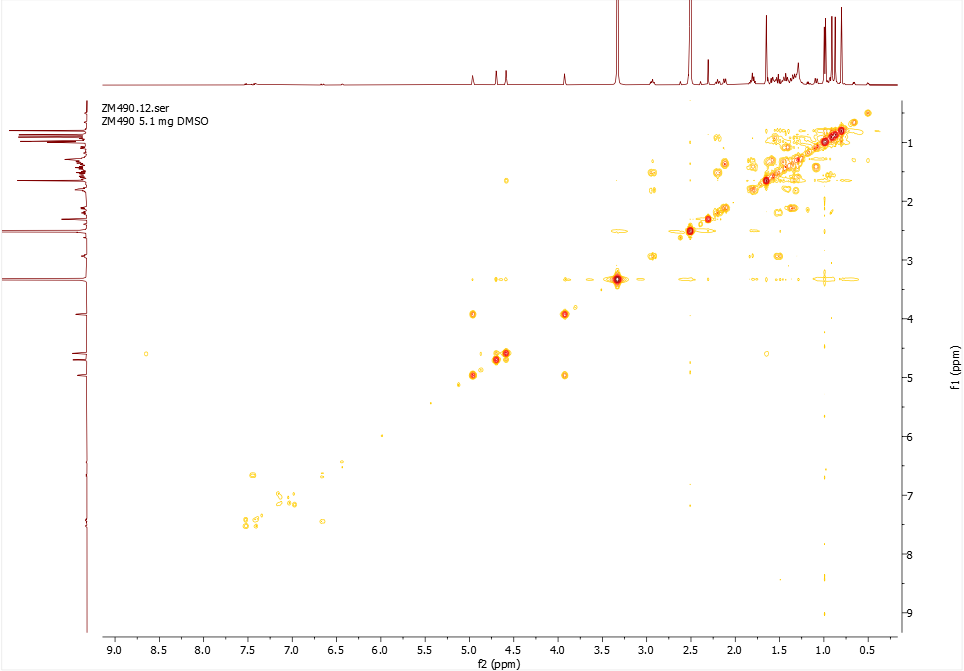


Figure S39. COSY (600 MHz, DMSO-*d_6_*) spectrum of 6


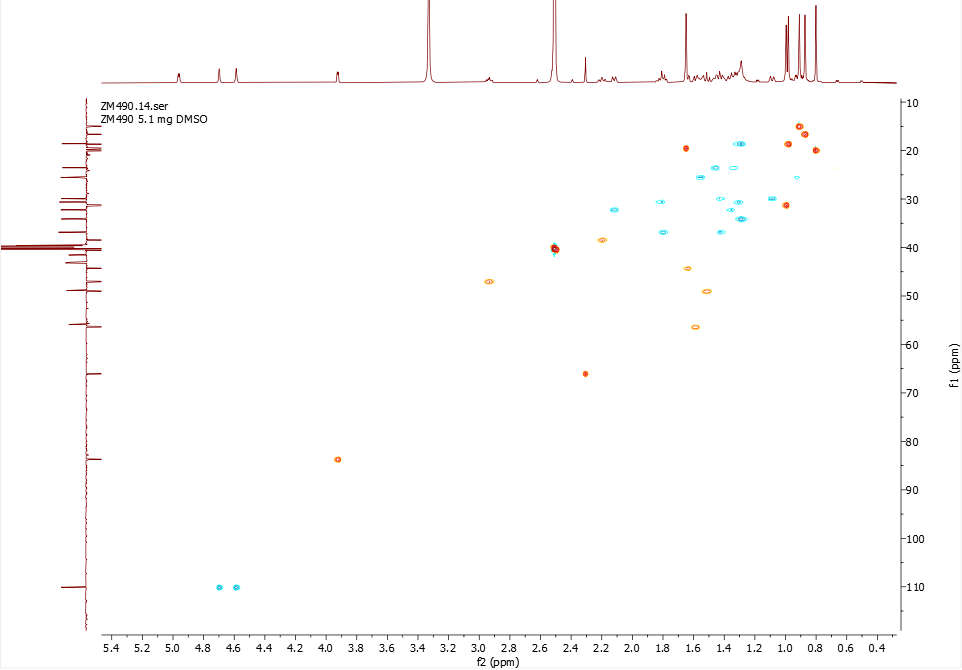


Figure S40. HSQC (600 MHz, DMSO-*d_6_*) spectrum of 6


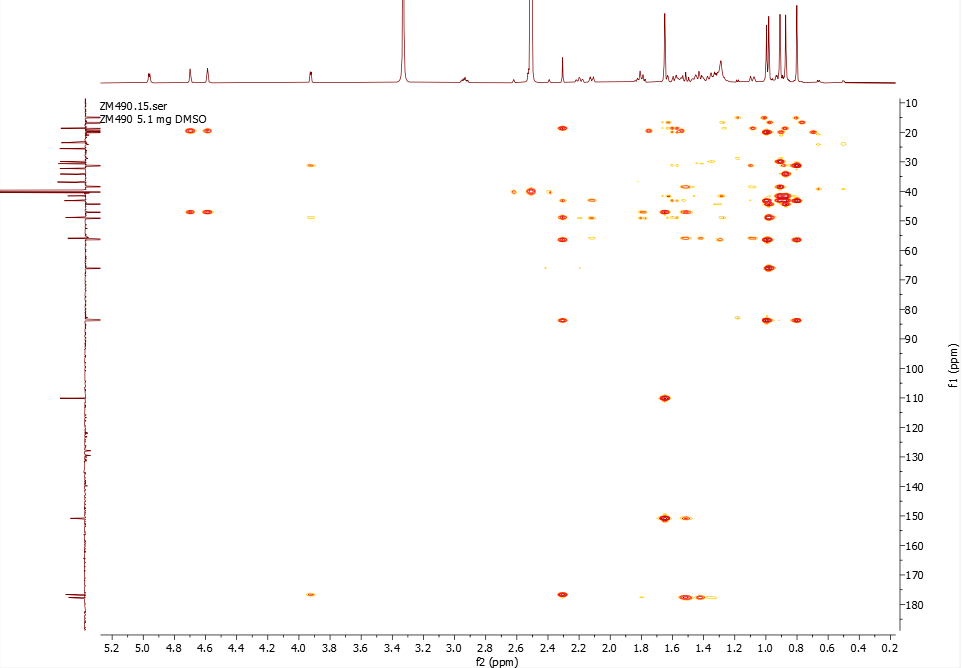


Figure S41. HMBC (600 MHz, DMSO-*d_6_*) spectrum of 6

**
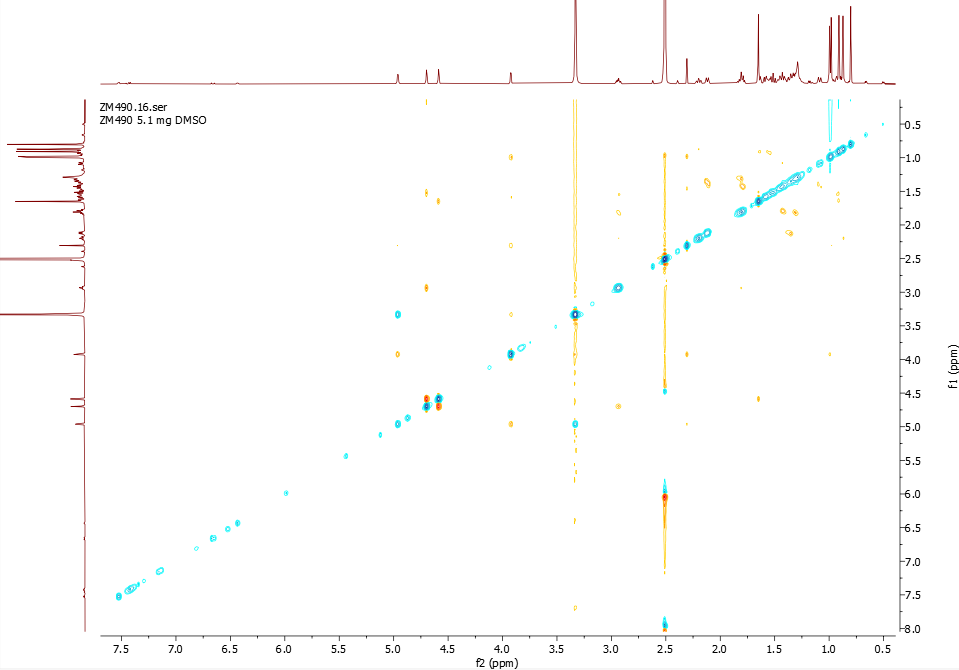
**

Figure S42. ROESY (600 MHz, DMSO-*d_6_*) spectrum of 6


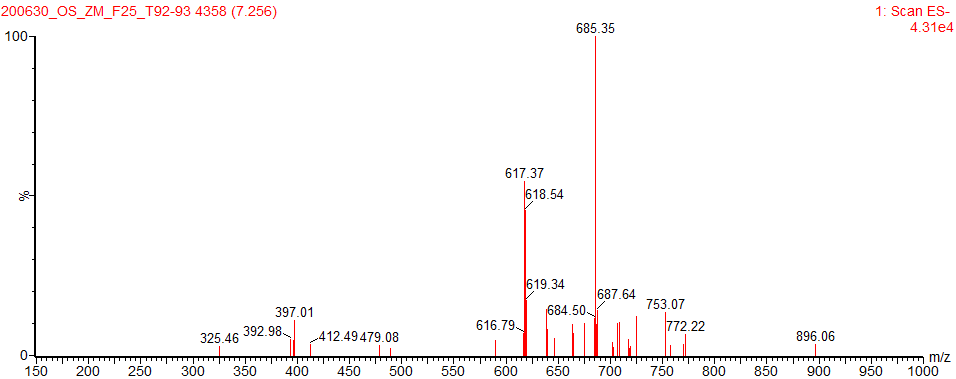


Figure S43. ESIMS spectrum of 7


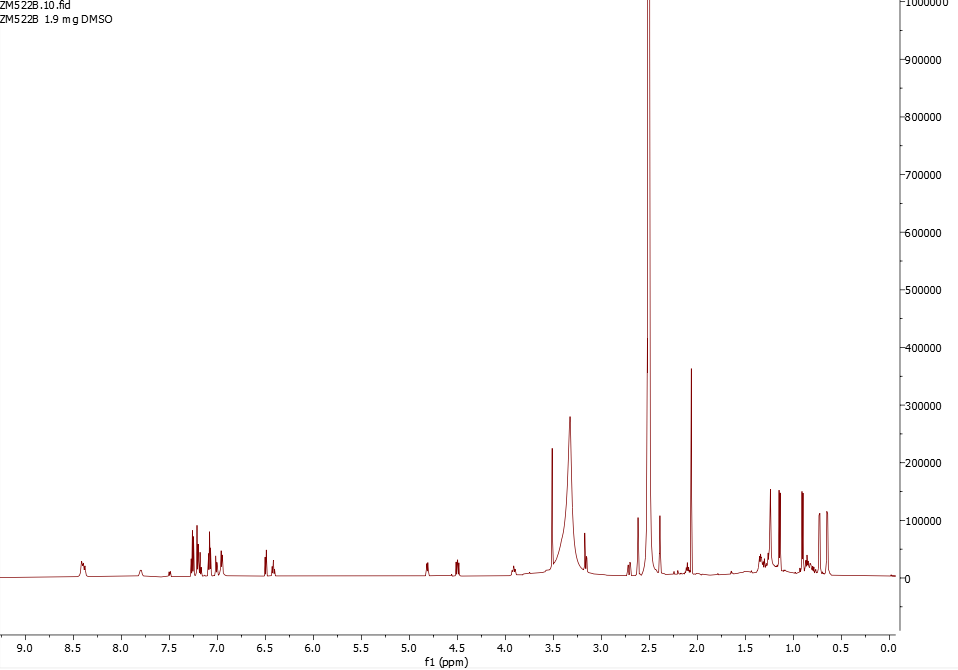


Figure S44. ^1^H NMR (600 MHz, DMSO-*d_6_*) spectrum of 7


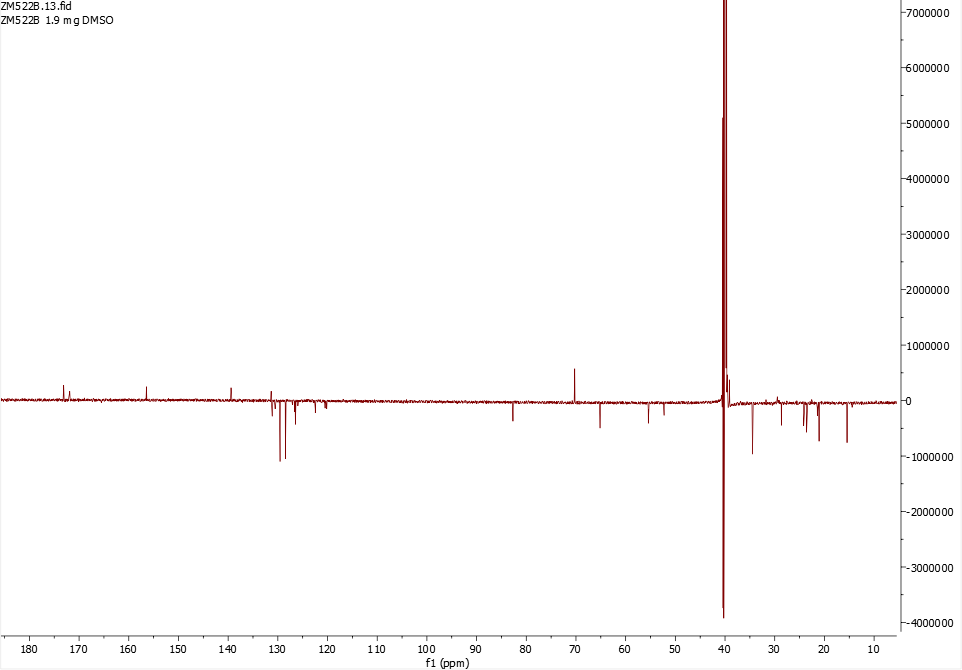


Figure S45. DEPTQ NMR (150 MHz, DMSO-*d_6_*) spectrum of 7


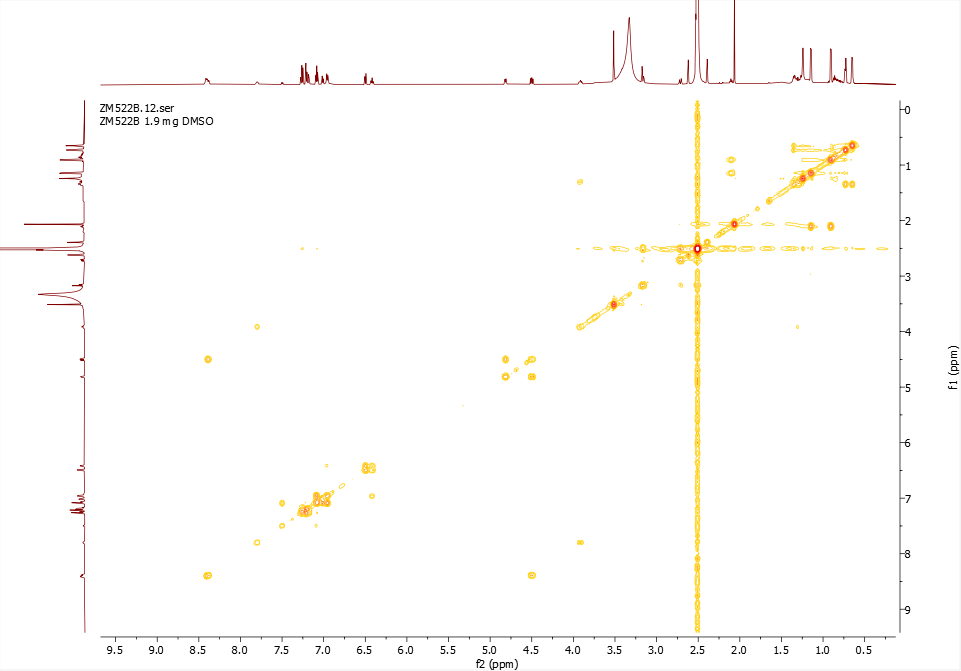


Figure S46. COSY (600 MHz, DMSO-*d_6_*) spectrum of 7


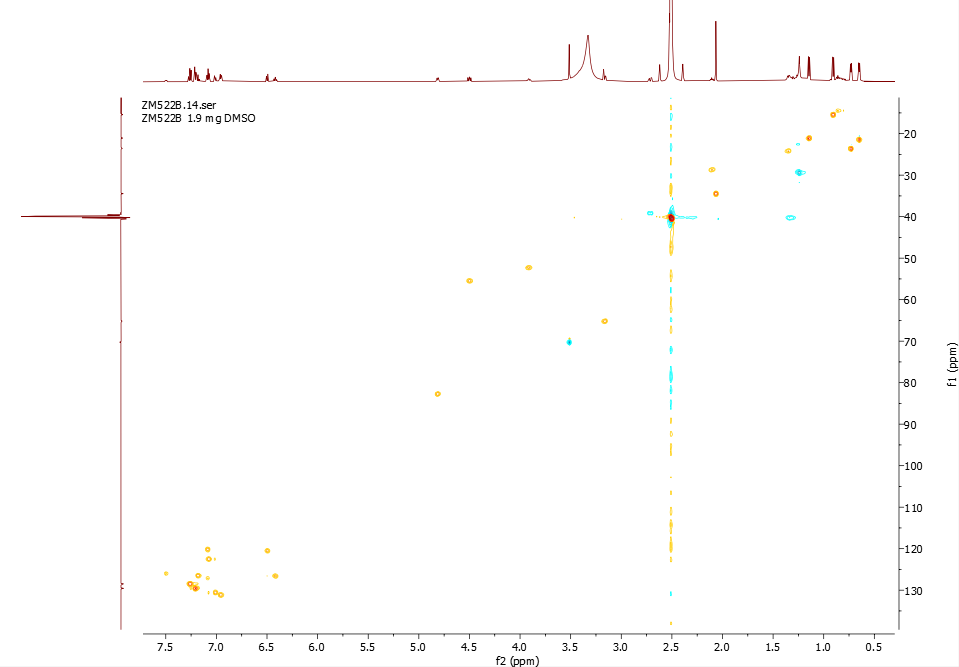


Figure S 47. HSQC (600 MHz, DMSO-*d_6_*) spectrum of 7


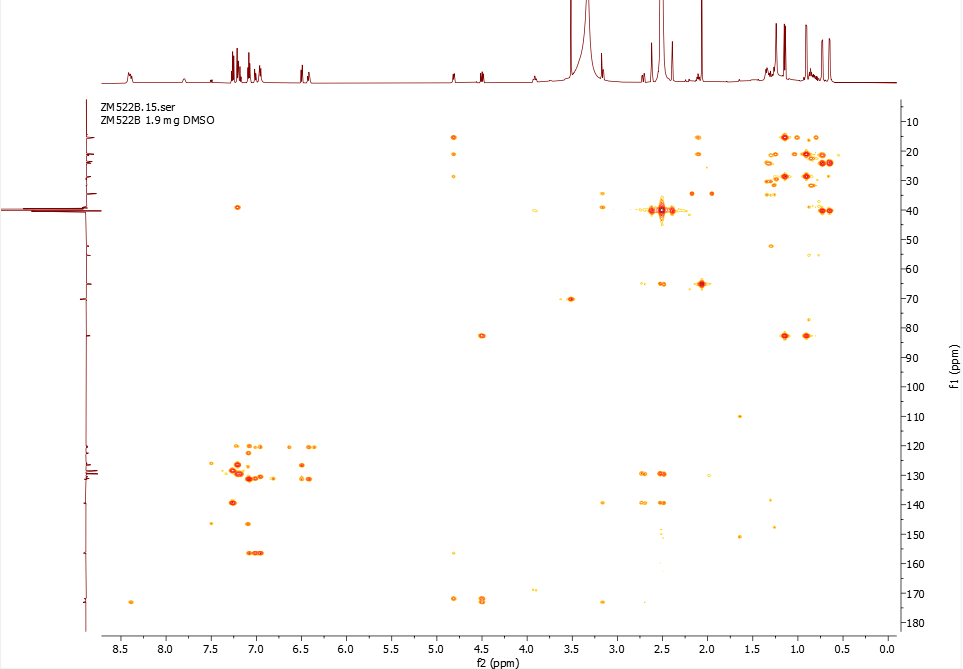


Figure S48. HMBC (600 MHz, DMSO-*d_6_*) spectrum of 7

**
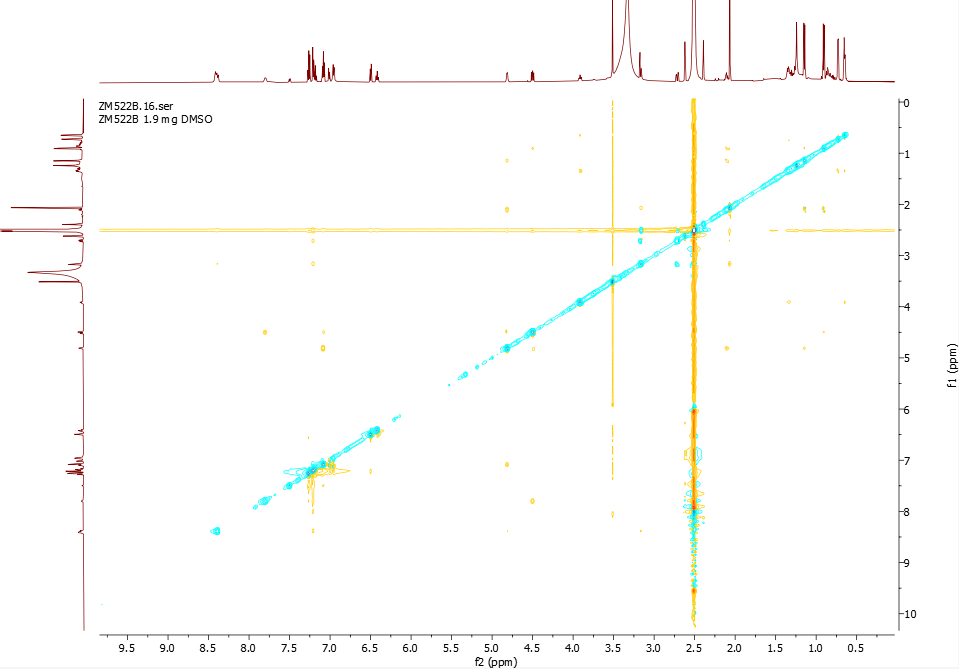
**

Figure S49. ROESY (600 MHz, DMSO-*d_6_*) spectrum of 7


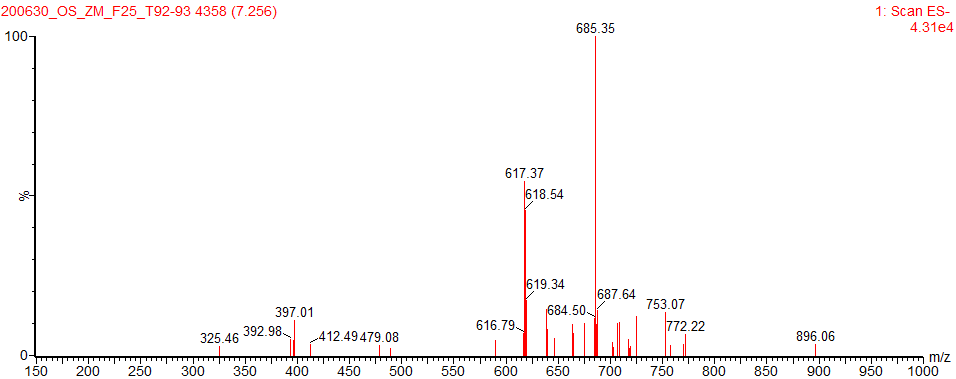


Figure S50. ESIMS spectrum of 8


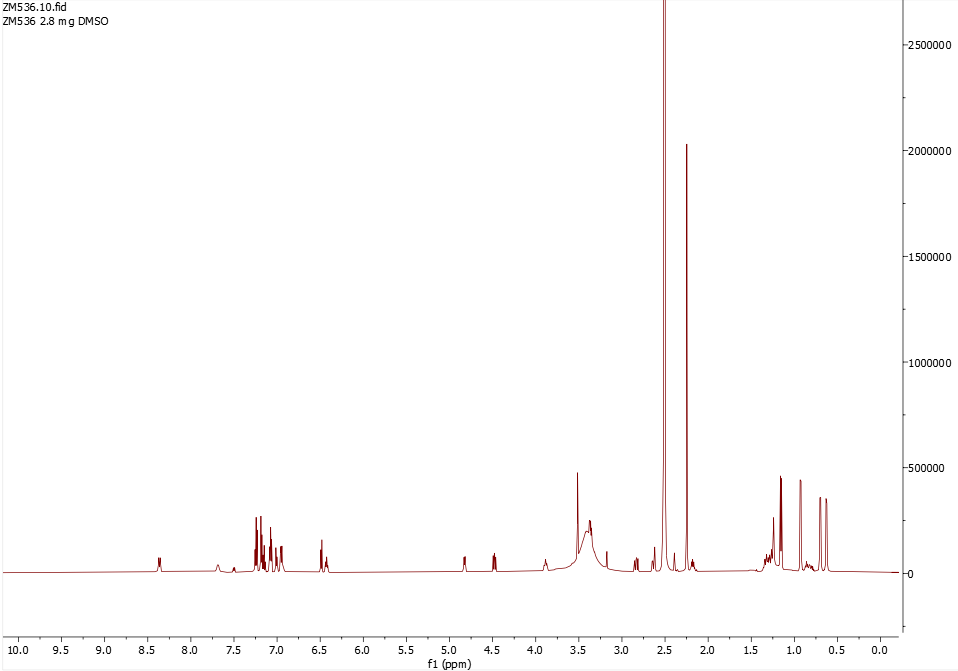


Figure S51. ^1^H NMR (600 MHz, DMSO-*d_6_*) spectrum of 8


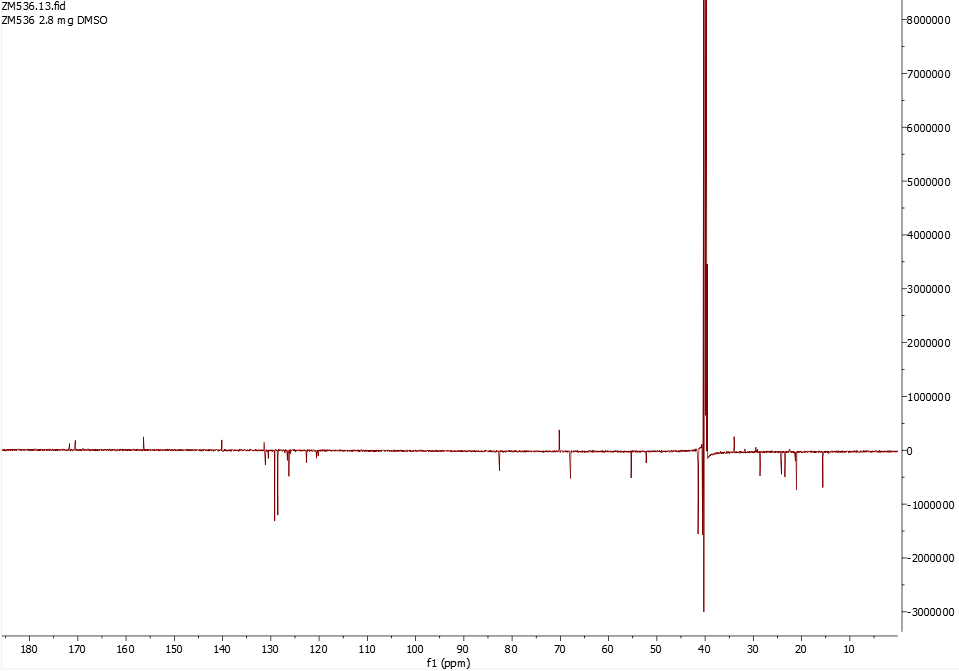


Figure S52. DEPTQ NMR (150 MHz, DMSO-*d_6_*) spectrum of 8


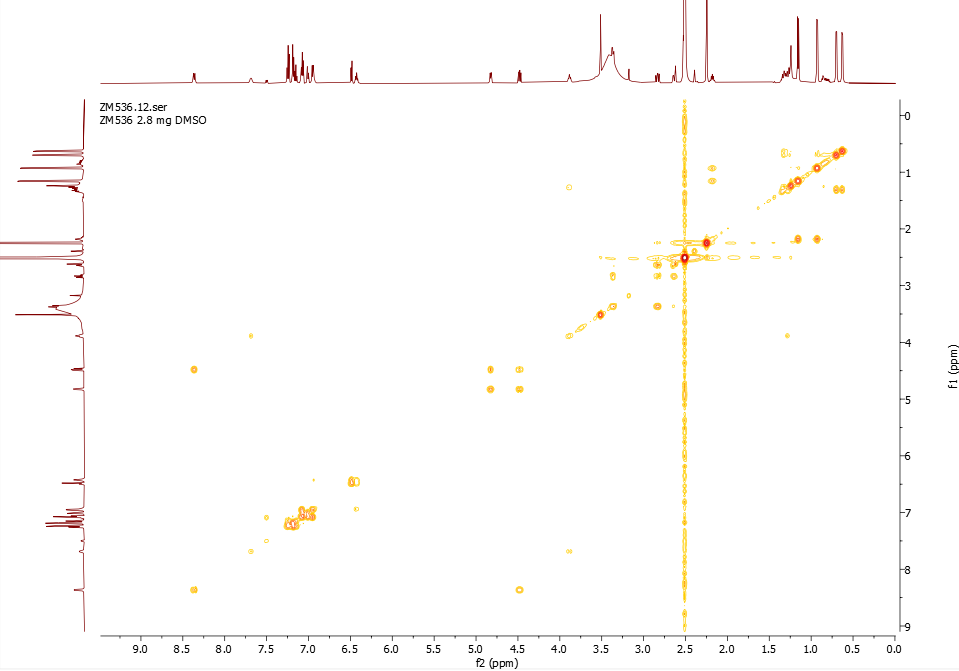


Figure S53. COSY (600 MHz, DMSO-*d_6_*) spectrum of 8


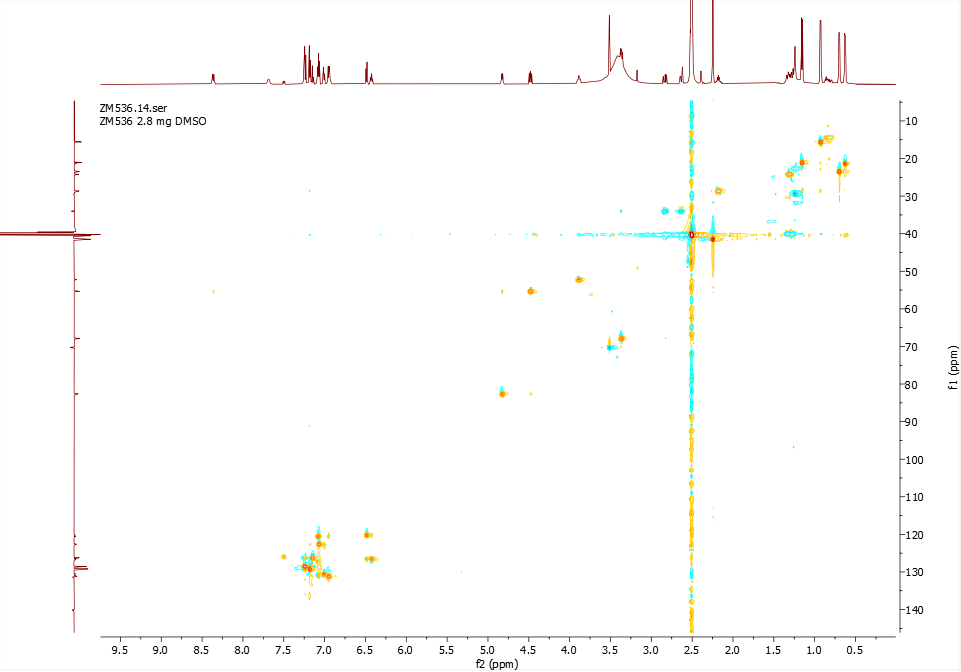


Figure S54. HSQC (600 MHz, DMSO-*d_6_*) spectrum of 8


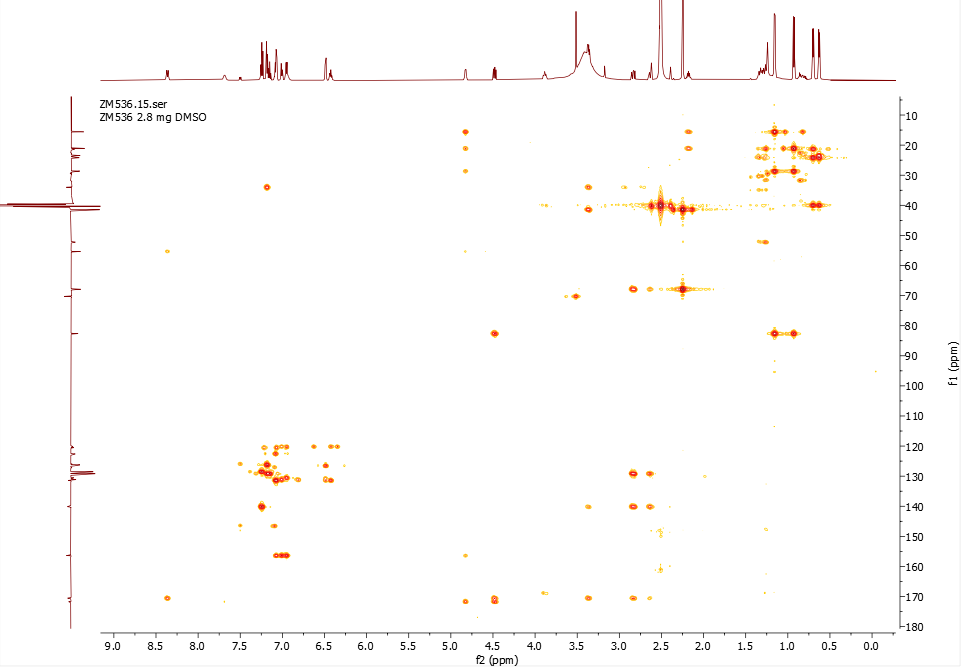


Figure S55. HMBC (600 MHz, DMSO-*d_6_*) spectrum of 8

**
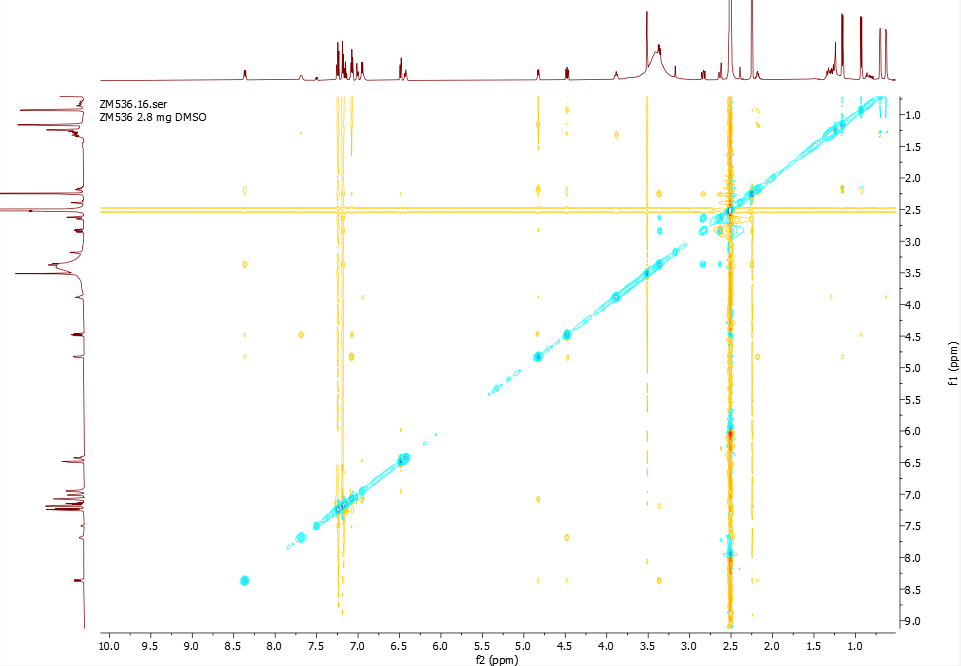
**

Figure S56. ROESY (600 MHz, DMSO-*d_6_*) spectrum of 8


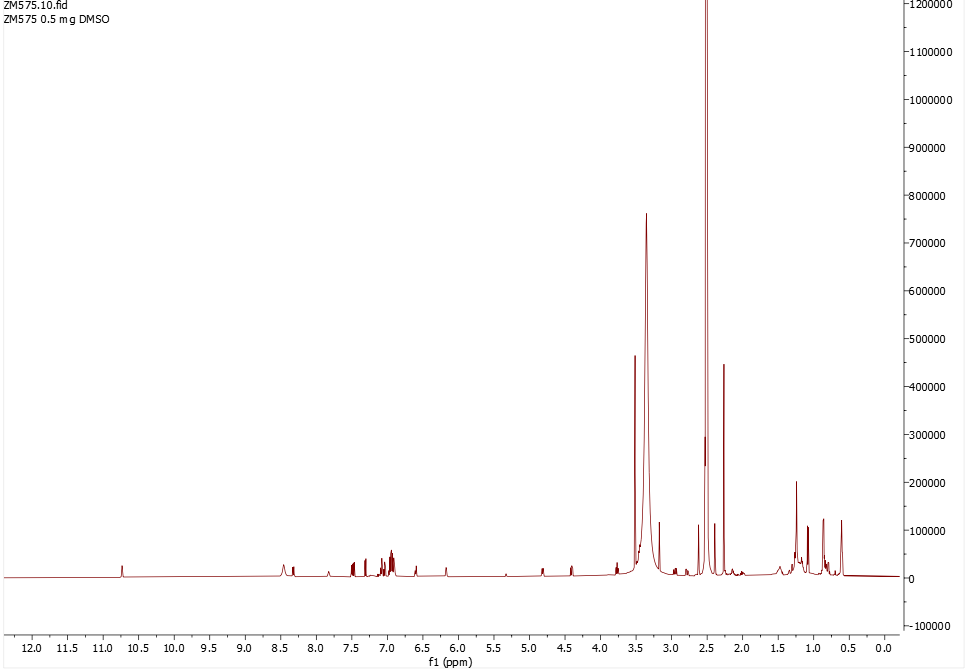


Figure S57. ^1^H NMR (600 MHz, DMSO-*d_6_*) spectrum of 9


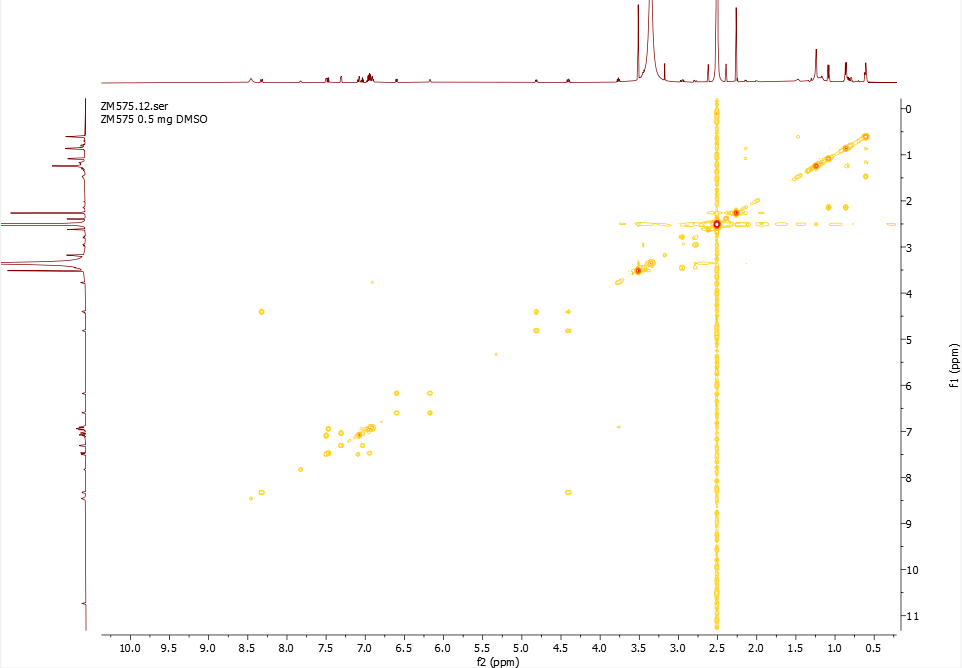


Figure S58. COSY (600 MHz, DMSO-*d_6_*) spectrum of 9


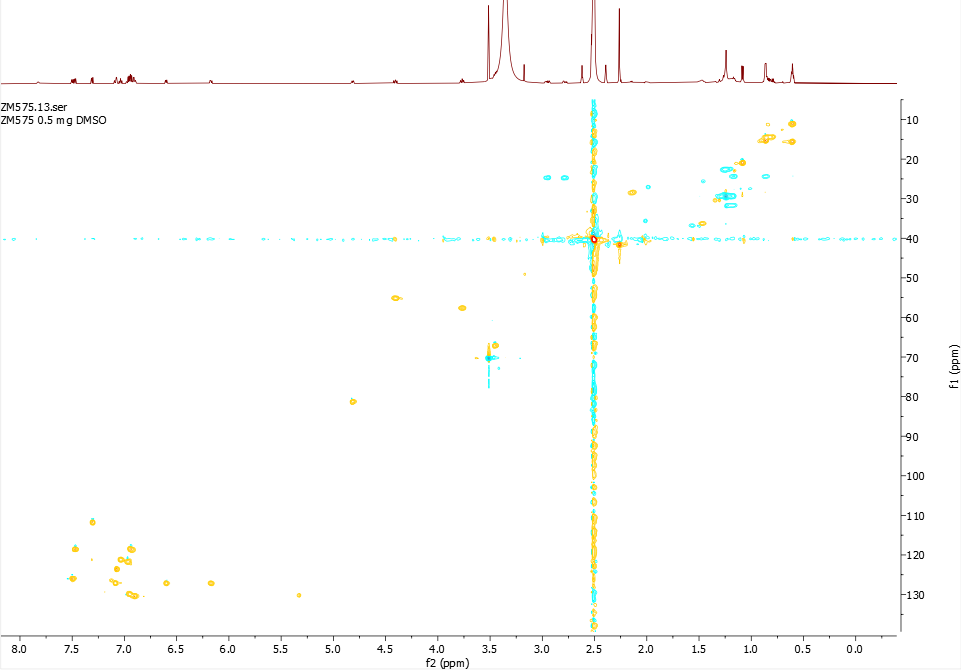


Figure S59. HSQC (600 MHz, DMSO-*d_6_*) spectrum of 9


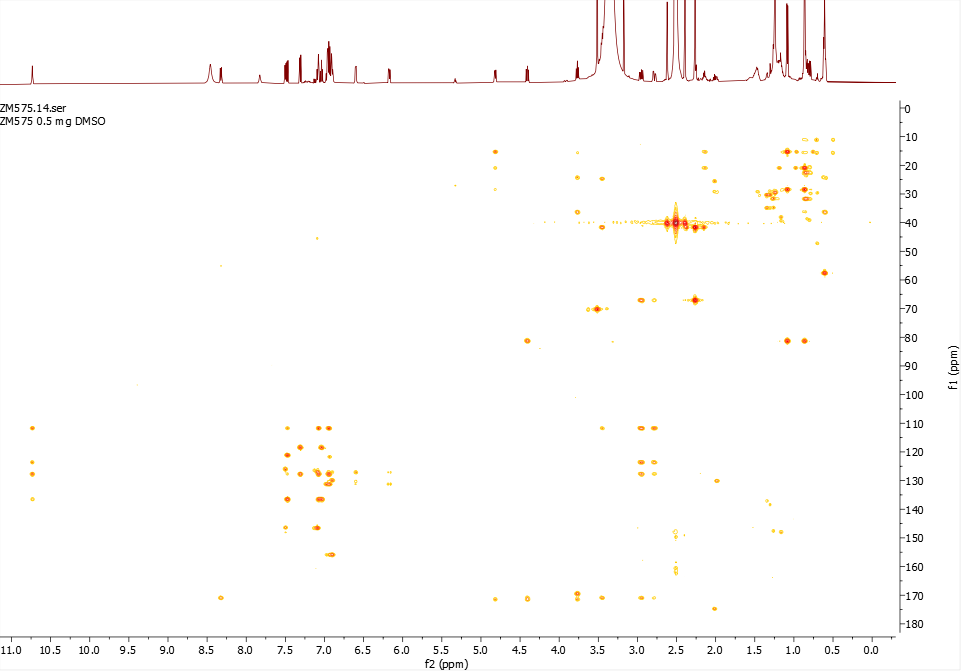


Figure S60. HMBC (600 MHz, DMSO-*d_6_*) spectrum of 9

**
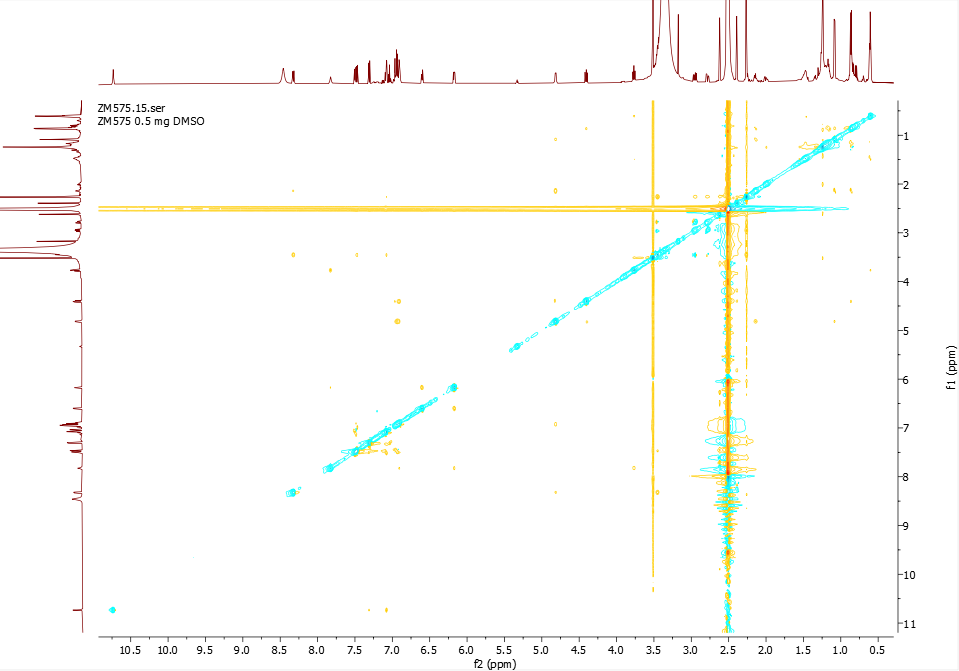
**

Figure S61. ROESY (600 MHz, DMSO-*d_6_*) spectrum of 9


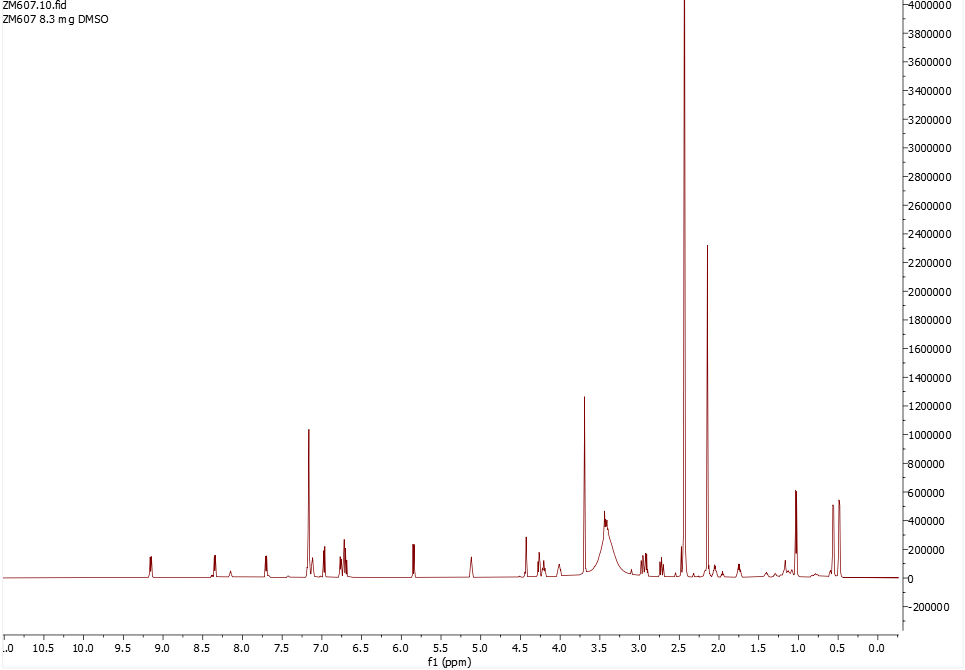


Figure S62. ^1^H NMR (600 MHz, DMSO-*d_6_*) spectrum of 10


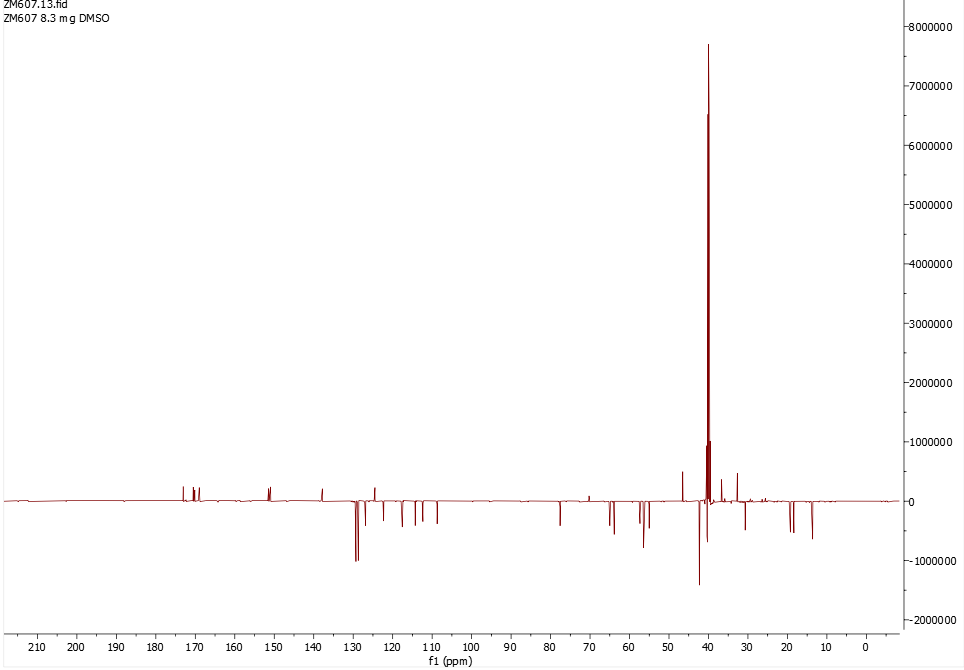


Figure S63. DEPTQ NMR (150 MHz, DMSO-*d_6_*) spectrum of 10


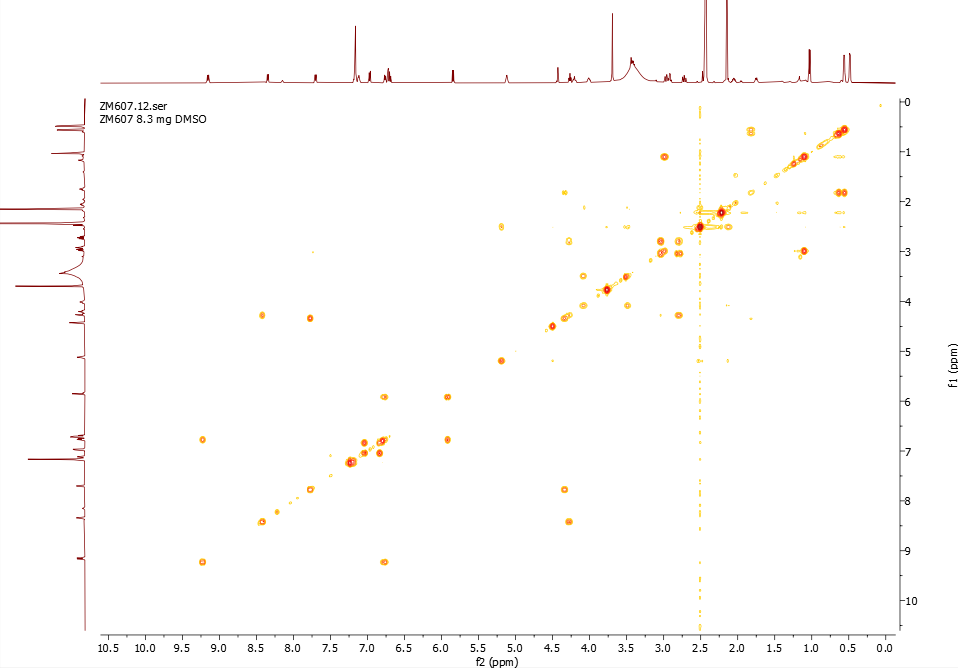


Figure S64. COSY (600 MHz, DMSO-*d_6_*) spectrum of 10


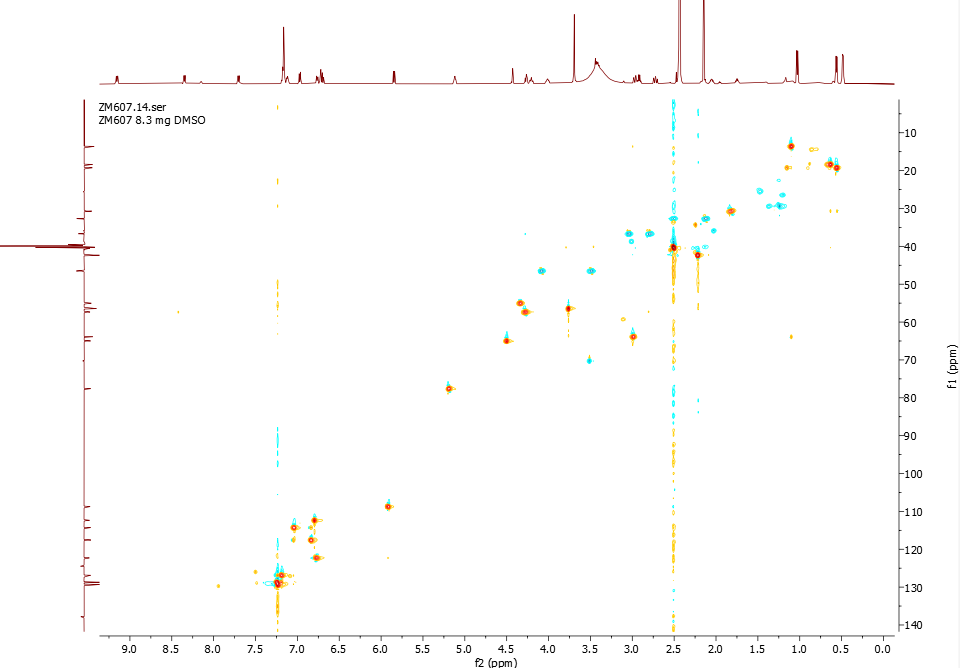


Figure S65. HSQC (600 MHz, DMSO-*d_6_*) spectrum of 10


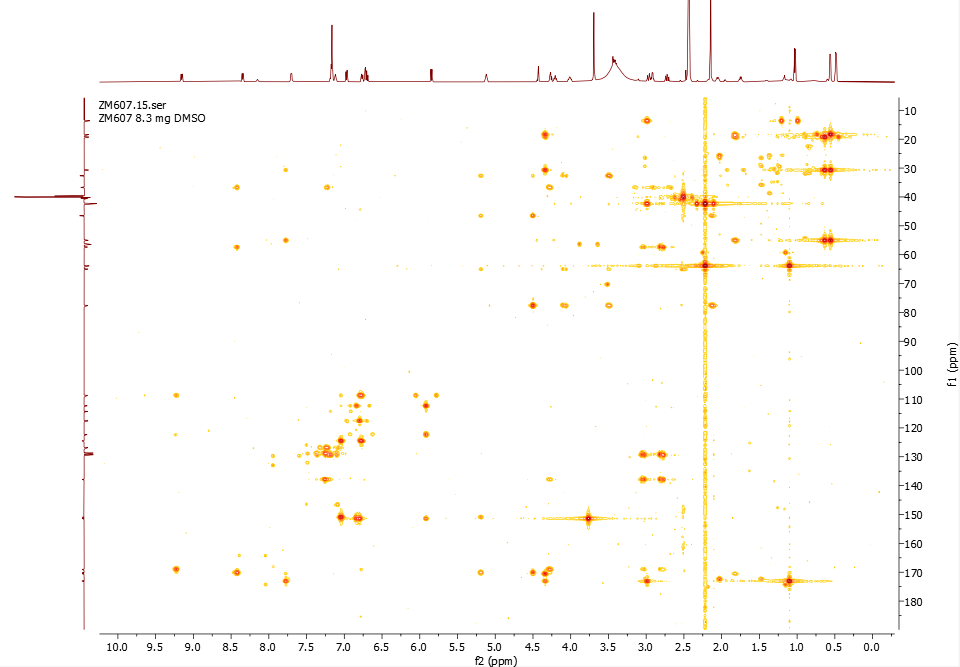


Figure S66. HMBC (600 MHz, DMSO-*d_6_*) spectrum of 10

**
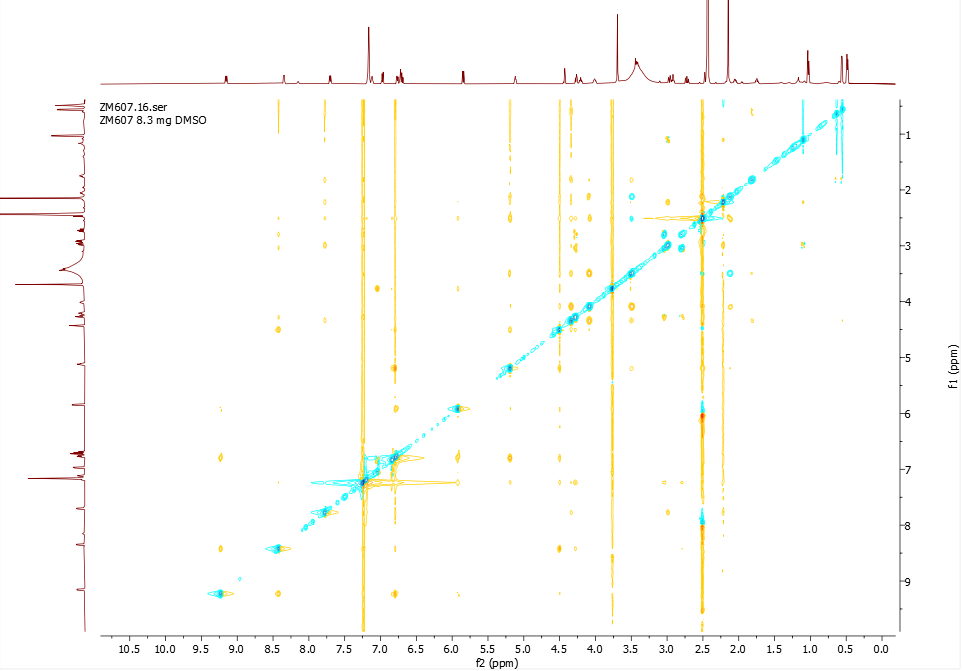
**

Figure S67. ROESY (600 MHz, DMSO-*d_6_*) spectrum of 10


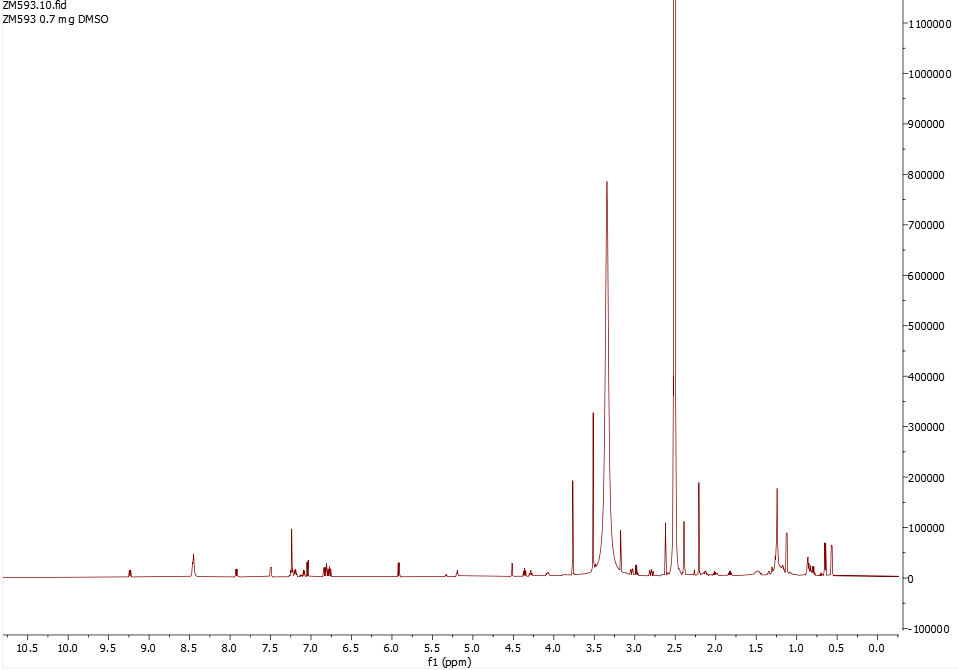


Figure S68. ^1^H NMR (600 MHz, DMSO-*d_6_*) spectrum of 11


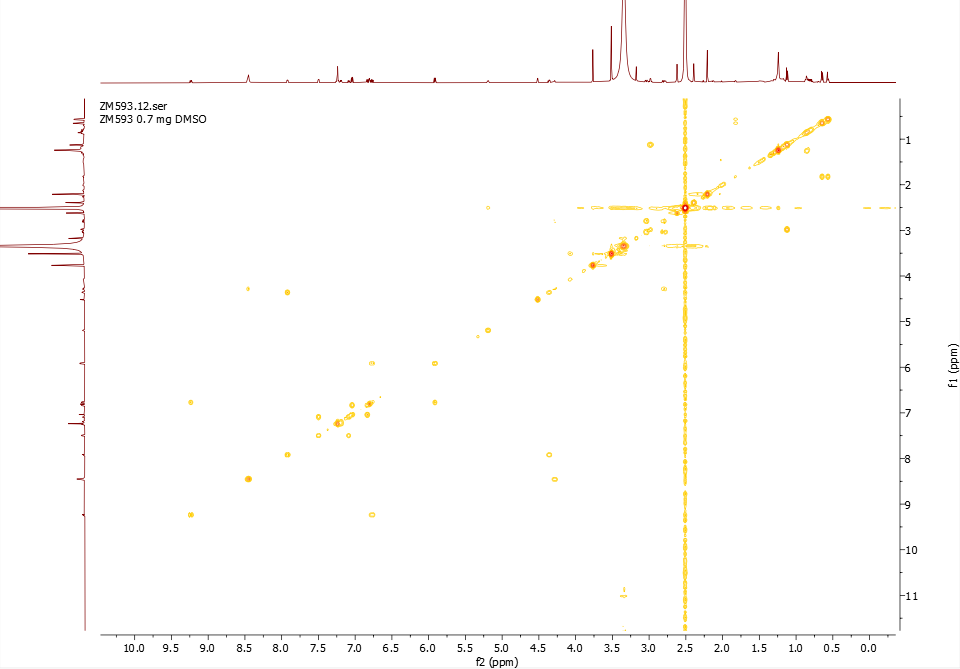


Figure S69. COSY (600 MHz, DMSO-*d_6_*) spectrum of 11


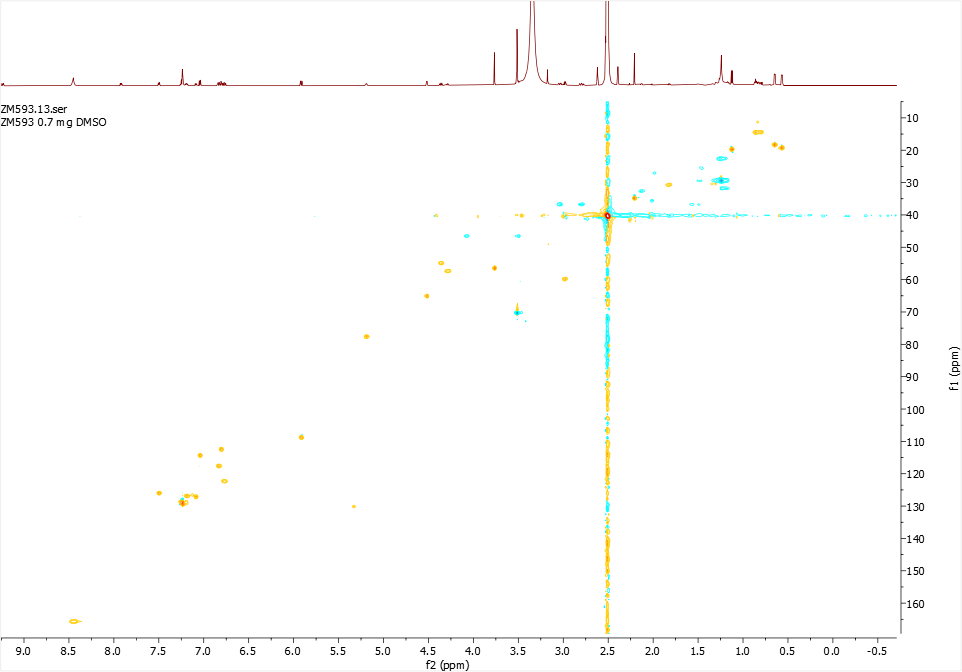


Figure S70. HSQC (600 MHz, DMSO-*d_6_*) spectrum of 11


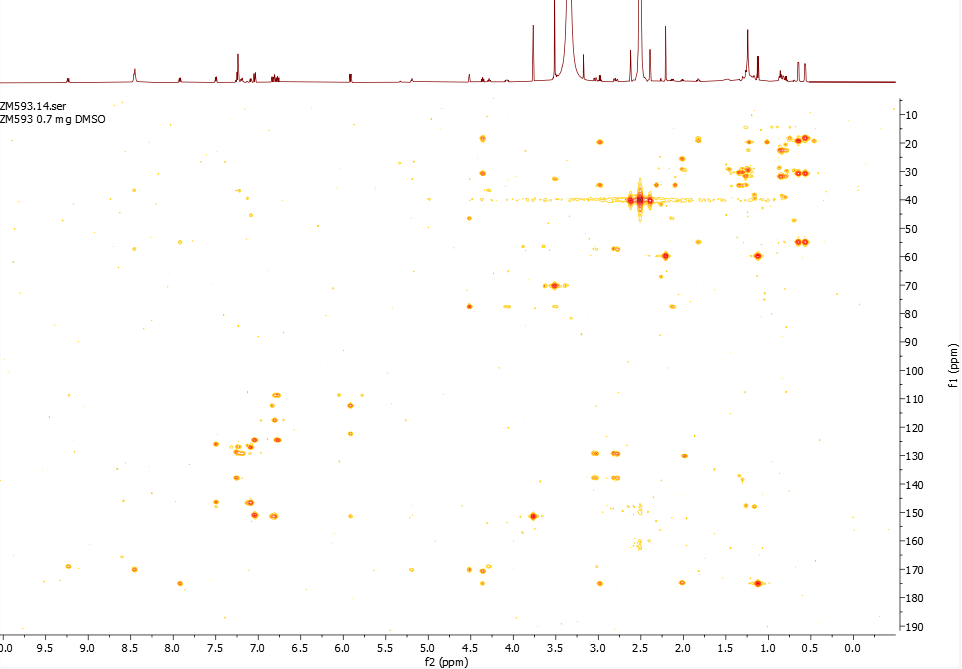


Figure S71. HMBC (600 MHz, DMSO-*d_6_*) spectrum of 11

**
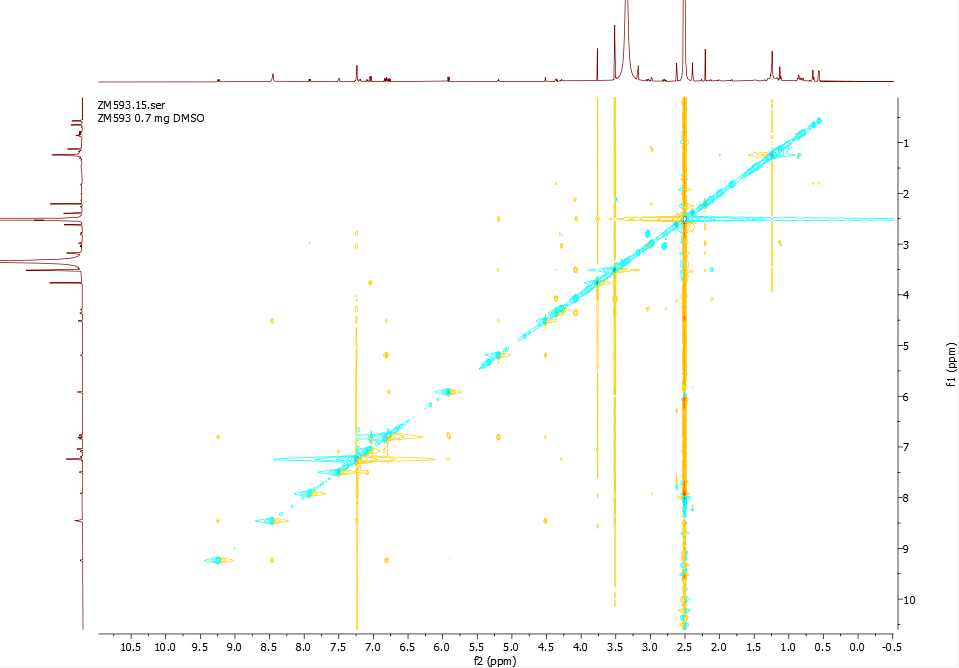
**

Figure S72. ROESY (600 MHz, DMSO-*d_6_*) spectrum of 11


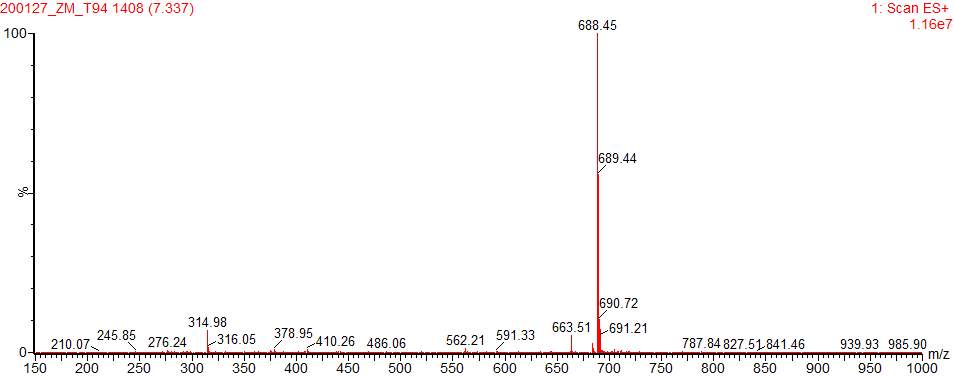


Figure S73. ESIMS spectrum of 12


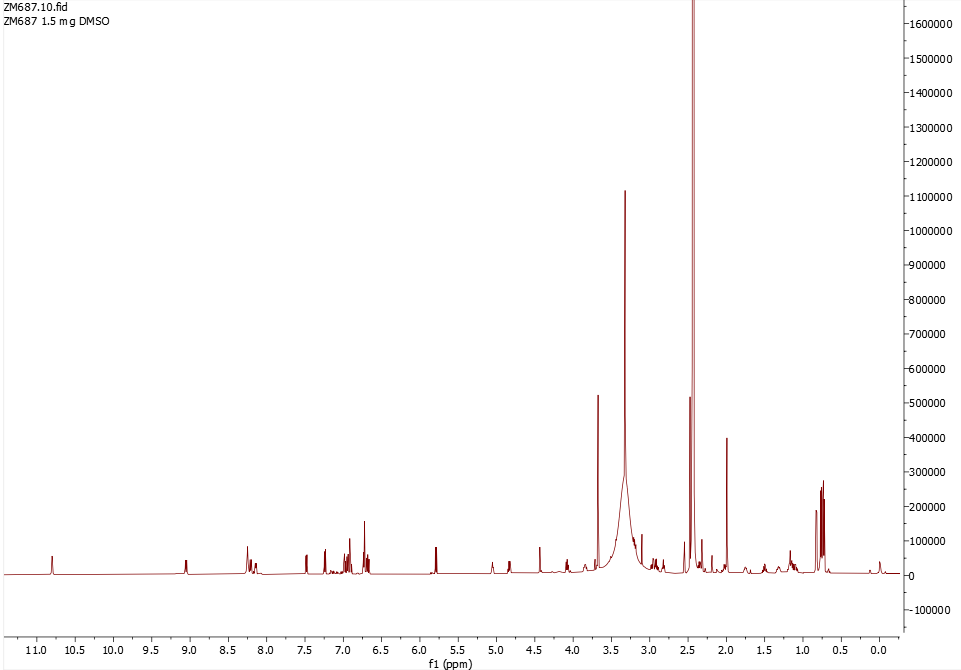


Figure S74. ^1^H NMR (600 MHz, DMSO-*d_6_*) spectrum of 12


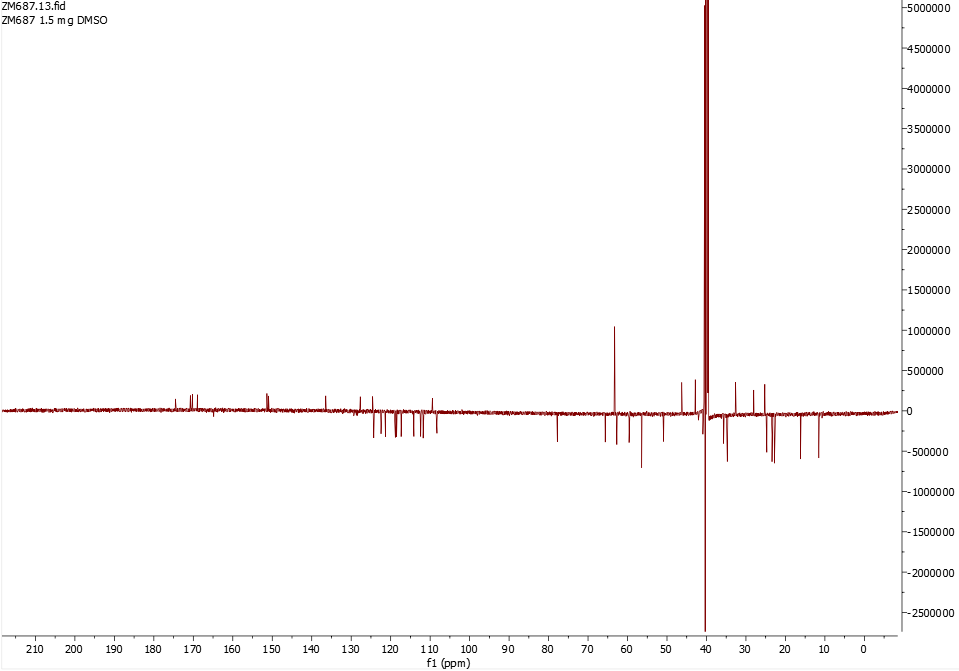


Figure S75. DEPTQ NMR (150 MHz, DMSO-*d_6_*) spectrum of 12


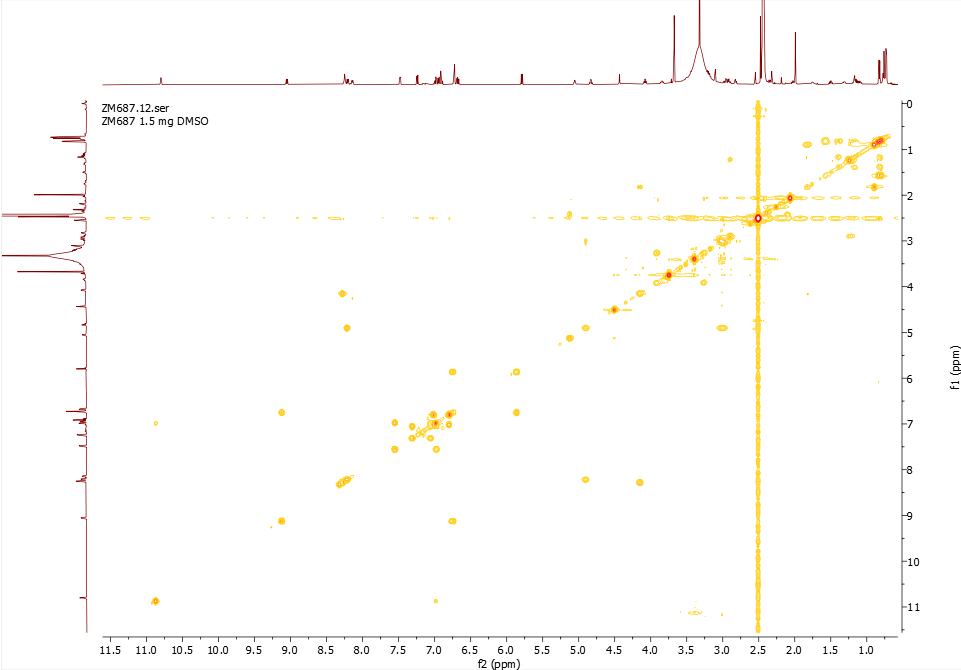


Figure S76. COSY (600 MHz, DMSO-*d_6_*) spectrum of 12


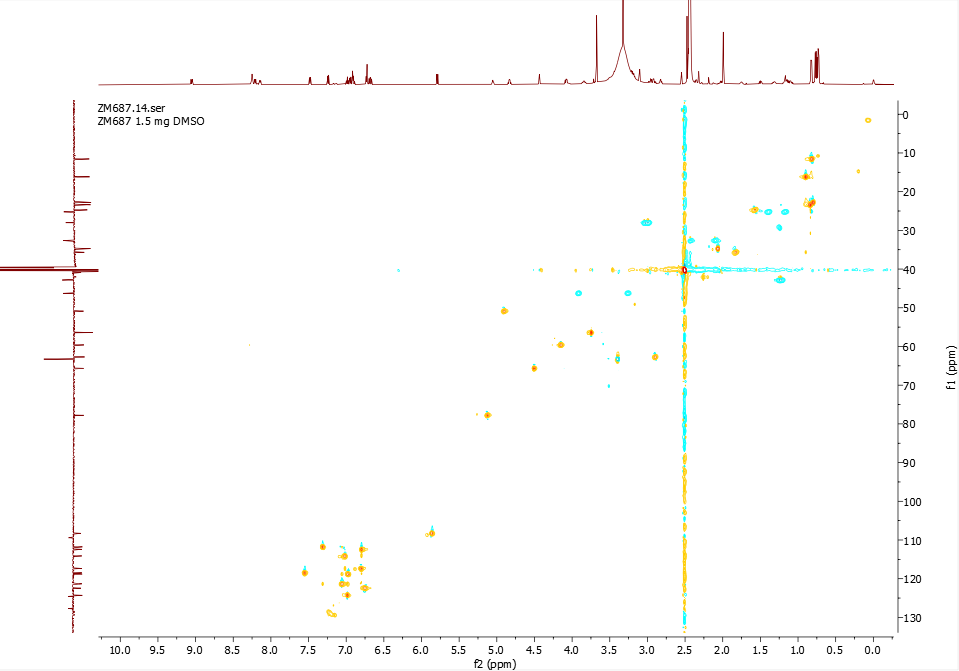


Figure S77. HSQC (600 MHz, DMSO-*d_6_*) spectrum of 12


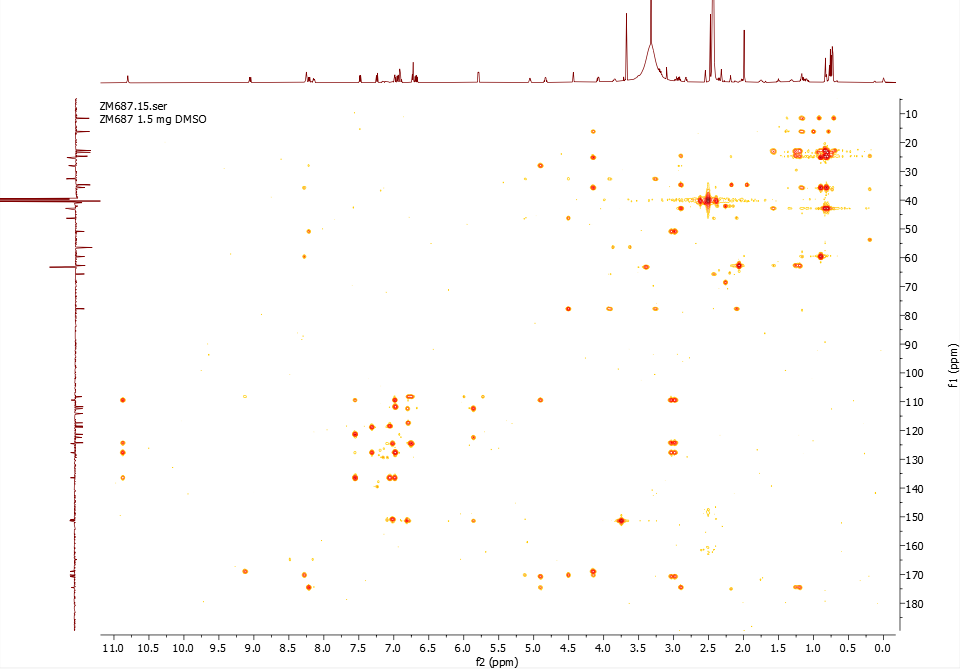


Figure S78. HMBC (600 MHz, DMSO-*d_6_*) spectrum of 12

**
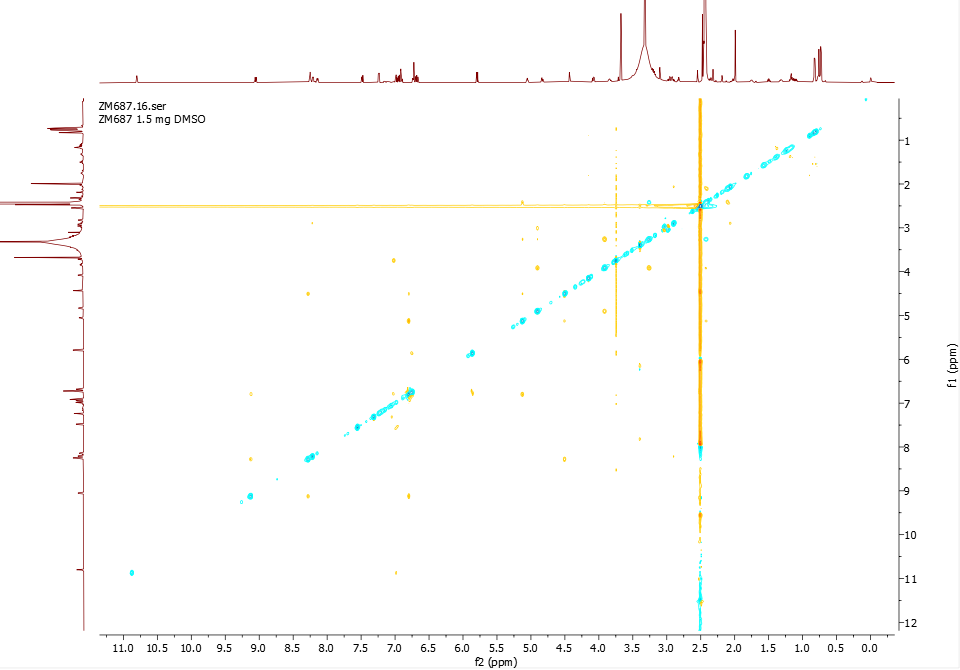
**

Figure S79. ROESY (600 MHz, DMSO-*d_6_*) spectrum of 12


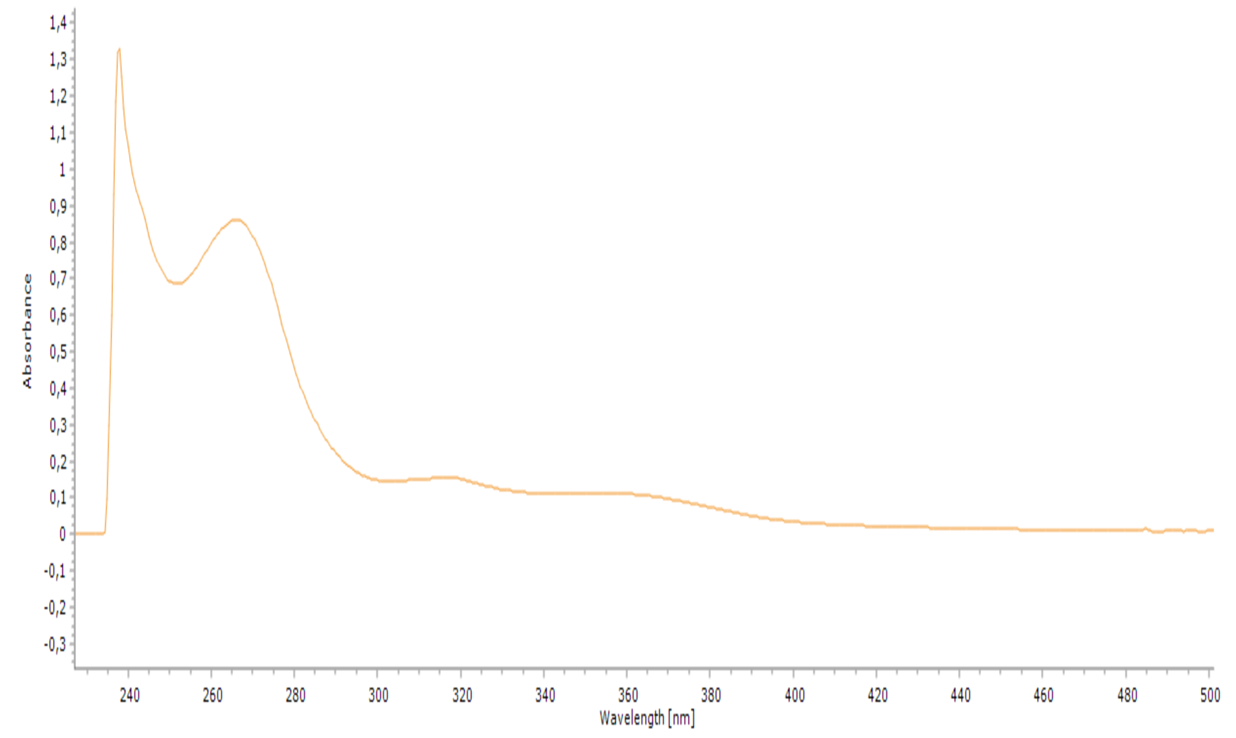


Figure S80. UV spectrum of 13


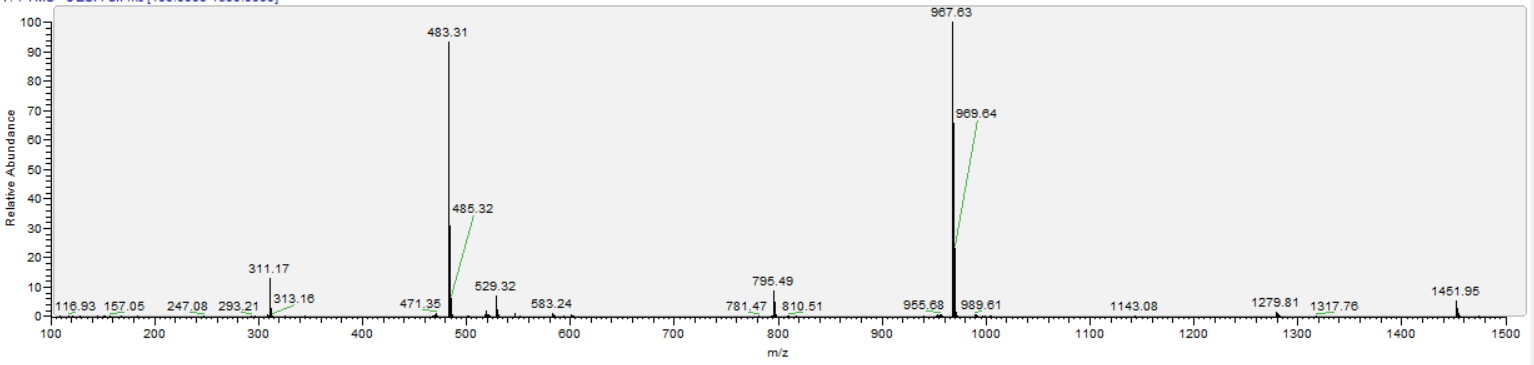


Figure S81. HRESIMS spectrum of 13


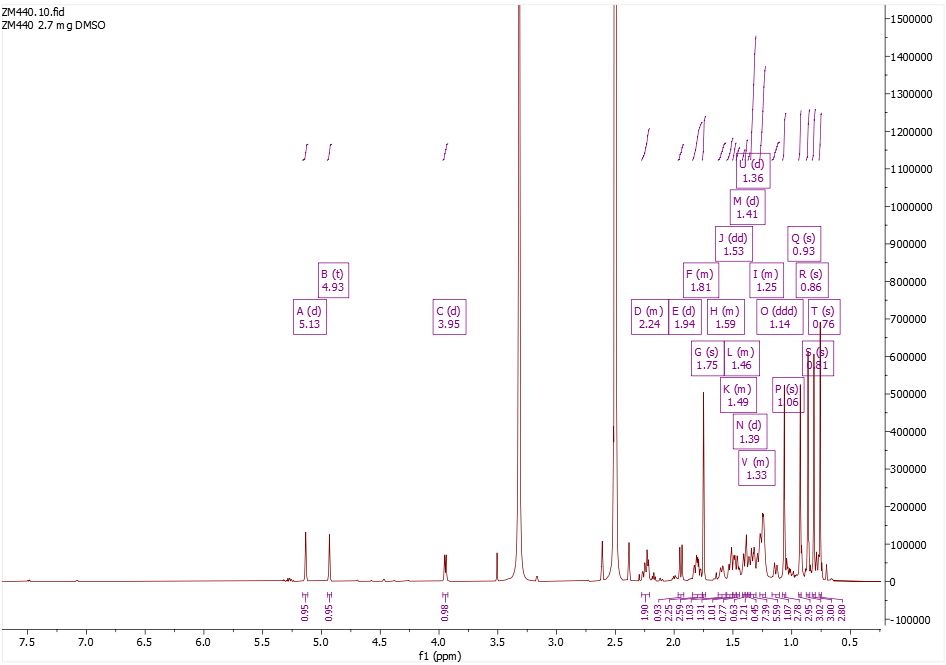


Figure S82. ^1^H NMR (600 MHz, DMSO-*d_6_*) spectrum of 13


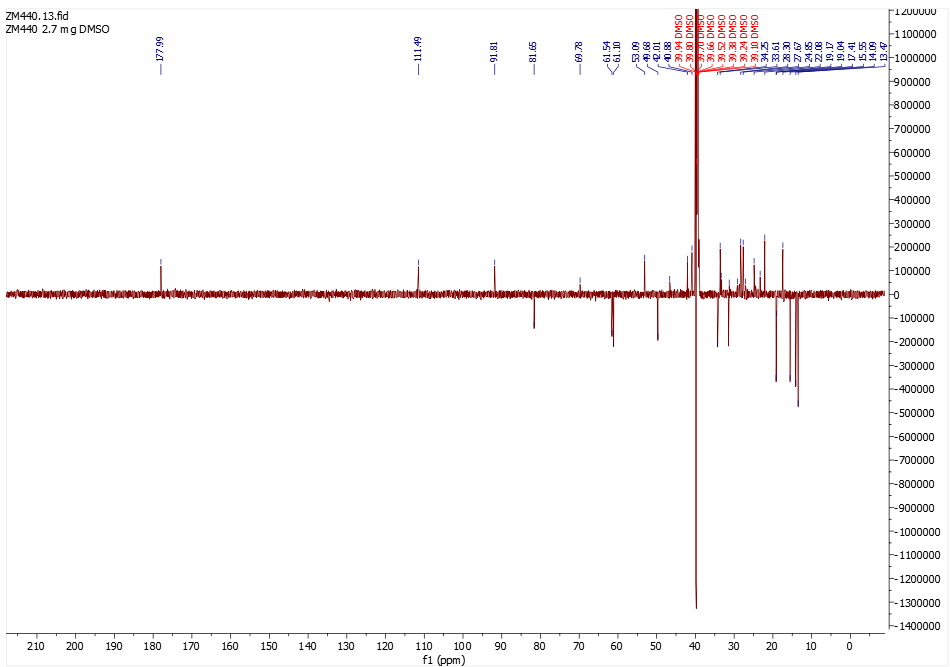


Figure S83. DEPTQ NMR (150 MHz, DMSO-*d_6_*) spectrum of 13


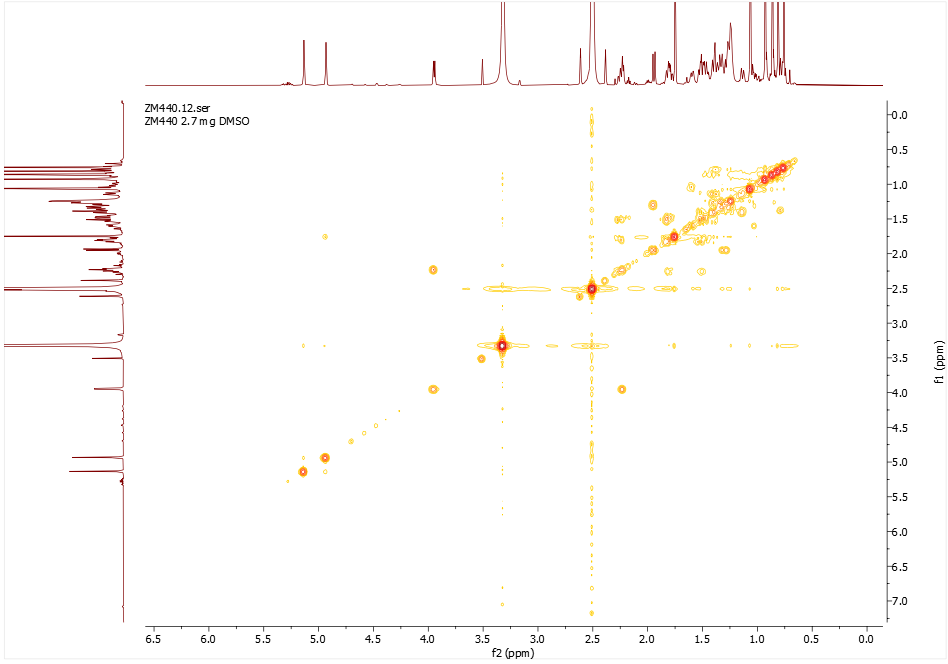


Figure S84. COSY (600 MHz, DMSO-*d_6_*) spectrum of 13


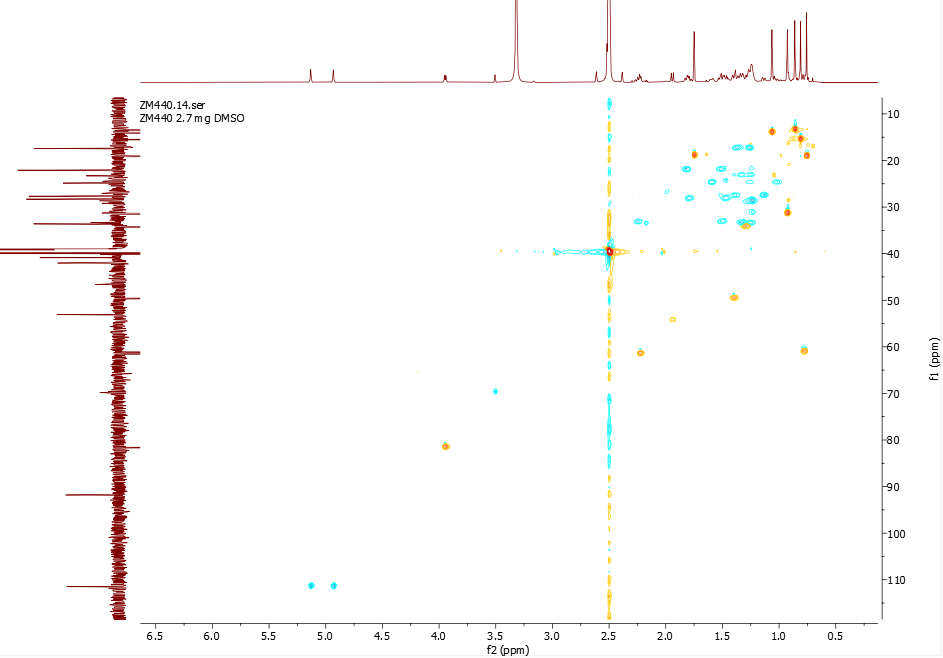


Figure S85. HSQC (600 MHz, DMSO-*d_6_*) spectrum of 13


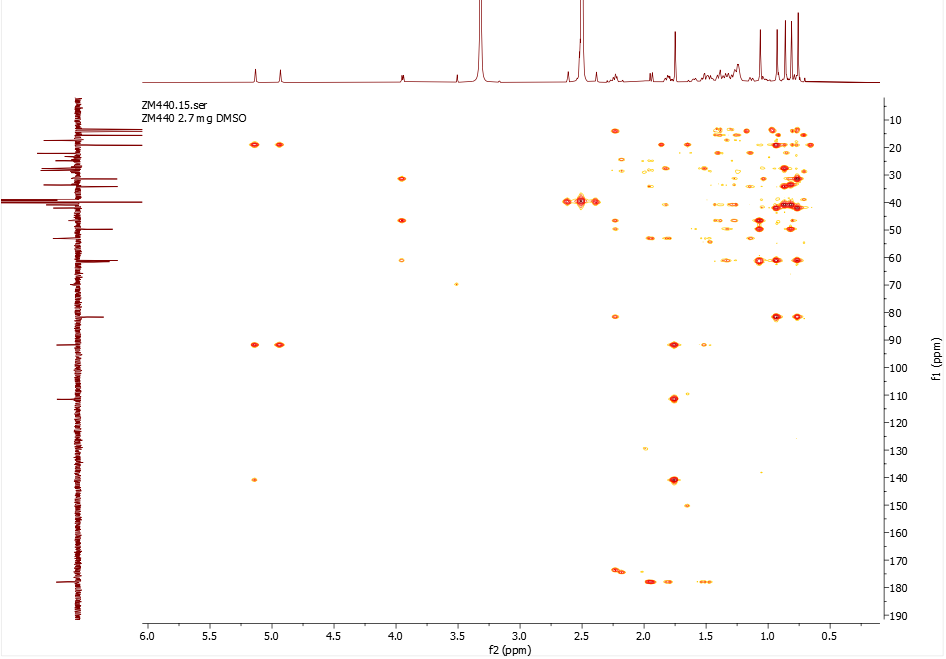


Figure S86. HMBC (600 MHz, DMSO-*d_6_*) spectrum of 13


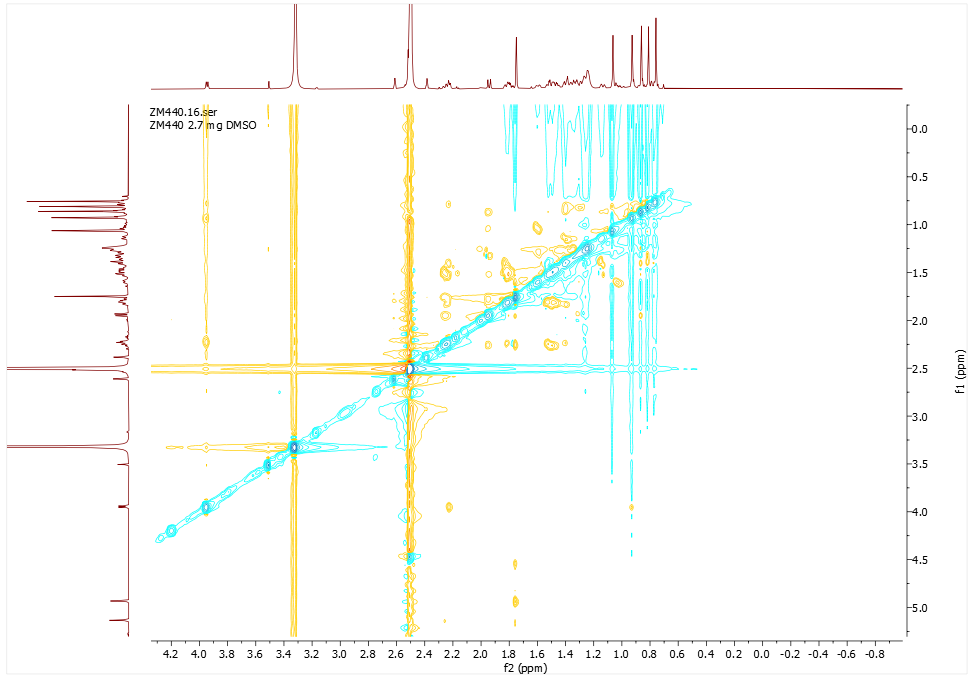


Figure S87. ROESY (600 MHz, DMSO-*d_6_*) spectrum of 13


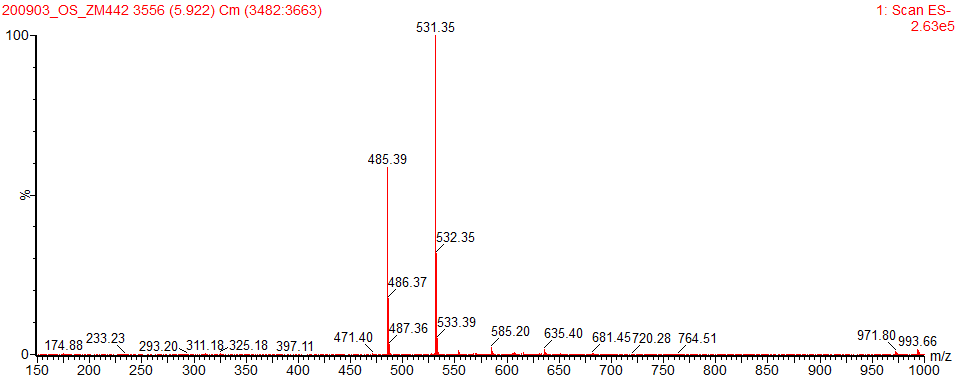


Figure S88. ESIMS spectrum of 14


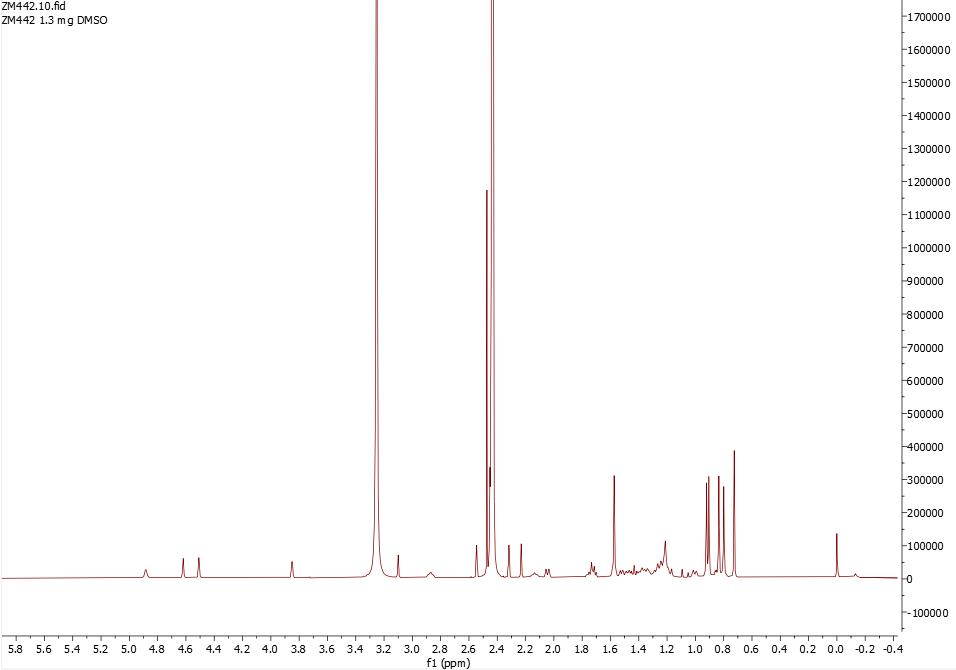


Figure S89. ^1^H NMR (600 MHz, DMSO-*d_6_*) spectrum of 14


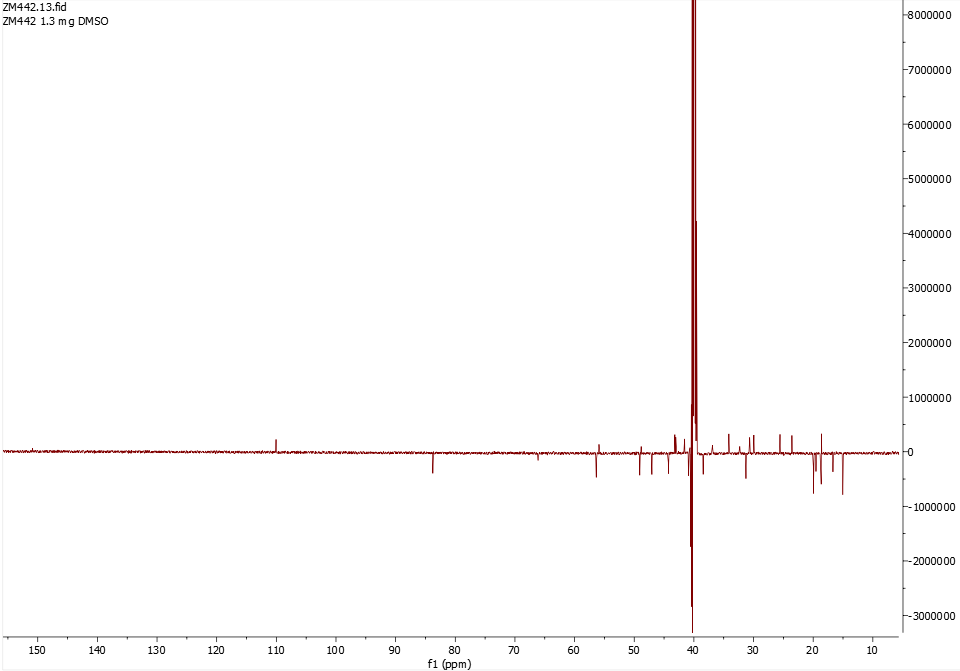


Figure S90. DEPTQ NMR (150 MHz, DMSO-*d_6_*) spectrum of 14


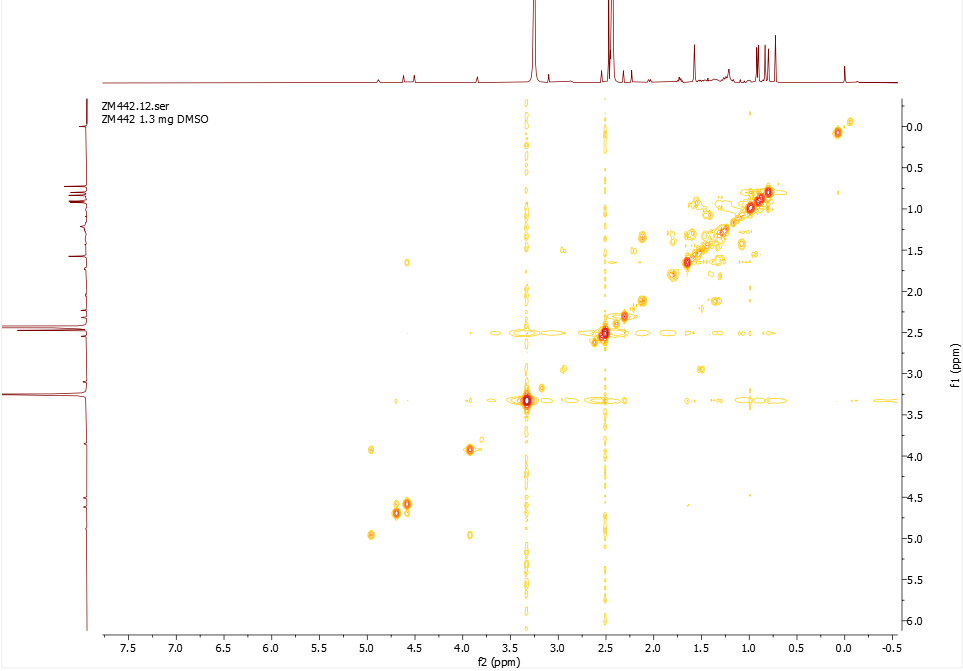


Figure S91. COSY (600 MHz, DMSO-*d_6_*) spectrum of 14


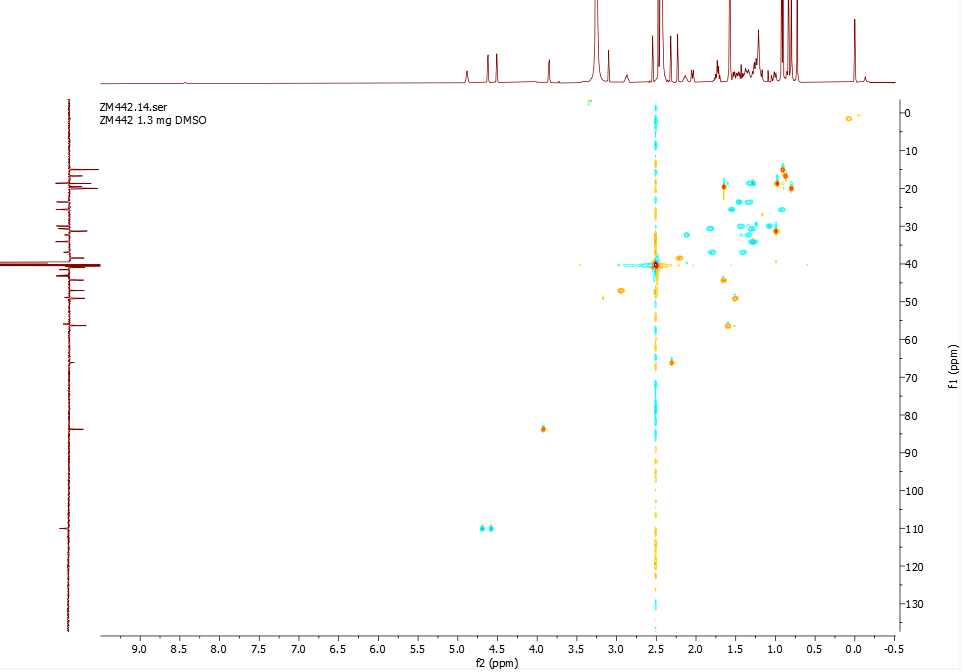


Figure S92. HSQC (600 MHz, DMSO-*d_6_*) spectrum of 14


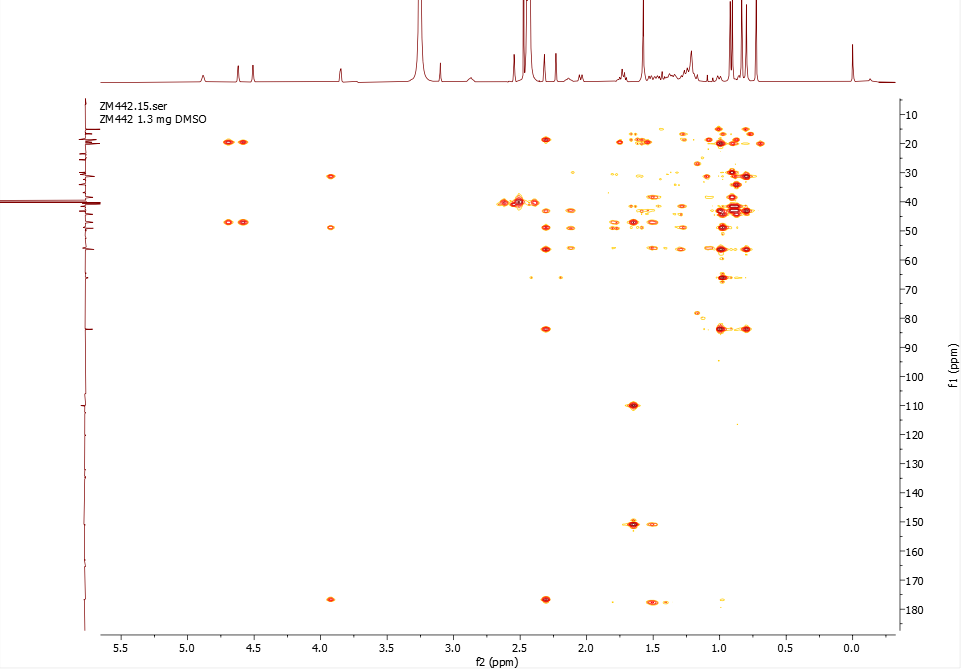


Figure S93. HMBC (600 MHz, DMSO-*d_6_*) spectrum of 14

**
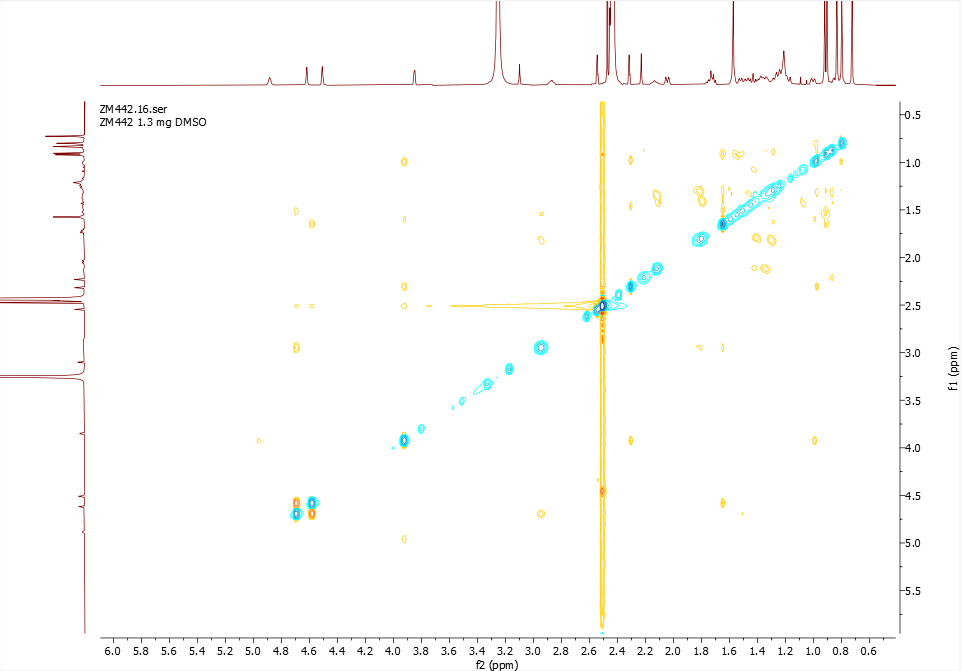
**

Figure S94. ROESY (600 MHz, DMSO-*d_6_*) spectrum of 14


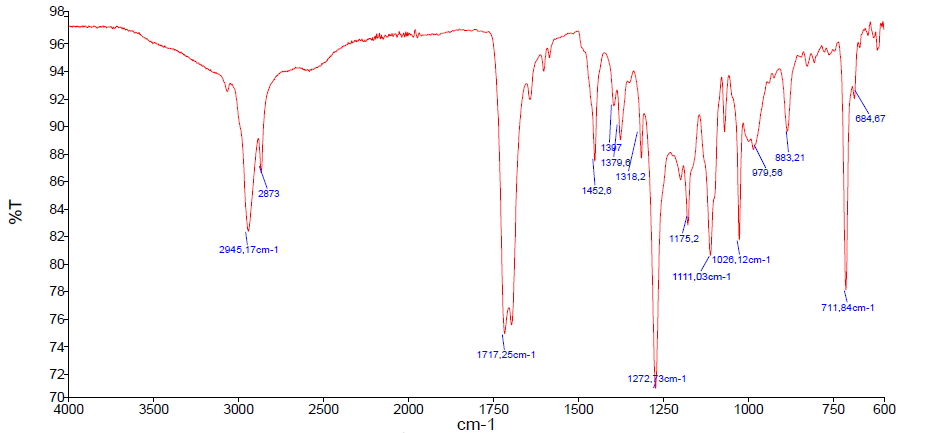


Figure S95. IR spectrum of 15


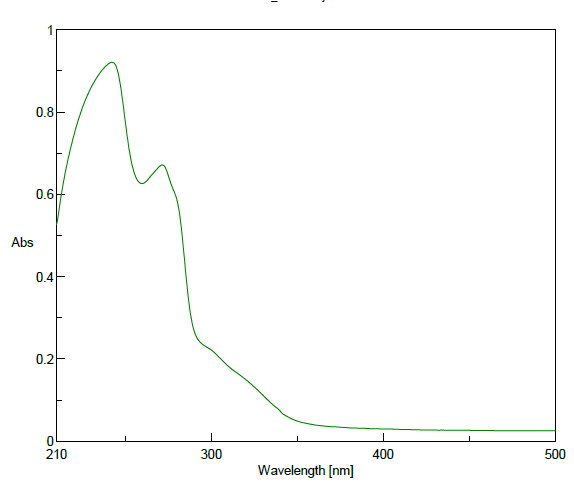


Figure S96. UV spectrum of 15


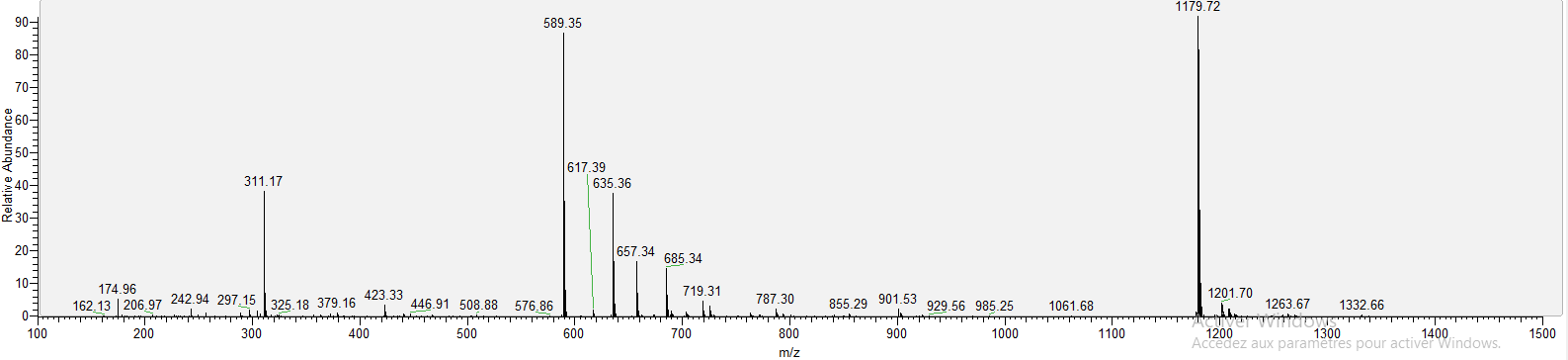


Figure S97. HRESIMS spectrum of 15

**
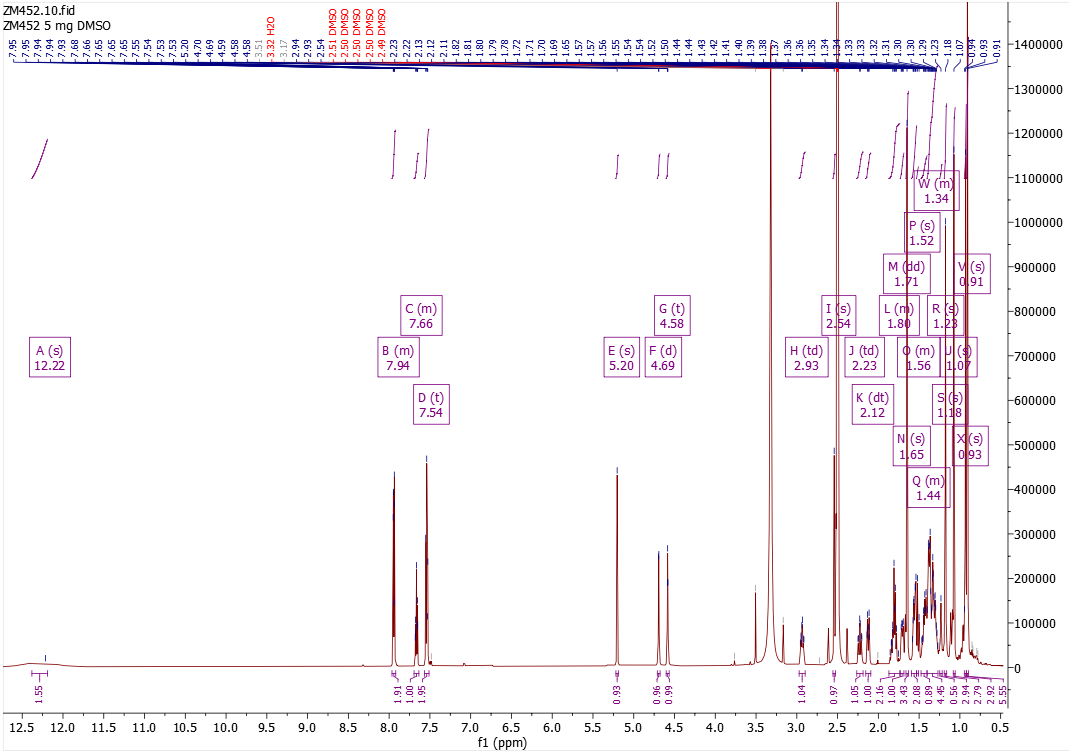
**

Figure S98. ^1^H NMR (600 MHz, DMSO-*d_6_*) spectrum of 15

**
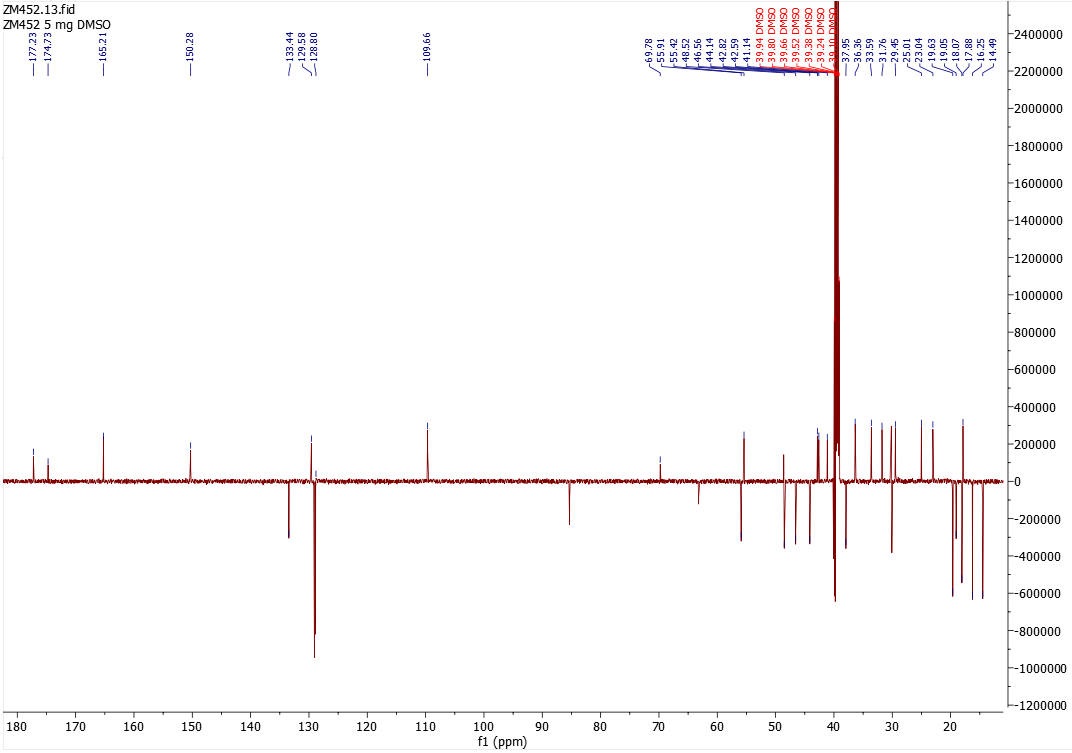
**

Figure S99. DEPTQ NMR (150 MHz, DMSO-*d_6_*) spectrum of 15

**
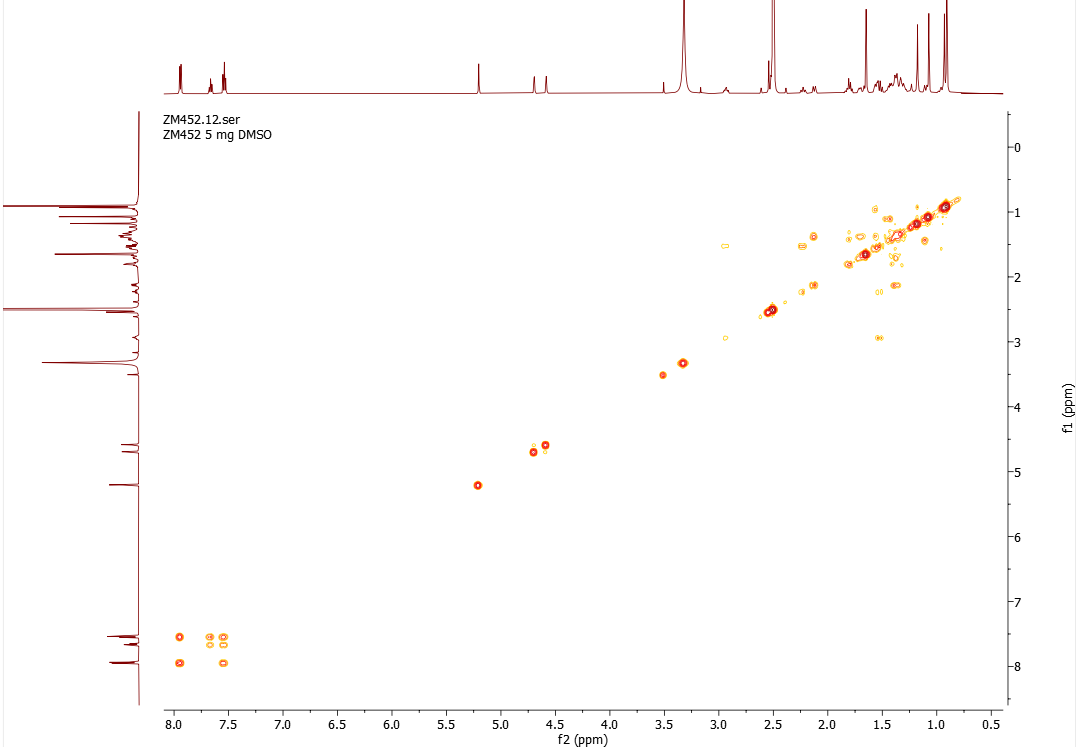
**

Figure S100. COSY (600 MHz, DMSO-*d_6_*) spectrum of 15

Figure S101. HSQC (600 MHz, DMSO-*d_6_*) spectrum of 15

Figure S102. HMBC (600 MHz, DMSO-*d_6_*) spectrum of 15

**Figure S103.** ROESY (600 MHz, DMSO-*d_6_*) spectrum of **15**

Figure S104. IR spectrum of 16

Figure S105. UV spectrum of 16

Figure S106. HRESIMS spectrum of 16

Figure S107. ^1^H NMR (600 MHz, DMSO-*d_6_*) spectrum of 16

Figure S108. DEPTQ NMR (150 MHz, DMSO-*d_6_*) spectrum of 16

**Figure S109.** COSY (600 MHz, DMSO-*d_6_*) spectrum of **16**

Figure S110. HSQC (600 MHz, DMSO-*d_6_*) spectrum of 16

Figure S111. HMBC (600 MHz, DMSO-*d_6_*) spectrum of 16

Figure S112. ROESY (600 MHz, DMSO-*d_6_*) spectrum of 16

Figure S113. ESIMS spectrum of 17

Figure S114. ^1^H NMR (600 MHz, DMSO-*d_6_*) spectrum of 17

Figure S115. COSY (600 MHz, DMSO-*d_6_*) spectrum of 17

Figure S116. HSQC (600 MHz, DMSO-*d_6_*) spectrum of 17

Figure S117. HMBC (600 MHz, DMSO-*d_6_*) spectrum of 17

Figure S118. ROESY (600 MHz, DMSO-*d_6_*) spectrum of 17

Figure S119. ESIMS spectrum of 18

Figure S120. ^1^H NMR (600 MHz, DMSO-*d_6_*) spectrum of 18

Figure S121. DEPTQ NMR (150 MHz, DMSO-*d_6_*) spectrum of 18

Figure S122. COSY (600 MHz, DMSO-*d_6_*) spectrum of 18

Figure S123. HSQC (600 MHz, DMSO-*d_6_*) spectrum of 18

Figure S124. HMBC (600 MHz, DMSO-*d_6_*) spectrum of 18

Figure S125. ROESY (600 MHz, DMSO-*d_6_*) spectrum of 18

Figure S126. ESIMS spectrum of 19

Figure S127. ^1^H NMR (600 MHz, DMSO-*d_6_*) spectrum of 19

Figure S128. DEPTQ NMR (150 MHz, DMSO-*d_6_*) spectrum of 19

**Figure S129.** COSY (600 MHz, DMSO-*d_6_*) spectrum of **19**

Figure S130. HSQC (600 MHz, DMSO-*d_6_*) spectrum of 19

Figure S131. HMBC (600 MHz, DMSO-*d_6_*) spectrum of 19

Figure S132. ROESY (600 MHz, DMSO-*d_6_*) spectrum of 19
